# Operating CRISPR/Cas12a in a complex nucleic acid sequence background

**DOI:** 10.1093/nar/gkag390

**Published:** 2026-04-30

**Authors:** Henning Hellmer, Thomas Mayer, Lea Bauersachs, Friedrich C Simmel

**Affiliations:** Department of Bioscience, School of Natural Sciences, Technical University of Munich, Garching, D-85748, Germany; Department of Bioscience, School of Natural Sciences, Technical University of Munich, Garching, D-85748, Germany; Department of Bioscience, School of Natural Sciences, Technical University of Munich, Garching, D-85748, Germany; Department of Bioscience, School of Natural Sciences, Technical University of Munich, Garching, D-85748, Germany

## Abstract

Since their discovery, CRISPR-Cas systems have been widely applied in areas ranging from genome editing to biosensing, owing to their specific, RNA-guided target recognition. Their performance in complex biological environments has been extensively studied, particularly to optimize guide RNA (gRNA) design and minimize off-target cleavage. Here, we focus on the kinetic inhibition of the interaction between Cas12a—a Class 2, Type V effector—and its target, caused by interference from non-cognate background nucleic acids. This effect is particularly relevant for sensing applications in complex mixtures or cellular contexts, where genome- and transcriptome-derived sequences may impede CRISPR-Cas activity. Using *in vitro* assays under defined conditions, we systematically examine the influence of background single-stranded RNA and double-stranded DNA (dsDNA) on reaction kinetics. We find that both the purine-to-pyrimidine ratio and the GC content of the gRNA seed region significantly affect kinetic inhibition by background polynucleotides. gRNAs with low GC content and a high purine fraction in the seed region were least affected by background sequences. A gRNA with high uracil content in the seed region exhibited particularly strong inhibition in the presence of a dsDNA background. Experiments with dCas12a-based gene activation in living cells indicate that our *in vitro* findings may also be relevant for *in vivo* applications.

## Graphical Abstract

**Graphical Abstract:**
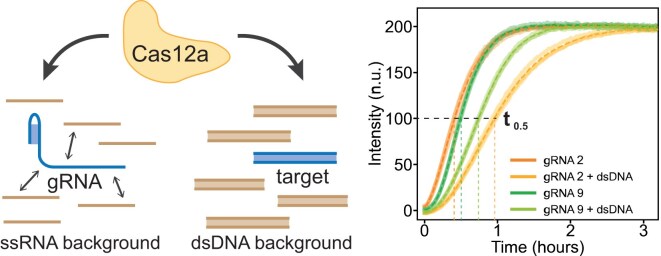

## Introduction

In recent years CRISPR/Cas technology has become a standard approach for the manipulation of genomic DNA. A wide range of CRISPR-associated (Cas) proteins have been discovered, which carry out diverse functions by binding to and chemically processing DNA or RNA molecules, or both [1]. Among the most popular proteins are the CRISPR-associated endonucleases Cas9 and Cas12a, which have already found widespread use in genome editing and a plethora of other applications [2, 3]. The reason for the huge impact of CRISPR-associated nucleases is the fact that they are directed toward their target sequences by an RNA guide (gRNA), which may be a single-stranded RNA or a complex of several RNAs [2, 3]. This makes Cas proteins, in a sense, “sequence-programmable” [4]. Upon incorporation of the gRNA into the Cas endonuclease, the resulting ribonucleoprotein complex scans potential target nucleic acids for complementary sequences, and after target recognition cleaves them. Besides sequence-specific cutting of the target, some RNA-guided Cas endonucleases show additional unspecific cleavage of non-cognate nucleic acids after target recognition. In its biological context this has been interpreted as a suicide reaction toward phage infection [5, 6].

In the present work we focus on Cas12a, a Class 2, Type V nuclease which targets double-stranded DNA (dsDNA) and cleaves the double strand at the binding site (*cis-*cleavage) [3]. Compared to the more widely used Cas9, Cas12a employs shorter, single-stranded gRNAs. In addition, Cas12a can process precursor RNA strands that contain its cognate handle sequence, thereby trimming longer gRNA transcripts into functional guides [3]. Binding to its target sequence triggers a conformational change which activates the Cas protein’s cleavage domain [7]. Activated Cas12a also exhibits unspecific “*trans* ”-cleavage of single-stranded DNA (ssDNA) in the proximity of its nuclease domain, which can be utilized as a readout for target recognition and activation. While *cis-*cleavage is a single turnover process, *trans*-cleavage occurs multiple times, with a reported turnover rate of 17 s^−1^ [5]. Short ssDNA reporter molecules, labeled with a fluorophore at one end and a quencher at the other, are cleaved by Cas12a’s trans-cleavage activity. This cleavage separates the fluorophore from the quencher, resulting in an increase in fluorescence that serves as a detectable signal—a readout technique that has been coined DETECTR [5, 8].

Because of its unique potential for precise genetic manipulation, CRISPR/Cas technology holds significant promise in biomedicine, with genome editing emerging as one of its most far-reaching applications [9]. In such settings, however, it is essential that genomic edits are both predictable and reliable within the complex biological environment of a living organism. One important and extensively studied aspect of CRISPR/Cas systems is, therefore, their specificity in targeting only the intended genomic locus. To achieve target precision, engineered Cas variants with enhanced binding fidelity have been developed, along with AI-guided tools that predict potential off-target sites across the genome [10, 11].

While off-target effects and cleavage kinetics have been studied extensively, the specific impact of competing RNA molecules and non-target DNA on target cleavage kinetics has received comparatively less attention. CRISPR/Cas target recognition in cells involves a series of steps that must occur before DNA cleavage can proceed [12]. First, the expressed Cas protein must bind the transcribed gRNA, forming a functional Cas–gRNA complex. This complex then searches the genome for its target sequence [13, 14]. Two distinct types of interactions may influence the kinetics of these early steps. During and shortly after transcription, unbound gRNA may interact with other RNAs in the nucleus, potentially leading to sequestration or the formation of secondary structures that interfere with Cas protein binding [15]. Once the gRNA is successfully incorporated into the Cas protein, the target search begins. In this phase, the Cas–gRNA complex scans the DNA for a protospacer adjacent motif (PAM)—a short sequence motif essential for initial binding and local DNA unwinding. For Cas12a, the PAM sequence is 5′-TTTV-3′, where V represents A, C, or G. Upon PAM recognition, the complex interrogates the adjacent DNA sequence for complementarity to the gRNA spacer. When sufficient complementarity is not found, the complex dissociates and resumes scanning. The first 5-6 nucleotides (nt) adjacent to the PAM—the so-called seed region—have been found to be particularly critical for initiating hybridization. When sufficient complementarity is established, the complex undergoes a conformational transition into the R-loop state, in which the non-target DNA strand (sharing the same sequence as the gRNA spacer) is displaced. This structural rearrangement positions the nuclease domain for cleavage, enabling site-specific cutting of the target DNA. In Cas12a, this activation also triggers *trans*-cleavage activity [16–18].

The stability of the seed region plays a critical role in determining the dissociation rate of the Cas–gRNA–DNA complex. This implies that the sequence composition of the gRNA, and consequently the target, influences the duration for which the Cas–gRNA remains bound at an off-target DNA locus containing a PAM sequence, where complementarity is probed. Stronger interactions between gRNA and off-target DNA are expected to result in prolonged binding, slowing down the kinetics of the intended reaction. It has, in fact, been shown that CRISPR-Cas off-target binding can be well understood in the context of nucleic acid hybridization kinetics [19].

Using single molecule tracking experiments [13, 14, 20, 21], the kinetics of the target search process have been investigated for the Cas9 protein in greater detail, and to some extent for Cas12a [16]. In both cases, the search process is a combination of three-dimensional diffusion between DNA strands or strand regions and one-dimensional diffusion along the DNA once the Cas9 binds to a PAM sequence. Dwell times at a PAM site are on the order of one second for Cas9 and much shorter for Cas12a, but increase substantially with more PAMs [21, 22] or an increasingly matching target sequence [16, 22] adjacent to the initial binding site. Other studies stated that interactions between the Cas12a effector and DNA are similar to those for Cas9 [23, 24].

It has previously been noted that the recognition and binding of CRISPR-associated effectors to their target sites, which requires both a PAM and a complementary sequence, resembles a toehold-mediated strand displacement (TMSD) process [25, 26]. TMSD is a widely employed mechanism in nucleic acid nanotechnology, where an invading strand displaces an incumbent strand from a nucleic acid duplex through a branch migration process. Recent studies have shown that TMSD processes are impacted by the sequence composition of the participating nucleic acid strands in various ways. For instance, the sequence of the invader strand dictates its interactions with background sequences, which slow down the binding of the invader to the target [27]. Moreover, once strand displacement is initiated, it has been found that individual steps of the branch migration process are highly affected by the sequence composition, particularly in RNA–DNA hybrid strand displacement processes [28, 29].

In the present study, we investigate how the sequence composition of gRNAs influences the kinetics of the intended Cas–gRNA–target interaction *in vitro* within complex sequence environments. Specifically, we employed synthetic pools of background dsDNA containing a variety of sequence motifs. These include sequences with partial complementarity to the gRNA but lacking a PAM site, sequences with complementarity only in regions distal to the seed, and other combinatorial configurations. We further analyzed the effects of background pools consisting of random dsDNA or ssRNA, each pool potentially covering the full spectrum of possible gRNA target motifs.

To further explore the impact of gRNA composition, we also employed sequences based on different three-letter alphabets and varied both the purine-to-pyrimidine ratio and GC content. This enabled us to probe sequence design principles that were previously shown to influence DNA/RNA hybridization and branch migration kinetics in the context of TMSD. Based on the quantitative analysis of our data, we identified sequence features that correlate with reduced kinetic inhibition. We hypothesize that the found sequence features mitigate off-target interactions and unfavorable secondary structures, thereby facilitating more efficient target recognition. We finally examined a CRISPR/Cas system designed based on these features also in living HEK cells. Our results show that the gRNA most strongly affected by a random pool background *in vitro* is also associated with significantly reduced CRISPR gene activation in our cellular model system.

## Materials and methods

### Materials

We used NUPACK for all sequence design and analysis tasks [30, 31]. Template oligos for gRNA transcription were ordered as polyacrylamide gel electrophoresis-purified dsDNA at Integrated DNA Technologies (IDT), targets were ordered as lab-ready dsDNA in 1× IDTE buffer. Templates and primers for the preparation of random ssRNA background and dsDNA background were ordered as lab-ready ssDNA in 1× IDTE buffer. Cas12a (Alt-R™ AsCas12a Ultra nuclease) was ordered from IDT, reaction buffer (NEBuffer 3.1) was ordered from New England Biolabs (NEB). Single-stranded, fluorophore-quencher labeled reporter strands (5′-FAM-TTATT-BHQ1-3′) were ordered from IDT. Q5 mastermix for polymerase chain reaction (PCR) reactions as well as *in vitro* transcription (IVT) components, namely T7 RNA polymerase, rNTPs, and RNA polymerase buffer were ordered from NEB. For DNA digestion, we used TURBO DNA-free™ kits from Thermo Fisher. For RNA and DNA purification, Zymo Research RNA or DNA Clean & Concentrator kits were used. Gene fragments for cloning were ordered at Twist Biosciences, short DNA oligos were ordered from IDT in 1× IDTE buffer. BsmBI, T4 ligase, and T4 polynucleotide kinase were purchased from NEB. Bacterial host cells (*Escherichia coli* DH5alpha strain) and LB medium were ordered from Thermo Fisher. For cell culture, HEK293T cells were acquired from ATCC. Dulbecco’s modified Eagle’s medium and Opti-MEM media, FBS, DPBS, and Trypsin-ethylenediaminetetraacetic acid were ordered from Merck. Antibiotic-antimycotic and Lipofectamine™ 2000 were purchased from Thermo Fisher.

### *In vitro* assay overview

All *in vitro* experiments were performed following the same procedure. The final assay composition contained 1x reaction buffer (NEBuffer 3.1), 60 nM AsCas12a, 150 nM gRNA, 6.7 nM dsDNA target, 200 nM ssDNA reporter, and either 2 µM of RNA background strands, 200 nM of DNA background strands, or no background. The mixture was brought to a final volume of 15 µl with water. A mastermix containing all constituents except for target DNA and guide RNA (gRNA) was prepared. The mastermix was pipetted into a Corning® low volume, non-binding plate, and the gRNA and the target were added last. The plate was sealed, samples spun down, and the plate was put into either a BMG Labtech FLUOstar® Omega or CLARIOstar® plate reader with the temperature set to 37°C; excitation and emission filters were preset to 485 nm (15 nm bandwidth) and 530 nm (20 nm bandwidth), respectively. After starting the measurement, fluorescence intensity data were collected each minute for 16 h. The data were transferred to an Excel file and subsequently plotted and analyzed using custom Python scripts (provided in the Zenodo repository). For every measurement a negative control without target DNA was conducted. Experiments were performed in technical triplicates on the same plate.

### Preparation of guide RNAs and polynucleotide backgrounds

#### Guide RNAs

gRNAs were transcribed from dsDNA oligos containing the T7 RNAP promoter sequence (provided in the Zenodo repository). Transcription was carried out following the standard NEB protocol, with an incubation time of 16 h to maximize RNA yield. Transcripts were treated with TURBO^TM^ DNase according to the rigorous treatment protocol, followed by purification with the RNA Clean & Concentrator kit (Zymo Research).

#### dsDNA background

The dsDNA background used in our experiments was only partially random, as we had to balance sequence randomness against synthesis yield and cost. To our knowledge, commercially available dsDNA cannot be ordered with fully randomized sequences—only ssDNA is available in this format. As a workaround, we ordered 200-nucleotide-long ssDNA oligonucleotides containing two fixed primer-binding regions at the 5′ and 3′ ends (5′-P1–N168–P2-3′), resulting in 168 central nucleotides with randomized composition. The sequences of the constant flanking regions—which were designed to not contain a PAM for Cas12a—are provided in the Zenodo repository. To reduce synthesis costs, we amplified these ssDNA templates by PCR, ensuring that each resulting dsDNA molecule exists in multiple copies. Even though the presence of the fixed primer sequences limits the overall sequence diversity, there are still 168 random bases per strand, corresponding to 4^168 ^≈ 10 ^100^ different possibilities for the sequence. The number N of different sequences before performing the PCR can be calculated via ${\mathrm{N\ }} = {\mathrm{\ }}{{{\mathrm{N}}}_{\mathrm{A}}}{\mathrm{\ }} \cdot {\mathrm{c}}( {{\mathrm{ssDNA}}} ){\mathrm{\ }} \cdot {\mathrm{V}},$ where ${{N}_A}$ is the Avogadro constant, $\mathrm{ c}( {\mathrm{ssDNA}} )$ the concentration and $V$ the volume of the ssDNA solution, which in our case results in ${\mathrm{N}} = {\mathrm{\ }}6.02 \cdot {{10}^{23}}\frac{1}{{{\mathrm{mol}}}} \cdot 2 \cdot {{10}^{ - 6}}{\mathrm{\ }}\frac{{{\mathrm{mol}}}}{{\mathrm{L}}} \cdot 1 \cdot {{10}^{ - 6}}{\mathrm{\ L\ }} = {\mathrm{\ }}1.2 \cdot {{10}^{12}}$ different sequences to start with. For the PCR we added the forward and reverse primer to a final concentration of 2 µM, for which we obtained the best yield of the full-length product without generating shorter dsDNA strands due to undesired primer binding.

#### ssRNA background

In the case of single-stranded RNA (ssRNA) background, we were able to produce fully random ssRNA strands. To this end, we ordered ssDNA with the 183 random nucleotides, followed by the reverse complementary T7 promoter sequence. Before transcription, we added an oligo with the T7 promoter sequence that can bind to its reverse complement and added the Q5 mastermix and performed a primer extension reaction, to produce a double stranded template for IVT. As for the gRNAs, the transcription was performed according to the standard NEB protocol with transcription time set to 16 h to maximize the output yield. The IVT output RNA with a length of 183 bases was processed with the TURBO DNA-free™ Kit and subsequently cleaned up using the Zymo Research RNA Clean & Concentrator kit.

### Kinetic data analysis

Kinetic data were analyzed using Excel and Python. The data are provided in a time versus intensity format. We performed technical triplicates and included negative controls in every measurement. For normalization and better comparability, the background value (obtained from the negative controls) was subtracted from the intensity values, and reactions normalized to a final value of 200 (which is the nanomolar concentration of reporter in every measurement). For comparison of the kinetics with (w) and without (w/o) background sequences, we extracted the time to reach 50% completion (${{{\mathrm{t}}}_{0.5}}$) for each sample with each condition by identifying the earliest time point at which the fluorescence intensity exceeded 50% of the maximum signal observed for that sample. To compare the effect of background DNA on kinetic performance, we computed all possible pairwise ${{{\mathrm{t}}}_{0.5}}$ ratios via ${\mathrm{r}} = \frac{{{{{\mathrm{t}}}_{0.5}}( {{\mathrm{w\ background}}} )}}{{{{{\mathrm{t}}}_{0.5}}( {{\mathrm{w}}/{\mathrm{o\ background}}} )}}$ between replicates of the DNA-containing condition and its matching DNA-free control (i.e. 3 × 3 = 9 comparisons per group). The mean of these nine ratios was taken as the relative $t_{ 0.5}$, and the standard deviation was used to represent the variability across comparisons.

### Symbolic regression

We applied symbolic regression using the PySRRegressor from the pysr library to identify algebraic expressions that relate features of our spacer sequences to the measured kinetic slowdowns. Sequence features from the seed region that appeared to correlate with the kinetic ratio, such as the purine fraction, uracil fraction, or GC fraction, were, together with the kinetic ratio, imported into a data frame for further analysis. We used the PySRRegressor to minimize the mean squared error with a custom element-wise loss function, and selected models based on the best loss score. Models with different depth and complexity were compared to identify the most salient sequence features governing the kinetic slowdown. Additional information, including example fits, is provided in the Supplementary Information. The full code and datasets are available in the accompanying Zenodo repository.

### ODE model

For the model used in the discussion around Fig. 6, we first fitted the data from a DETECTR experiment using gRNA 2 in the absence of a nucleic acid background. To this end, we normalized the fluorescence data to span a range corresponding to 0–200 nM target concentration. We used scipy.solve_ivp as the ODE solver and manually adjusted ${{{\mathrm{k}}}_{{\mathrm{ON,T}}}}$ and ${{k}_2}$ within a physically reasonable range until agreement with the data were found. These two parameters were then fixed. Using the dataset for gRNA 2 in the presence of a random DNA background, the remaining two rate constants of Model 2 (${{{\mathrm{k}}}_{{\mathrm{ON,B}}}},{\mathrm{\ }}{{{\mathrm{k}}}_{{\mathrm{OFF}},{\mathrm{B}}}}$) were determined in the same way. The reported values are consistent with typical rates for such processes.

### *In cellulo* experiments

For *in cellulo* experiments, the readout strategy had to be adapted. We chose a denCas12a-VPR-based CRISPR activation system that results in GFP expression in response to target binding. Plasmids for the gRNA and a denCas12a-VPR construct, respectively, were transiently transfected alongside a target plasmid coding for GFP.

#### Plasmid design and cloning

The plasmid coding for constitutive expression of denAsCas12a-VPR was available in-house. The gRNA plasmids were cloned via Golden Gate assembly [32]. As backbone, we used a plasmid with the required cutting sites (BsmBI) downstream of a basic U6 promoter. An additional U6 + 27 sequence and the gRNA sequence were designed in Benchling and ordered as single-stranded oligonucleotides with four-base overlaps. After hybridization, the two resulting duplexes and the backbone were joined together in an assembly reaction following standard procedures and supplemented with T4 polynucleotide kinase to phosphorylate the ordered DNA strands. For the target plasmid, longer gene fragments were designed. These included a CRISPR target site for the respective gRNA sequence followed by a minimal promoter in front of a GFP gene. The designed DNA duplexes were appended on each side with BsmBI cutting sites. As a backbone, a fragment of a plasmid was amplified via PCR, introducing complementary BsmBI restriction sites. This part introduced a bovine growth hormone polyadenylation (*bgh*-PolyA) signal after the GFP gene upon correct assembly. All plasmid sequences are listed in the Zenodo repository.

### Cell culture and qPCR

The performance of CRISPR-mediated transcriptional activation using different gRNAs was evaluated in a cellular context using HEK293T cells cultured under standard conditions. Cells were seeded into 48-well plates at a density of 60 000 cells per well. Twenty-four hours after seeding, cells were transfected with three plasmids encoding the target construct, the gRNA, and the denCas12a–VPR activator. After an additional 24 h, the culture medium was replaced. Total RNA was isolated the following day using the Quick-RNA Miniprep Plus Kit (Zymo Research) according to the manufacturer’s instructions, including the on-column DNase I digestion step. To ensure complete removal of residual genomic DNA, the eluted RNA was subjected to an additional DNase treatment using the TURBO DNA-free™ Kit (Invitrogen) under rigorous conditions according to the manufacturer's instructions with an incubation time of 1 h. Complementary DNA (cDNA) synthesis was performed with 100 ng of RNA per sample using Maxima H Minus First Strand cDNA Synthesis Kit (Thermo Fisher Scientific) according to the manufacturer’s instructions for qPCR use. qPCR was performed using the Luna® Universal qPCR Kit according to the manufacturer’s protocol. Each condition was analyzed using biological triplicates with technical replicates for each sample, using 100 ng of cDNA per reaction. Reactions were carried out on a qTower^3^G (Jena Analytics). The data were analyzed in the qPCRsoft4.1 software (Jena Analytics) using the ΔΔ*C*_t_ method the software. A detailed qPCR protocol following the MIQE guidelines is given in the Supporting Information, including Supplementary Tables S1–S3 with a description of housekeeping genes and primer sequences.

## Results

### Impact of designed background strands on interaction kinetics

The Cas/gRNA target search process has been previously studied in great detail, which has led to a good understanding of how this ribonucleoprotein complex unwinds dsDNA in an RNA–DNA hybrid strand displacement process, during which gRNA displaces the non-target strand and binds to its target sequence.

**Figure 1.**
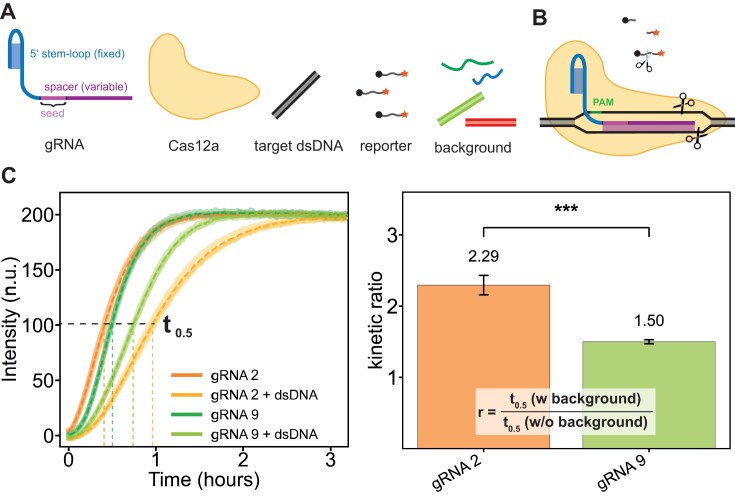
Experimental *in vitro* setup and analysis. (**A**) Individual components of the experiment. The gRNA contains a fixed region required for recognition and uptake by the Cas12a protein. The downstream variable region is called the spacer, with the first eight bases referred to as the seed region. Additionally, we used target dsDNA, a ssDNA reporter labeled with a fluorophore–quencher pair, and varying backgrounds of dsDNA and ssRNA. (**B**) *Trans*-cleavage property. Upon successful binding to the target dsDNA, Cas12a exhibits *trans*-cleavage activity, meaning it non-specifically cleaves surrounding ssDNA. This property can be used to measure system activity in a plate reader experiment. (**C**) Data acquisition and analysis from triplicate measurements (*n* = 3). Left: Fluorescence curves measured for two different gRNAs, with and without dsDNA background. Shown are the means as dotted lines with standard deviations. Although gRNA 2 shows the fastest kinetics without dsDNA background, gRNA 9 is faster in the presence of a dsDNA background. Right: Comparison of the *t*_0.5_ values as a measure of the slowdown caused by the background. Mean values with standard seviations. The gRNA 2 system is slowed down more (*r* = 2.32) than the gRNA 9 system (*r* = 1.50), indicating that gRNA 9 is less affected by the dsDNA background. The difference is significant with *P* = 5.2e-08 (two-sided Welch’s *t*-test).

It has been shown that the first few bases after the PAM recognition site, which are called the seed region, are the most important for stable, initial binding of the Cas-gRNA complex to the dsDNA [3, 7]. For the Cas12a protein, the first 5–6 bases downstream of the PAM are typically regarded as the seed sequence [7, 18]. However, mismatches within the first ≈ 10 bases are known to significantly impair target recognition and binding [33]. For our experiments and designs, we thus regarded the first 8 bases after the PAM as the relevant seed sequence (Fig. 1A). To assess how different Cas12a/gRNA combinations perform in the presence of a background composed of designed or random nucleic acid sequences, we used the DETECTR method for readout (Fig. 1B, cf. Methods).

Upon binding to its target and activation of its nuclease function, Cas12a exhibits a collateral *trans*-cleavage activity on nearby DNA strands. When fluorogenic probes—labeled with both a fluorophore and a quencher—are supplied, this collateral activity leads to a fluorescence increase, which serves as a proxy for successful target recognition and activation. As illustrated in Fig. 1C, DETECTR can be employed as a readout to compare the performance of different gRNA types in the presence or absence of interfering background strands. As demonstrated by the representative fluorescence traces shown, different gRNA types are affected by the background to varying degrees. In the following, we quantify the reaction kinetics by determining the half-time *t*_0.5_ for completion of each process. The effect of the background is then expressed as the ratio *r* of the *t*_0.5_ values in the presence and absence of background strands, respectively.

**Figure 2.**
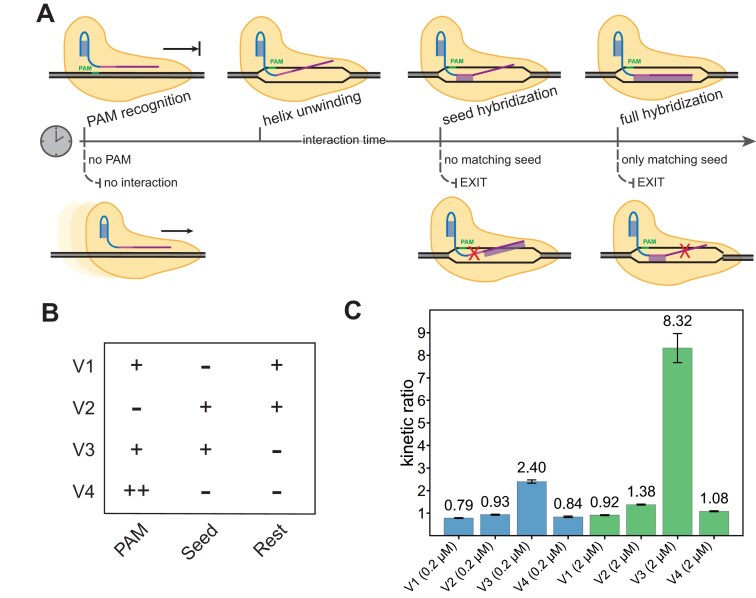
Cas12a target recognition. (**A**) Modes of interaction between the Cas12a enzyme and background DNA and the corresponding interaction time for any DNA locus. Cas12a loaded with a guideRNA is moving along the DNA scanning for PAMs. Upon binding to a PAM, the DNA helix is unwound, and the spacer hybridization takes place starting at the seed region. Only if the complete sequence matches the DNA, R-loop formation is completed, and the endonuclease activity is unlocked. (**B**) Sequence designs for background DNA harboring parts of an additional target sequence to compete with the original target in the assay. V1 carries a PAM and the target sequence missing the seed region, V2 incorporates the complete target but lacks a PAM, V3 only displays a PAM and the seed region, and V4 has several PAMs but no target sequence. (**C**) Relative *t*_0.5_ comparison as a measure of the slowdown caused by the specifically designed DNA background. Shown are the mean values and standard deviations of triplicate measurements (*n* = 3) for background DNA at 0.2 µM and at 2 µM. The greatest slowdown is caused by background version 3, which is in line with the modes of interaction (see A) as present PAM and seed region cause the longest interaction with the background DNA.

To study kinetic inhibition by non-cognate DNA sequences that can hinder the target search process, we designed four different versions (V1–V4) of a dsDNA background. As illustrated in Fig. 2B, V1 contained the PAM sequence, no complementarity for the seed region of the gRNA, but perfect complementarity for the remaining 12 bases. V2 lacked the PAM but contained a sequence perfectly matching the gRNA spacer; V3 harbors a matching PAM and target for the seed sequence but no complementarity in the remaining part. Lastly, V4 has multiple PAMs but is not complementary to the spacer at all. As explained in Fig. 2A, we expected these different types of backgrounds to interfere at different stages of the search process.

Measurements of the kinetic ratio *r* (defined in Fig. 1C) for the different backgrounds are shown in Fig. 2C. A background of dsDNA lacking a PAM sequence (V2) does not appear to inhibit target search by the Cas12a:gRNA complex at all, even though V2 contains a perfectly matching seed region. Moreover, we do not observe any kinetic inhibition by background V1, which includes a PAM, but no sequence matching the seed. This suggests that the Cas12a:gRNA only briefly interacts with PAM sites when the initial bases of the seed region cannot hybridize with the DNA target. For Cas9, similar interactions with isolated PAM sites have previously been shown to last ~1 s [21].

Even in the case of the V4 background sequences, which contain eight PAM sites each, we do not observe any kinetic inhibition within the tested concentration range. This appears to differ from previous observations made for Cas9, suggesting an even faster dissociation of Cas12a from a PAM in the absence of a matching seed region [20].

Notably, a pronounced kinetic slowdown by a factor of ≈ 8 is observed in the case of background V3 (at 2 µM concentration), which contains both a matching PAM and a seed region. Upon encountering the PAM, the Cas12a:gRNA complex can transiently bind to the first eight bases of the dsDNA, reducing the unbinding rate. However, because the remaining 12 bases do not match, this partial interaction is insufficient to initiate R-loop formation and the associated conformational changes required for the activation of *cis-* and *trans*-cleavage [18].

### Impact of random dsDNA and ssRNA background strands on interaction kinetics

**Figure 3.**
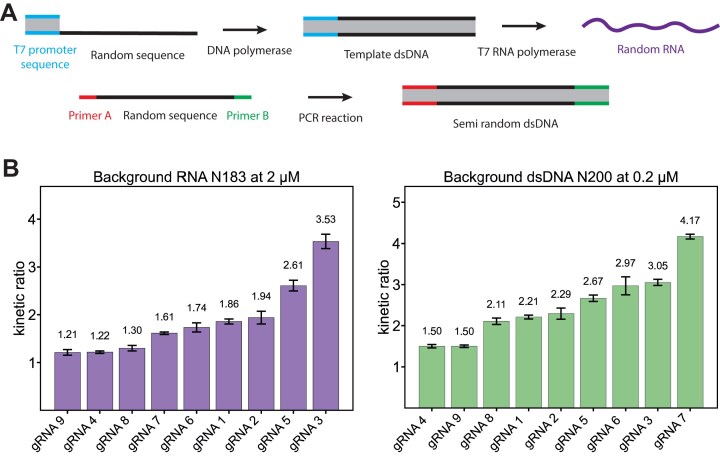
Multiple gRNAs operated in ssRNA or dsDNA background. (**A**) Production of random RNA and semi-random dsDNA. A randomized DNA template with only the T7 promoter sequence fixed is used to first produce the template dsDNA and later transcribe it to random RNA. For DNA, a semi-random ssDNA with two primer sequences flanking the random part is used and multiplied via a PCR reaction. (**B**) First set of gRNAs with RNA or dsDNA background. Shown are the mean values and standard deviations of triplicate measurements (*n* = 3). The dsDNA background (right side) showed comparable slowdown with only a tenth of the concentration of the RNA. A direct comparison between the slowdown caused by RNA and dsDNA shows that there are some gRNAs that are neither heavily influenced by the RNA nor by the dsDNA (e.g. gRNA 9) and others, like gRNA 3, which are slowed down by RNA as well as dsDNA background. Another visual trend is the dependence on the purine-to-pyrimidine ratio, especially for the dsDNA background. The gRNA with a higher purine ratio in the seed region is less influenced by the background.

We next investigated the influence of dsDNA and ssRNA background strands with randomized sequences on Cas12a–gRNA target interactions. The processes used to obtain the dsDNA and ssRNA background are shown in Fig. 3A. As described in the “Materials and methods” section, members of the dsDNA pool contain a 168 bp long randomized sequence domain, which is flanked by constant primer-binding sites required for amplification. The primer sequences were specifically designed to exclude PAM motifs and to not exhibit extensive complementarity to the gRNAs. The primer regions were therefore not expected to significantly influence the experimental outcome.

We worked with randomized dsDNA pools at a total concentration of 200 nM, corresponding to a 30-fold excess relative to the target concentration (6.7 nM). In contrast, for ssRNA we used a concentration of 2 µM, as little to no inhibitory effect was observed at lower concentrations. Due to the substantially weaker kinetic inhibition exerted by ssRNA compared to dsDNA at equivalent concentrations, we hypothesize that ssRNA influences the kinetics mainly by interacting with the gRNA prior to its incorporation into the Cas protein, but not with gRNA sequestered within a Cas12a complex. Such interactions can occur when all components are mixed simultaneously, potentially inhibiting the association of Cas12a with the gRNA.

As shown in Fig. 3B, we tested different types of gRNAs with varying sequence features (listed in Table 1) to explore how their activity is affected by the presence of random nucleic acid backgrounds. With these gRNAs, we evaluated design strategies that had previously been applied to enhance the robustness and predictability of nucleic acid strand displacement reactions [27]. These included gRNA sequences based on restricted three-letter alphabets [3L-A (gRNA 3), 3L-C (gRNA 4), 3L-G (gRNA 5), 3L-T (gRNA 6)] and sequences with varying purine-to-pyrimidine ratios. Additionally, we tested a gRNA (gRNA 1) that had been used previously in our lab [34], along with a version in which its bases were randomly permuted (gRNA 2). Although secondary structure of the gRNA may also influence kinetics, we did not specifically account for it in this study. Instead, the gRNA sequences were designed to avoid strong secondary structures within the spacer sequence, which is a commonly applied design principle [35, 36].

**Table 1.** gRNA spacer sequences used in the experiments

Name	Sequence	Pur:Pyr (Seed)	AU:GC (Seed)	Pur:Pyr	AU:GC
gRNA 1	**CCACUCCC** UAUCAGUGAUAG	1:7	2:6	8:12	10:10
gRNA 2	**CUCAGGUA** UCACUCUAGCAC	4:4	4:4	8:12	10:10
gRNA 3	**UCCCUUGC** CUUCCUCCGUCU	1:7	3:5	7:13	9:11
gRNA 4	**UGAAAGUG** AAUGAUGUGAUG	6:2	5:3	15:5	12:8
gRNA 5	**CUUACAAC** CUAUUCCACCCU	3:5	5:3	8:12	12:8
gRNA 6	**AGCAAAGA** CACCGAACGAAC	7:1	5:3	15:5	12:8
gRNA 7	**UUAUUCUU** UACAAUAUAAGU	1:7	7:1	11:9	17:3
gRNA 8	**UAAGAUAU** UACAAUAUAAGU	5:3	7:1	12:8	16:4
gRNA 9	**UAAAUGAU** AUAUACUUGAUU	5:3	7:1	10:10	17:3
gRNA 10	**CCUUCUAC** UACAAUAUAAGU	1:7	4:4	9:11	15:5
gRNA 11	**CCUGAUAC** UACAAUAUAAGU	3:5	4:4	11:9	15:5
gRNA 12	**ACUGAUAA** UACAAUAUAAGU	5:3	6:2	12:8	15:5
gRNA 13	**UUAUACAU** UACAAUAUAAGU	3:5	7:1	12:9	17:3
gRNA 14	**AAAGAUAA** UACAAUAUAAGU	7:1	7:1	13:7	16:4
gRNA 15	**CAAUAAUG** UAAUUAUAAGUU	5:3	6:2	11:9	17:3
gRNA 16	**AUGUCAUU** AAGAUAAUUAUU	3:5	6:2	10:10	17:3
gRNA 17	**AUGCUAUU** AAGAUAAUUAUU	3:5	6:2	10:10	17:3

The seed sequence is shown in bold font.

Notably, even in the absence of background strands, target cleavage kinetics assessed via DETECTR already showed substantial variation across the different gRNAs, consistent with previous observations [35, 37]. To allow for a meaningful comparison of kinetic inhibition across different conditions, we therefore normalized the cleavage rates by defining a kinetic ratio ${\mathit{r\ }} = \frac{{{{{\mathrm{t}}}_{0.5}}( {{\mathrm{w\ background}}} )}}{{{{{\mathrm{t}}}_{0.5}}( {{\mathrm{w}}/{\mathrm{o\ background}}} )}}$ as above. Our comparison of kinetic ratios in Fig. 3B thus does not focus on the fastest kinetics, but rather on the robustness of each gRNA design in the presence of randomized nucleic acid backgrounds. The data show that the ssRNA background causes less kinetic inhibition than the dsDNA pool, although the effect still depends on the specific gRNA sequence used. Notably, the dsDNA background exerts a stronger inhibitory effect despite being present at only 10% of the RNA concentration. Some gRNAs experience strong inhibition by both dsDNA and RNA (e.g. gRNA 3), while others are largely unaffected by either (e.g. gRNA 9), and some are specifically inhibited by dsDNA alone (e.g. gRNA 7).

Interestingly, our data suggest that both the purine-to-pyrimidine ratio and the GC content play a critical role in determining the extent of kinetic inhibition. To further investigate these effects, we designed a new set of gRNAs (gRNA 10–gRNA 13) with varying purine-to-pyrimidine ratios specifically in the seed region. These included sequences with either variable GC content or constant GC content to disentangle the individual and combined contributions of these two parameters. In addition, we generated further variants with low GC content (gRNA 14–gRNA 17), including a scrambled version (gRNA 16) of the original gRNA 9, to test whether the reduced inhibition observed was sequence-specific or might be a general phenomenon (cf. Table 1).

**Figure 4.**
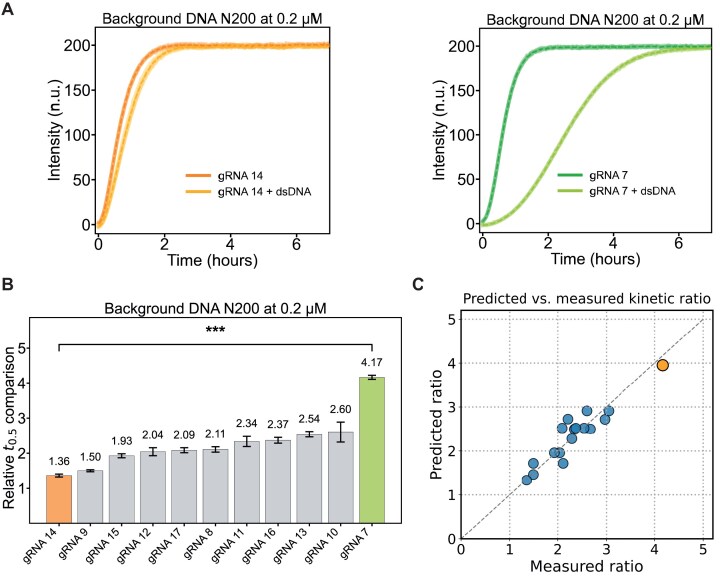
Data sets with varying Pur:Pyr ratio. (**A**) Fluorescence curves for the best performing gRNA 14 (left) and the worst-performing gRNA 7 (right). Shown are the means from triplicate measurements (*n* = 3) as dotted lines with standard deviations. (**B**) Bar plots showing the relative slowdown of the different gRNA versions. Mean values and standard deviations of triplicate measurements (*n* = 3). The difference between gRNA 7 and gRNA 14 at the ends of the spectrum is significant with *P* = 6e-23. (**C**) Symbolic regression analysis of the data. Shown is the comparison of the function ${\mathrm{r}}( {{{{\mathrm{f}}}_{\mathrm{R}}},{{{\mathrm{f}}}_{\mathrm{U}}}} ) = {\mathrm{exp}}[ {{\mathrm{exp}}( {{{{\mathrm{f}}}_{\mathrm{U}}}} ) + ( {{{{\mathrm{f}}}_{\mathrm{U}}}/( {{{{\mathrm{f}}}_{\mathrm{R}}} - 1.0423962} )} )} ]\ $and the experimentally obtained kinetic ratios for all gRNAs tested. gRNA7 is marked in orange.

Given the stronger inhibitory effect observed with the dsDNA background, we focused our experiments on the random dsDNA pool. Using the new set of gRNAs, we repeated our analysis, as shown in Fig. 4. The results indicate a correlation between the purine-to-pyrimidine ratio and the extent of kinetic inhibition: gRNAs with higher purine content are slowed down less in the presence of the dsDNA background. Furthermore, the sequence composition of the seed appears to play a role. In order to capture these trends in a simple mathematical expression, we performed a symbolic regression analysis of the kinetic ratio as a function of various sequence features. We found that the data can be well described by functions of the purine fraction in the seed, ${{f}_R}$, and one additional parameter such as the seed’s uracil, cytosine, or GC fraction (${{f}_U}$, ${{f}_C}$, or ${{f}_{GC}}$). All expressions identified by symbolic regression capture the trend that the kinetic slowdown becomes more pronounced for lower purine content (Supplementary Figs S1 and S2). Furthermore, the slowdown increases with higher uracil, cytosine, or GC content (these parameters are not independent, cf. Supplementary Information). Notably, most of the simpler expressions cannot capture the particularly strong slowdown observed for gRNA7. An example of a model of intermediate complexity based on ${{f}_R}$ and ${{f}_U}$ is shown in Fig. 4C, while a more complex expression that fits the entire dataset is provided in the Supplementary Information (Supplementary Fig. S1E).

### Variation of Cas12a:gRNA activation in cellulo

We next investigated whether the sequence-dependent kinetic inhibition of Cas12a:gRNA interactions observed *in vitro* would also occur in cellulo. Since CRISPR–Cas activation and gene editing take place in the nucleus, both endogenous nuclear RNA and DNA may influence these processes. However, the availability and reactivity of nucleic acids in the nucleus may differ substantially from *in vitro* conditions due to factors such as chromatin organization, molecular crowding, and other nuclear-specific effects.

To assess the activity of gRNAs in a cellular context, we used a CRISPR activation system in which a denAsCas12a protein fused to a VPR transcriptional activator was directed to a GFP reporter gene. As gRNAs, we selected gRNA 14 and gRNA 7, which represented the least and most inhibited cases in our *in vitro* experiments, respectively, as well as gRNA 8 and gRNA 13, which showed intermediate inhibition in the presence of a random dsDNA background.

**Figure 5.**
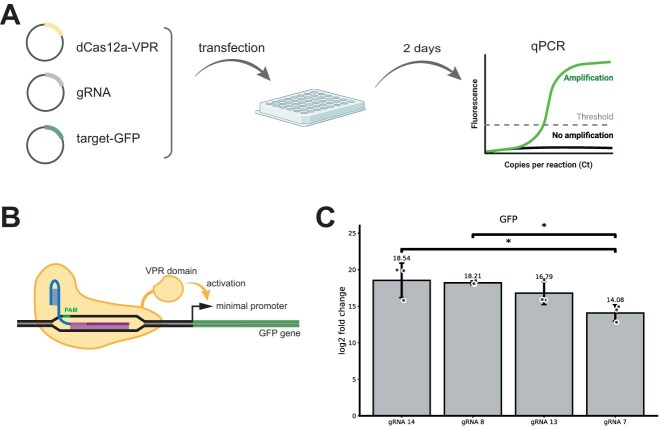
In cellulo experiment. (**A**) HEK293T cells are transfected with plasmids coding for denAsCas12a, a gRNA, and GFP under a minimal promoter activated by CRISPR activation upon binding of the Cas/gRNA complex to the target sequence in front of the promoter. Two days after transfection, total RNA is extracted and the GFP-mRNA levels are assessed by qPCR. (**B**) Scheme of the CRISPR activation assay producing GFP fluorescence signal as readout. The GFP gene is under control of a minimal promoter that is only active upon activation by the VP64-p65-Rta (VPR) domain. The VPR domain is linked to a catalytically inactive (dead) enAsCas12a protein. In front of the minimal promoter there are seven targets for the used gRNA (only one shown here) which bring the modified Cas protein close enough for activation upon binding. (**C**) Quantification of GFP expression levels using qPCR after 48 h. Bars show the average intensities for cells transfected with gRNA 14, gRNA 8, gRNA 13, and gRNA 7 gRNA/target constructs. Data are acquired from all RNA per well, shown by individual dots per well, originating from three biological replicates. Standard deviation of the mean is shown as error bar for each condition. gRNA 14 samples show higher fluorescence signal than the gRNA 7 samples. The difference between the conditions is significant with *P* = 0.0269231. Significance was assessed using one-way analysis of variance followed by Tukey’s honestly significant difference post hoc test. For a comparison with unregulated GFP expression see Supplementary Fig. S3.

To avoid kinetic effects introduced by protein expression or fluorescent protein maturation, we measured GFP messenger RNA (mRNA) levels directly using qPCR rather than monitoring GFP fluorescence (Fig. 5). The qPCR results follow the trend expected from the *in vitro* DETECTR experiments: higher *in vitro* kinetic ratios correspond to lower mRNA levels *in vivo*. The differences between the less inhibited guides (gRNAs 8, 13, and 14) are not statistically significant; however, the strongly inhibited gRNA 7 also shows the lowest mRNA level in the cellular experiments.

Importantly, in contrast to the *in vitro* experiments, we cannot directly compare gRNA activity in the presence or absence of a complex nucleic acid background. Consequently, we do not measure an *in vivo* equivalent of the kinetic ratio *r*, but instead compare the relative CRISPR activity of different gRNAs within the same cellular context. When comparing the *in vivo* trend with the half-times measured in the DETECTR experiments in the presence of background, we observe the same qualitative relationship (cf. Supplementary Fig. S4).

### Modeling of interactions with the dsDNA background

We can estimate the number of possible interaction sites for the Cas12a:gRNA within our 200 nM random dsDNA pool. Cas12a recognizes the PAM sequence TTTV, where V stands for any nucleotide except thymine (i.e. A, C, or G). The probability of finding this specific tetranucleotide in a random sequence of four nucleotides thus is: ${\mathrm{P}}( {{\mathrm{PAM}}} ) = {{( {\frac{1}{4}} )}^3} \cdot \frac{3}{4} = \frac{3}{{256}} \approx 0.0117$. In a DNA fragment of length *L* = 168 base pairs, the number of possible PAM positions is 2 ⋅ (L − 4 + 1) = 330. Therefore, we expect on average N(PAM) ≈ 3.86 PAMs on one representative of the random pool, corresponding to a total PAM concentration of 772 nM in the pool. In a similar way, we can calculate the average number of sequences that contain a PAM with a fitting seed region of length m nucleotides next to it, which gives: ${\mathrm{N}}( {{\mathrm{PAM\ }} + {\mathrm{\ m}}} ) = 2 \cdot ( {{\mathrm{L}} - {\mathrm{m}} + 1} ) \cdot {\mathrm{P}}( {{\mathrm{PAM}}} ) \cdot {{( {\frac{1}{4}} )}^{\mathrm{m}}}$ occurrences per DNA molecule. The number of occurrences exponentially drops with m—for example, the concentration of strands that contain a PAM and a neighboring 8 nt seed is only c(PAM + 8) ≈ 11 pM. The observed inhibitory effect is therefore kinetic in nature, as has previously been discussed for Cas9 target search using different experimental and theoretical approaches [14, 18–20, 38, 40]. Interestingly, the observed kinetics of our fluorescence assay can be accurately described by a simple model in which the entire random dsDNA pool is replaced by an effective “average” sequence. This average sequence is assumed to be present at the same total concentration as the pool and to interact with Cas12a:gRNA complexes for a characteristic residence time.

In the absence of a background, we can model the rise of the fluorescence signal based on the following reactions (Model 1):


\begin{eqnarray*}
{\mathrm{Cas}}12{\mathrm{a}}&:&{\mathrm{gRNA\ }} + {\mathrm{\ dsT\ }}\mathop \to \limits^{{{{\mathrm{k}}}_{{\mathrm{ON}},{\mathrm{T}}}}} {\mathrm{\ Cas}}12{\mathrm{a}}:{\mathrm{gRNA*}}\\{\mathrm{Cas}}12{\mathrm{a}}&:&{\mathrm{gRNA*\ }} + {\mathrm{\ Rep\ }}\mathop \to \limits^{{\mathrm{k}}2} {\mathrm{\ Cas}}12{\mathrm{a}}: {\mathrm{gRNA*\ }} + {\mathrm{\ Rep*}}.
\end{eqnarray*}

Here, dsT represents the cognate dsDNA target, Cas12-gRNA* stands for the activated effector complex, and Rep* is the fluorescent cleavage product of the doubly labeled reporter Rep. The fluorescence signal is assumed to be proportional to the concentration [Rep*]. Consistent with previous studies, we assume irreversible binding of the effector to its target [20, 40] and thus disregard the unbinding reaction. In the presence of a random pool, these processes are augmented by the interaction with the background dsB (Model 2):


\begin{eqnarray*}
{\mathrm{Cas}}12{\mathrm{a}}:{\mathrm{gRNA}} + {\mathrm{dsB}}\begin{array}{@{}*{1}{c}@{}} {{{{\mathrm{k}}}_{{\mathrm{ON}},{\mathrm{B}}}}}\\ \rightleftharpoons \\ {{{{\mathrm{k}}}_{{\mathrm{OFF}},{\mathrm{B}}}}} \end{array}{\mathrm{Cas}}12{\mathrm{a}}:{\mathrm{gRNA}} - {\mathrm{dsB}}.
\end{eqnarray*}

**Figure 6.**
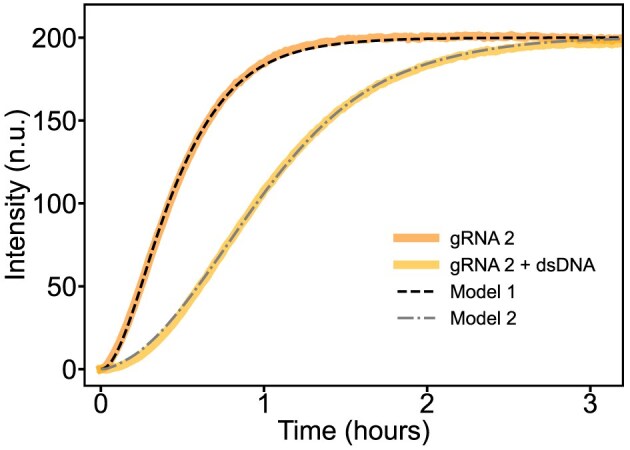
Simple kinetic model for the time course of the DETECTR reaction. Shown is the experimentally determined time-course for gRNA 2 and its cognate target in the absence and presence of a random DNA pool. Dashed and dash-dotted lines show the results of a simulation with the simple kinetic Model 1 and 2 as explained in the text.

As shown in Fig. 6, the models accurately reproduce the observed fluorescence time courses both in the absence and presence of the background pool. Notably, the extracted on-rates for the target and background strands differ by approximately two orders of magnitude (e.g ${{{\mathit{k}}}_{{\mathrm{ON,T}}}} = 1.8 \times {{10}^4}{{{\mathrm{M}}}^{ - 1}}{{{\mathrm{s}}}^{ - 1}}$, ${{{\mathit{k}}}_{{\mathrm{ON,B}}}} = 1.1 \times {{10}^6}{{{\mathrm{M}}}^{ - 1}}{{{\mathrm{s}}}^{ - 1}}$). It is important to note that ${{{\mathit{k}}}_{{\mathrm{ON,T}}}}$ represents a lumped rate constant, which encompasses not only initial binding, but also subsequent conformational changes and activation of the nuclease domain within the Cas12a:gRNA effector complex.

In the context of this model, the interaction of effector complexes with gRNAs of different sequence composition with the sequence background is reflected in different unbinding rates ${{{\mathit{k}}}_{{\mathrm{OFF,B}}}}$. In the example shown in Fig. 6, the off rate is ${{{\mathit{k}}}_{{\mathrm{OFF,B}}}} = 0.026{\mathrm{\ }}{{{\mathrm{s}}}^{ - 1}}$. This corresponds to an effective equilibrium dissociation constant of ${{{\mathit{K}}}_{{\mathrm{D}},{\mathrm{eff}}}} = {{{\mathit{k}}}_{{\mathrm{OFF}},{\mathrm{B}}}}/{{{\mathit{k}}}_{{\mathrm{ON}},{\mathrm{B}}}} = 24{\mathrm{\ nM}}$. This is similar to the *K*_D_ for a 10 bp long duplex.

## Discussion

### Main findings

The selection of target sequences and the design of gRNAs are critical steps in the implementation of CRISPR/Cas technologies, enabling diverse applications ranging from biosensing and biocomputing to gene and cell therapies. In cellular environments, Cas-gRNA complexes do not encounter isolated target DNA strands but must operate within a dense molecular milieu comprising numerous non-cognate DNA and RNA molecules—the genome and transcriptome, respectively. Similarly, in *in vitro* biosensing applications, specific target sequences must be reliably detected within the complex background of clinical samples. While significant efforts have been made in recent years to improve gRNA design to minimize off-target activity *in vivo* and reduce false positives in biosensing, less attention has been given to the kinetic variability that may arise from such off-target interactions.

In the present work, we systematically investigated how various factors influence the kinetics of Cas12a:gRNA target binding in complex sequence environments. Using a bottom-up *in vitro* approach, we first examined the kinetics of target search in the presence of designed background sequence pools, which allowed us to correlate the observed kinetic inhibition with specific design features, such as the presence of PAM motifs, seed region complementarity, or combinations thereof (Fig. 2). We next investigated the susceptibility of different gRNA sequence designs to interference from DNA and RNA strands with randomized sequences.

This set of experiments revealed a strong influence of both GC content and the purine-to-pyrimidine ratio within the target recognition site (spacer) of the gRNA (Figs 3 and 4). Across the 17 tested gRNAs with varying sequence features, the most favorable performance was observed for guides with a high AU:GC ratio combined with a high purine content. The overall trend can be captured by analytical expressions derived using symbolic regression. Notably, one of the tested guides (gRNA 7), which contains six uracils within the eight-nt seed region, exhibited exceptionally strong inhibition.

Initial experiments in human cells suggest that the kinetic effects observed *in vitro* may also influence CRISPR activity in a cellular context. Using qPCR of the corresponding mRNA, we monitored CRISPR activation of a GFP reporter gene in HEK293T cells using a catalytically inactive Cas12a variant. Although this variant does not cleave DNA, it still performs target search and is therefore subject to the same background interactions with endogenous RNA and genomic DNA. The expression levels induced by the four tested gRNAs followed the general trend expected from the *in vitro* inhibition experiments. Consistently, the use of gRNA 7 resulted in an exceptionally low mRNA expression level. A more systematic analysis will be required to fully understand these effects in cellular environments and in biosensing applications.

### Interpretation

To interpret our findings, we have to distinguish the different effects of RNA and DNA backgrounds as well as thermodynamic and kinetic effects:

*- RNA background*: Our results indicate that Cas12a activity is only weakly affected by the presence of an RNA sequence background, as a 200-fold excess relative to the target DNA concentration was required to observe a measurable effect. Two types of effects must be considered in this context. First, unbound gRNAs may hybridize with random RNA sequences to varying degrees, potentially forming inhibitory secondary structures that prevent loading into the Cas12a protein. Second, once the gRNA is incorporated into the Cas12a complex, it may still interact with surrounding RNA molecules. However, since Cas12a does not naturally target RNA, we consider such interactions to play only a minor role. Previous experiments with antisense RNAs for Cas9 gRNAs suggest that their presence reduces the availability of free gRNA for Cas9 loading [39, 40]. Thus, the primary effect of an RNA background is likely a reduction in the effective concentration of free gRNAs due to nonspecific sequestration. Among the different gRNAs tested, those with a high purine content were least affected by the RNA background, and the three-letter alphabet 3L-A (gRNA 3) sequence (only containing G,C,U) was most affected. This increased susceptibility potentially arises from the reduced sequence diversity combined with the high potential for both canonical (G–C, C–G, U–A) and wobble (G–U, U–G) base pairing, which enhances the probability of spurious interactions with background RNAs.

*- DNA background*: The effect of a dsDNA background is much more pronounced. Consistent with previous findings, the strongest interactions were observed when the Cas12a:gRNA complex encountered background sequences containing both a matching PAM and an adjacent seed region. In contrast, background sequences with only a PAM but no matching seed, or with a seed region but no PAM, did not lead to appreciable interactions.

- *Sequence dependence*: Why are gRNAs with high purine-to-pyrimidine ratios in the seed less affected by a dsDNA background? Because we quantified the kinetic ratio r (the slowdown caused by the random dsDNA pool), the observation could reflect (i) faster or more favorable binding/activation on the cognate target, (ii) reduced off-pathway interactions with non-cognate background DNA, or (iii) a combination of both. In hybrid strand-displacement experiments with RNA invading DNA duplexes, a high purine content in the invading RNA strand has been reported to accelerate displacement of the DNA [29], a similar effect may operate during Cas complex engagement with its DNA target. Moreover, higher A/U content and purine enrichment can reduce intra-gRNA secondary structure, potentially yielding a more accessible seed region that accelerates R-loop formation at the cognate site. Conversely, AU-rich seeds form less stable partial or mismatched heteroduplexes with random background DNA and thus dissociate more rapidly from non-cognate sites, while still nucleating efficiently at the true target where full complementarity stabilizes the complex.

Using symbolic regression, we derived analytical expressions that capture the trend in r values for the tested gRNAs as a function of purine fraction and either the uracil or cytosine content. The expressions obtained suggest that a high purine fraction in the seed region is generally favorable. Our most strongly affected gRNA, gRNA 7, has a large number of uracils in the seed, which may relate to the formation of hybrid wobble base pairs with background DNA. Remarkably, our fastest gRNA, gRNA 14 (*r* = 1.36), has a seed sequence very similar to that of gRNA 6, which nevertheless exhibits a kinetic ratio *r* of 3.

Overall, the sequence dependence thus appears to be complex. From a practical perspective, however, we believe that the derived expressions may serve as useful rules of thumb for gRNA design—bearing in mind that it does not capture the full complexity of the system.

- *In vivo relevance:* Our initial experiments suggest that designing guide sequences with a specific GC content and purine-to-pyrimidine ratio can be beneficial for target search and recognition, even *in vivo*. However, the effect is considerably less pronounced than under *in vitro* conditions. This is not surprising, given that the intracellular environment is far more complex due to factors such as compartmentalization, varying concentrations, and the different accessibility of binding partners. Within the nucleus, for example, the DNA concentration is on the order of micromolar, but only a fraction of the genomic DNA is accessible due to chromatin compaction. It has been noted that chromatin structure plays a crucial role in modulating target recognition by CRISPR–Cas effectors [41]. In addition, a potentially interfering RNA background is present in the nucleus, consisting of abundant mRNAs as well as various regulatory RNA species such as long non-coding RNAs, snoRNAs, and others, many of which localize to specific membraneless subcompartments. Such factors, along with those investigated in this study, interact in complex ways and must be considered collectively to achieve a holistic understanding of CRISPR–Cas target search and binding kinetics in cellular environments.

## Supplementary Material

gkag390_Supplemental_Files

## Data Availability

Python scripts for all figures, normalized data, and a list of sequences are available in the Zenodo repository under DOI: 10.5281/zenodo.19002498

## References

[1] Makarova KS, Wolf YI, Iranzo J et al. Evolutionary classification of CRISPR–Cas systems: a burst of class 2 and derived variants. Nat Rev Micro. 2020;18:67–83. 10.1038/s41579-019-0299-x PMC8905525 31857715

[2] Jinek M, Chylinski K, Fonfara I et al. A programmable Dual-RNA–guided DNA endonuclease in adaptive bacterial immunity. Science. 2012;337:816–21. 10.1126/science.1225829 22745249 PMC6286148

[3] Zetsche B, Gootenberg JS, Abudayyeh OO et al. Cpf1 is a single RNA-guided endonuclease of a class 2 CRISPR-Cas system. Cell. 2015;163:759–71. 10.1016/j.cell.2015.09.038 26422227 PMC4638220

[4] Cong L, Ran FA, Cox D et al. Multiplex genome engineering using CRISPR/Cas systems. Science. 2013;339:819–23. 10.1126/science.1231143 23287718 PMC3795411

[5] Chen JS, Ma E, Harrington LB et al. CRISPR-Cas12a target binding unleashes indiscriminate single-stranded DNase activity. Science. 2018;360:436–9. 10.1126/science.aar6245 29449511 PMC6628903

[6] Abudayyeh OO, Gootenberg JS, Konermann S et al. C2c2 is a single-component programmable RNA-guided RNA-targeting CRISPR effector. Science. 2016;353:aaf5573. 10.1126/science.aaf5573 27256883 PMC5127784

[7] Swarts DC, van der Oost J, Jinek M. Structural basis for guide RNA processing and seed-dependent DNA targeting by CRISPR-Cas12a. Mol Cell. 2017;66:221–33.e4. 10.1016/j.molcel.2017.03.016 28431230 PMC6879319

[8] Gootenberg JS, Abudayyeh OO, Kellner MJ. et al. Multiplexed and portable nucleic acid detection platform with Cas13, Cas12a, and Csm6. Science. 2018;360:439–44. 10.1126/science.aaq0179 29449508 PMC5961727

[9] Frangoul H, Locatelli F, Sharma A et al. Exagamglogene autotemcel for severe sickle cell disease. N Engl J Med. 2024;390:1649–62. 10.1056/NEJMoa2309676 38661449

[10] Slaymaker IM, Gao L, Zetsche B et al. Rationally engineered Cas9 nucleases with improved specificity. Science. 2016;351:84–8. 10.1126/science.aad5227 26628643 PMC4714946

[11] Chuai G, Ma H, Yan J et al. DeepCRISPR: optimized CRISPR guide RNA design by deep learning. Genome Biol. 2018;19:80. 10.1186/s13059-018-1459-4 29945655 PMC6020378

[12] Farasat I, Salis HM. A biophysical model of CRISPR/Cas9 activity for rational design of genome editing and gene regulation. Plos Comput Biol. 2016;12:e1004724. 10.1371/journal.pcbi.1004724 26824432 PMC4732943

[13] Jones DL, Leroy P, Unoson C et al. Kinetics of dCas9 target search in *Escherichia coli*. Science. 2017;357:1420–4. 10.1126/science.aah7084 28963258 PMC6150439

[14] Knight SC, Xie L, Deng W et al. Dynamics of CRISPR-Cas9 genome interrogation in living cells. Science. 2015;350:823–6. 10.1126/science.aac6572 26564855

[15] Sinan S, Appleby NM, Chou C-W et al. Kinetic dissection of pre-crRNA binding and processing by CRISPR–Cas12a. RNA. 2024;30:1345–55. 10.1261/rna.080088.124 39009379 PMC11404446

[16] Jeon Y, Choi YH, Jang Y et al. Direct observation of DNA target searching and cleavage by CRISPR-Cas12a. Nat Commun. 2018;9:2777. 10.1038/s41467-018-05245-x 30018371 PMC6050341

[17] Saha A, Ahsan M, Arantes PR et al. An alpha-helical lid guides the target DNA toward catalysis in CRISPR-Cas12a. Nat Commun. 2024;15:1473. 10.1038/s41467-024-45762-6 38368461 PMC10874386

[18] Strohkendl I, Saifuddin FA, Rybarski JR et al. Kinetic basis for DNA target specificity of CRISPR-Cas12a. Mol Cell. 2018;71:816–24.e3. 10.1016/j.molcel.2018.06.043 30078724 PMC6679935

[19] Klein M, Eslami-Mossallam B, Arroyo DG et al. Hybridization kinetics explains CRISPR-Cas off-targeting rules. Cell Rep. 2018;22:1413–23. 10.1016/j.celrep.2018.01.045 29425498

[20] Sternberg SH, Redding S, Jinek M et al. DNA interrogation by the CRISPR RNA-guided endonuclease Cas9. Nature. 2014;507:62–7. 10.1038/nature13011 24476820 PMC4106473

[21] Globyte V, Lee SH, Bae T et al. CRISPR/Cas9 searches for a protospacer adjacent motif by lateral diffusion. EMBO J. 2018;38:e99466. 10.15252/embj.201899466 30573670 PMC6376262

[22] Singh D, Sternberg SH, Fei J et al. Real-time observation of DNA recognition and rejection by the RNA-guided endonuclease Cas9. Nat Commun. 2016;7:12778. 10.1038/ncomms12778 27624851 PMC5027287

[23] Yang M, Sun R, Deng P et al. Nonspecific interactions between SpCas9 and dsDNA sites located downstream of the PAM mediate facilitated diffusion to accelerate target search. Chem Sci. 2021;12:12776–84. 10.1039/D1SC02633J 34703564 PMC8494019

[24] Sun R, Zhao Y, Wang W et al. Nonspecific interactions between Cas12a and dsDNA located downstream of the PAM mediate target search and assist AsCas12a for DNA cleavage. Chem Sci. 2023;14:3839–51. 10.1039/D2SC05463A 37035707 PMC10074435

[25] Szczelkun MD, Tikhomirova MS, Sinkunas T et al. Direct observation of R-loop formation by single RNA-guided Cas9 and cascade effector complexes. Proc Natl Acad Sci USA. 2014;111:9798–803. 10.1073/pnas.1402597111 24912165 PMC4103346

[26] Rutkauskas M, Songailiene I, Irmisch P et al. A quantitative model for the dynamics of target recognition and off-target rejection by the CRISPR-Cas cascade complex. Nat Commun. 2022;13:7460. 10.1038/s41467-022-35116-5 36460652 PMC9718816

[27] Mayer T, Oesinghaus L, Simmel FC. Toehold-mediated strand displacement in random sequence pools. J Am Chem Soc. 2023;145:634–44. 10.1021/jacs.2c11208 36571481

[28] Walbrun A, Wang T, Matthies M et al. Single-molecule force spectroscopy of toehold-mediated strand displacement. Nat Commun. 2024;15:7564. 10.1038/s41467-024-51813-9 39217165 PMC11365964

[29] Smith FG, Goertz JP, Jurinović K et al. Strong sequence–dependence in RNA/DNA hybrid strand displacement kinetics. Nanoscale. 2024;16:17624–37. 10.1039/D4NR00542B 39235130

[30] Fornace ME, Porubsky NJ, Pierce NA. A unified dynamic programming framework for the analysis of interacting nucleic acid strands: enhanced models, scalability, and speed. ACS Synth Biol. 2020;9:2665–78. 10.1021/acssynbio.9b00523 32910644

[31] Wolfe BR, Porubsky NJ, Zadeh JN et al. Constrained multistate sequence design for nucleic acid reaction pathway engineering. J Am Chem Soc. 2017;139:3134–44. 10.1021/jacs.6b12693 28191938

[32] Engler C, Kandzia R, Marillonnet S. A one pot, one step, precision cloning method with high throughput capability. PLoS One. 2008;3:e3647. 10.1371/journal.pone.0003647 18985154 PMC2574415

[33] Kim H, Lee W-j, Oh Y et al. Enhancement of target specificity of CRISPR–Cas12a by using a chimeric DNA–RNA guide. Nucleic Acids Res. 2020;48:8601–16. 10.1093/nar/gkaa605 32687187 PMC7470973

[34] Oesinghaus L, Simmel FC. Controlling gene expression in mammalian cells using multiplexed conditional guide RNAs for Cas12a**. Angew Chem Int Ed. 2021;60:23894–902. 10.1002/anie.202107258 PMC8596743 34533878

[35] Creutzburg SCA, Wu WY, Mohanraju P et al. Good guide, bad guide: spacer sequence-dependent cleavage efficiency of Cas12a. Nucleic Acids Res. 2020;48:3228–43. 10.1093/nar/gkz1240 31989168 PMC7102956

[36] Magnusson JP, Rios AR, Wu L et al. Enhanced Cas12a multi-gene regulation using a CRISPR array separator. eLife. 2021;10:e66406. 10.7554/eLife.66406 34499031 PMC8478413

[37] Kim HK, Song M, Lee J et al. *In vivo* high-throughput profiling of CRISPR–Cpf1 activity. Nat Methods. 2017;14:153–9. 10.1038/nmeth.4104 27992409

[38] Bisaria N, Jarmoskaite I, Herschlag D. Lessons from enzyme kinetics reveal specificity principles for RNA-guided nucleases in RNA interference and CRISPR-based genome editing. Cell Syst. 2017;4:21–9. 10.1016/j.cels.2016.12.010 28125791 PMC5308874

[39] Lee YJ, Hoynes-O’connor A, Leong MC et al. Programmable control of bacterial gene expression with the combined CRISPR and antisense RNA system. Nucleic Acids Res. 2016;44:2462–73. 10.1093/nar/gkw056 26837577 PMC4797300

[40] Mückl A, Schwarz-Schilling M, Fischer K et al. Filamentation and restoration of normal growth in *Escherichia coli* using a combined CRISPRi sgRNA/antisense RNA approach. PLoS One. 2018;13:e0198058. 30204770 10.1371/journal.pone.0198058 PMC6133276

[41] Strohkendl I, Saifuddin FA, Gibson BA et al. Inhibition of CRISPR-Cas12a DNA targeting by nucleosomes and chromatin. Sci Adv. 2021;7:eabd6030. 10.1126/sciadv.abd6030 33692102 PMC7946368

