# Supplementary material for: Operating CRISPR/Cas12a in a complex nucleic acid sequence background
Source: Nucleic Acids Res. 2026 Apr 30;54(8):gkag390. doi: 10.1093/nar/gkag390 (PMC13129545; doi:10.1093/nar/gkag390)
Supplement: gkag390_Supplemental_Files [file gkag390_supplemental_files.zip › Hellmer_NAR_SI.pdf]

SUPPORTING INFORMATION

Operating CRISPR/Cas12a in a complex nucleic acid sequence background

Henning Hellmer†, Thomas Mayer†, Lea Bauersachs†, Friedrich C. Simmel*

Department of Bioscience, School of Natural Sciences, Technical University of Munich, Garching, D-85748, Germany

† Henning Hellmer, Thomas Mayer, and Lea Bauersachs contributed equally to this work

* To whom correspondence should be addressed. Email: simmel@tum.de

Contents

1. Symbolic regression
2. qPCR experiments

Supplementary Figures S1-S4, Supplementary Tables 1-3

All other data, documentation and code relevant for the publication has been deposited in the Zenodo repository. DOI : 10.5281/zenodo.19002498

1. Symbolic Regression

1.1 Trends identified via symbolic regression

We performed symbolic regression on the dataset of 17 guide RNAs to identify compact mathematical expressions that capture the overall trends observed. We found that the behavior could already be captured when considering only the eight nucleotides in the guide recognition region. Furthermore, symbolic regression performed better when focusing on nucleotide fractions within this region, such as the purine fraction, the uracil fraction, the cytosine fraction, or the GC fraction.

Notably, these values are not independent: for example, the pyrimidine fraction is given by $f_Y = 1 - f_R$, and $f_P Y = f_C + f_U$. We found that the dataset could be fit well with high-complexity formulas, and as expected, the fit quality decreased when restricting the regression to lower-complexity expressions. In Fig. S1, we present example expressions obtained when using the purine and the uracil fraction as variables. In Fig. S1A, a very compact expression with a complexity of 8 captures the whole dataset with the exception of gRNA 7 (shown in orange). A more complex expression captures the behavior of gRNA 7 better (Fig. S1C), while the behavior of the entire dataset is best described by the most complex formula (Fig. S1E).

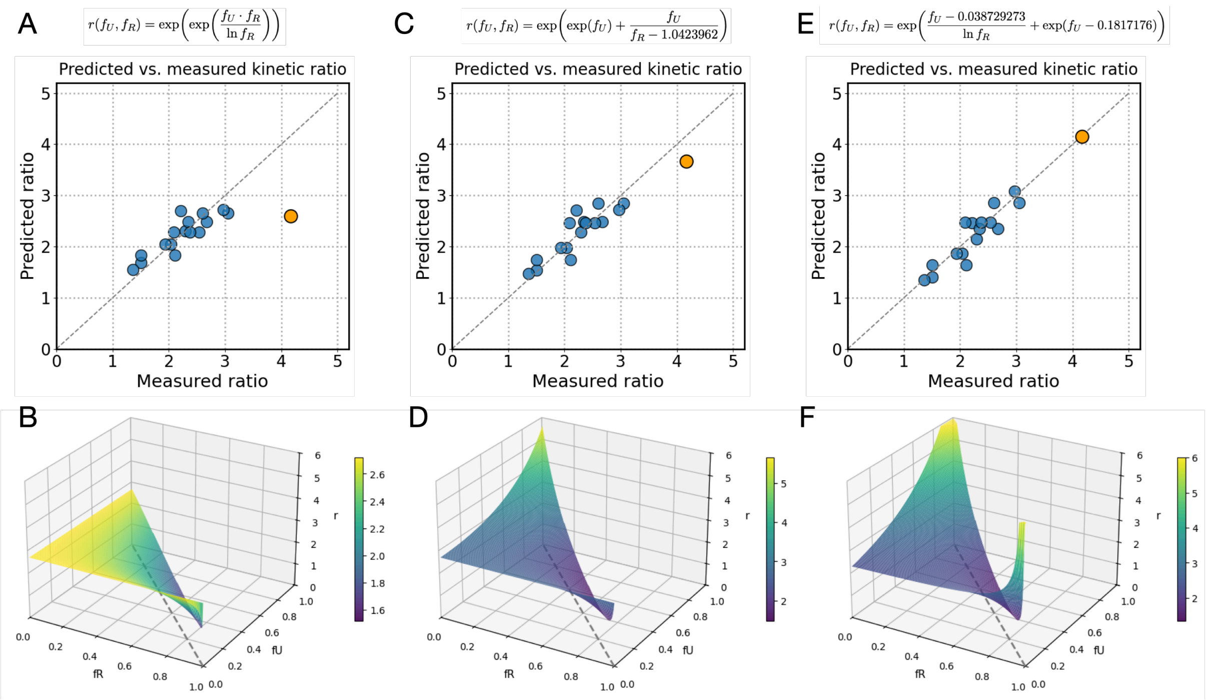

Supplementary Figure S1. Symbolic regression models for the kinetic slowdown ratio as a function of gRNA seed sequence composition. Each column shows one candidate expression selected from symbolic regression runs using purine fraction (fR) and uracil fraction (fU) as input features. The selected equation is shown above each column. **(A, C, E)** Predicted vs. measured kinetic slowdown ratio for all gRNAs (R^2 = 0.535, 0.850, 0.897 and RMSE = 0.445, 0.253, 0.210 for panels A, C, and E, respectively). The dashed line indicates perfect agreement. The orange symbol marks gRNA 7, an outlier in the (fR, fU) feature space. **(B, D, F)** Three-dimensional surface of the respective model function evaluated over the biologically accessible region of (fR, fU) parameter space (constraint: fU ≤ 1 − fR). Columns A/B, C/D, and E/F correspond to expressions of increasing complexity (8, 9, and 12, respectively).

We emphasize that these formulas are not intended as mechanistic models reflecting the physicochemical processes underlying the observed behavior. Rather, they should be regarded as compact representations of the dataset that facilitate discussion of trends in terms of nucleotide composition. We threfore plotted surface plots for the expressions in Fig. S1B,D, & F. These reveal the same overall behavior. From the surface plots we can infer that, in general, a higher purine fraction leads to a lower kinetic ratio r. The strong increase for high uracil fraction is relevant for gRNA 7, and is captured by the formulas in Fig. S1C & E.

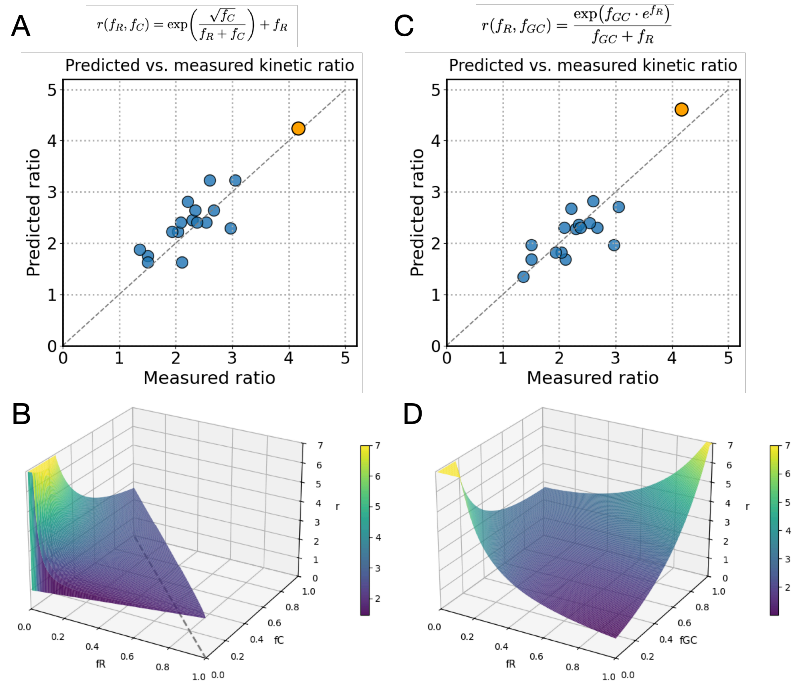

Supplementary Figure S2. Symbolic regression models for the kinetic slowdown ratio as a function of cytosine and GC content. The selected equations from two independent regression runs are shown above each column. (A, C) Predicted vs. measured kinetic slowdown ratio for all gRNAs (R^2 = 0.699 and RMSE = 0.358 for panel A; R^2 = 0.685 and RMSE = 0.366 for panel C). The dashed line indicates perfect agreement. The orange symbol marks gRNA 7. (B, D) Three-dimensional surface of the respective model function over the accessible parameter space. (A/B) Model using purine fraction and cytosine fraction (fR, fC) as input features; the surface is restricted to the biologically valid region fC ≤ 1 − fR. (C/D) Model using purine fraction and GC content (fR, fGC); since fGC combines one purine (G) and one pyrimidine (C), the full unit square is accessible.

For completeness, in Fig. S2 we also show example expressions as a function of the purine and cytosine fractions (Fig. S2A,B) and as a function of the purine fraction and the GC fraction (Fig. S2C,D). These expressions are likewise able to capture the observed trends in the dataset, but allow the trends to be rephrased in different compositional terms. For example, at low purine fraction the kinetic inhibition is strongest, and it becomes less pronounced with increasing cytosine or GC content. Notably, among the models of complexity 9 (Fig. S1 C, S2A, S2C) the one using purine and uracil content as parameters describes the data best.

1.2 Additional information for the symbolic regression procedure

Symbolic regression was performed using the PySR framework (v. 1.5.9) to identify analytical expressions relating gRNA sequence composition features to the measured kinetic slowdown ratio. Regression runs were conducted using two-feature input sets: purine fraction and uracil fraction (f_R, f_U), purine fraction and cytosine fraction (f_R, f_c), and purine fraction and GC content (f_R, f_{GC}), respectively. Each run used a population of 800 candidate expressions evolved over 1000 iterations with multithreaded parallelism across 6 processes. The operator set was restricted to binary operators {+, −, ×, ÷} and unary operators {exp, log, √}, with a maximum expression complexity of 12 and a maximum tree depth of 9. Mean squared error was used as the loss function. The resulting Pareto fronts (complexity vs. loss) were inspected and candidate equations were selected by balancing predictive accuracy against interpretability. In one run, gRNA 7 was excluded from the training set as an outlier in the (f_R, f_U), feature space. The code and underlying feature inputs are available in the zenodo repository.

2. Details on the qPCR experiments following the MIQE guidelines

2.1 qPCR results vs. half-times

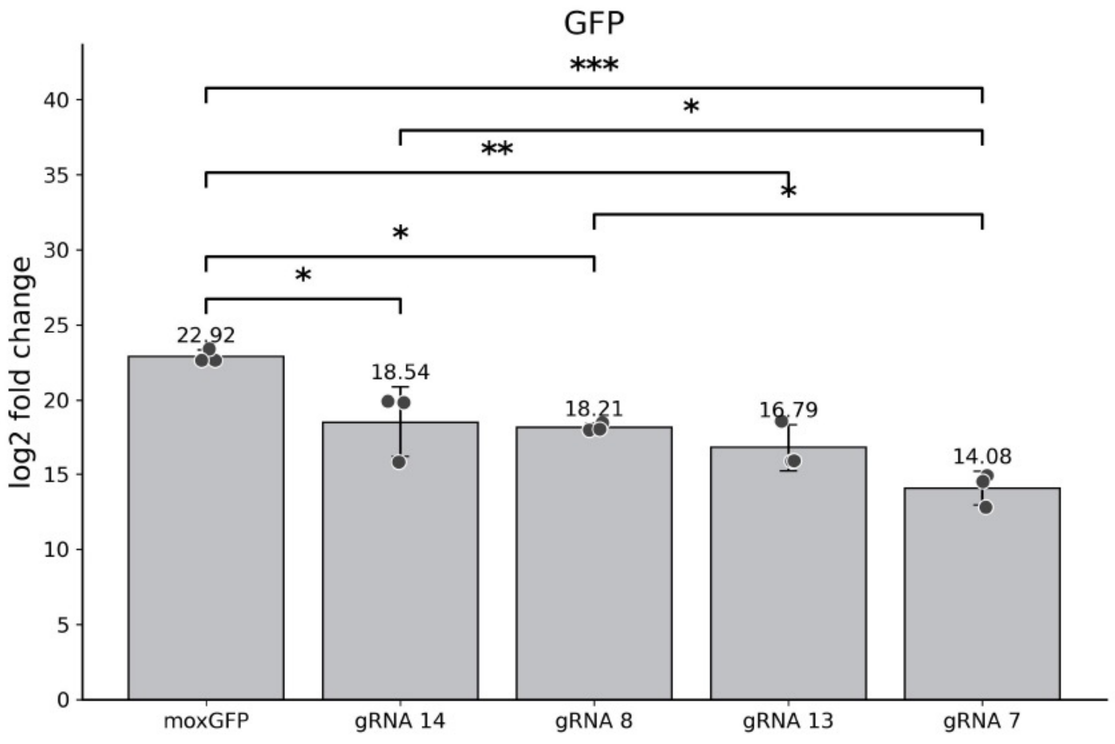

Supplementary Figure S3. qPCR analysis of GFP-mRNA. Shown is the $\log_2$ fold change in mRNA levels of unregulated moxGFP and GFP mRNA expressed via CRISPR activation using dCas9–VPR with different guide RNAs.

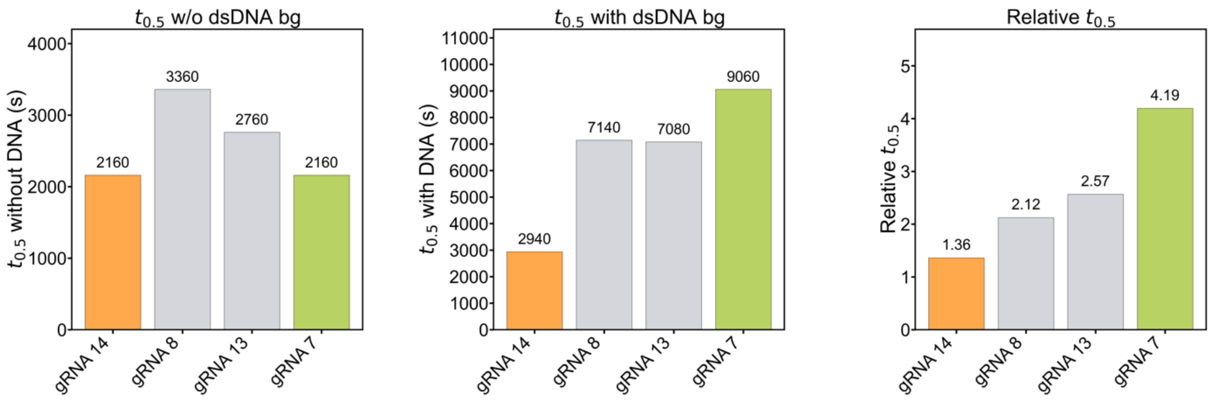

Supplementary Figure S4. Half-times and kinetic ratios. Shown are the DETECTR half-times of the gRNAs analyzed by qPCR in the absence and presence of a random dsDNA background (means of triplicates; cf. Figure 5), together with the corresponding kinetic ratios.

As shown in Fig. S3, the mRNA levels obtained for CRISPRa with gRNA 7 are the lowest, as determined by qPCR. This can be compared with the kinetics observed in our in vitro DETECTR experiments. As shown in Fig. S4, the in vivo situation more closely corresponds to the experiments performed in the presence of a dsDNA background, where gRNA 14 is the fastest and gRNA 7 the slowest. gRNA 8 and gRNA 13 exhibit similar kinetics in the in vitro experiments (with background), which is also consistent with the qPCR results. However, we do not observe a significantly higher expression level for mRNA controlled by gRNA 14 compared to gRNA 8 and gRNA 13.

2.2 Experimental details

The performance of CRISPR-mediated transcriptional activation using different guide RNAs (gRNAs) was evaluated in a cellular context using HEK293T cells cultured under standard conditions. Cells were seeded into 48-well plates at a density of 60,000 cells per well. Twenty-four hours after seeding, cells were transfected with three plasmids encoding the target construct, the gRNA, and the denCas12a–VPR activator. After an additional 24 hours, the culture medium was replaced.
Total RNA was isolated the following day using the Quick-RNA Miniprep Plus Kit (Zymo Research) according to the manufacturer's instructions, including the on-column DNase I digestion step. To ensure complete removal of residual genomic DNA, the eluted RNA was subjected to an additional DNase treatment using the TURBO DNA-free™ Kit (Invitrogen) under rigorous conditions. Briefly, 50 µL of undiluted RNA was transferred to a new tube, supplemented with 0.1 volumes of 10× TURBO DNase™ buffer and 2 µL of TURBO DNase™, and incubated at 37 °C for 1 h. Subsequently, 10 µL of DNase Inactivation Reagent was added, followed by incubation at room temperature for 5 min with gentle mixing. Samples were centrifuged at 10,000 × g for 1.5 min, and the supernatant was transferred to a fresh tube. RNA concentration was then determined.
RNA yield and purity were assessed using a NanoDrop absorbance spectrophotometer. All samples exhibited A260/280 ratios of around 2.0, indicating high purity. Isolated RNA was stored at −80 °C until further analysis.
cDNA synthesis was performed with 100 ng of RNA per sample using Maxima H Minus First Strand cDNA Synthesis Kit (ThermoFisher Scientific) according to the manufacturer's instructions for qPCR use.

qPCR was performed using the Luna® Universal qPCR Kit according to the manufacturer's protocol. Each condition was analyzed using biological triplicates with technical replicates for each sample, using 100 ng of cDNA per reaction. Reactions were carried out on a qTower^{3}G (Jena Analytics). Fluorescence was detected in the FAM channel with excitation at 470 nm and emission at 520 nm.

To confirm the absence of contaminating genomic DNA, control qPCR reactions lacking the reverse transcription step were performed using the Luna® Universal qPCR Master Mix according to the manufacturer's instructions in the qTower^{3}G as well. Each condition was analyzed using biological triplicates with technical replicates for each sample, using 100 ng of total RNA per reaction.

2.3 Data Analysis

The raw data was exported as a .csv file. The stability of the reference genes was analyzed using BestKeeper. Reference genes PPIA and 18S were found to be stable over all conditions. However, only PPIA was used because 18S rRNA has a huge dynamic range difference. Hence, small changes in mRNA become hard to normalize properly. The data was analyzed in the qPCRsoft4.1 software (Jena Analytics). Here, a quality check was performed, the curve inspection was performed, and the Ct values were calculated. For efficiency estimation, effciency-corrected ΔΔCt, reference gene validation and final fold change values, were calculated with the ΔΔCt method in the software. The statistical analysis was conducted with Python using a one-way ANOVA and Tukey's post-hoc test.

2.4 Primer Design and Validation

Primer specificity was verified in silico using NCBI Primer-BLAST against the RefSeq mRNA and human genome (GRCh38) databases for all primers.

- BLAST for 18S: The primer pair generated a single predicted amplicon (76 bp) specific for 18S rRNA (NR_003286), with multiple products detected in the human genome database. Since it is rRNA, exon-exon spanning was not applicable.

- BLAST for GAPDH: The primer pair generated a single predicted amplicon (159 bp) specific for GAPDH (NM_002046.7), with two additional products detected in the human genome database corresponding to the other GAPDH transcript variants as well. Primers specifically target transcript variant 1 (NM_002046.7). The forward primer spans the exon-exon junction 105/106 to prevent genomic DNA amplification. When searching for primer specificity in the whole genome, including genomic DNA, 25 unintended targets were found. Therefore, it is important to show that no genomic DNA is contained in the sample. However, because the primers span an exon junction, no genomic DNA should be amplified.

- BLAST for ACTB: The primer pair generated a single predicted amplicon (70 bp) specific for ACTB (NM_001101.5), with one additional products detected in the human genome database on chromosome 5 encoding for an ACTB isoform. The reverse primer spans the exon junction 78/79 to prevent genomic DNA amplification.

- BLAST for PPIA: The primer pair generated a single predicted amplicon (112 bp) specific for PPIA (NM_021130.5), with several additional products detected in the human genome database. Primers specifically target transcript variant 1 (NM_021130.5). The reverse primer spans the exon 1/2 junction to prevent genomic DNA amplification. Therefore, it is important to show that no genomic DNA is contained in the sample. However, because the primers span an exon junction, no genomic DNA should be amplified.

- BLAST for GFP: The primer pair generated a single predicted amplicon (188) specific for moxGFP, with no additional products detected in the human genome database. Because it is a synthetic protein, there is no exon-intron structure, hence the primers do not span any exon-exon junction.

Supplementary Table 1: Housekeeping Genes for RT-qPCR Normalization

Gene Symbol	Gene Name	Molecular Function	Accession Number
18S	18 S ribosomal RNA	Ribosomal RNA	NR_003286
ACTB	Beta-actin	Cytoskeletal structural protein	NM_001101
GAPDH	Glyceraldehyde-3-phosphate dehydrogenase	Glycolytic enzyme	NM_002046
PPIA	Peptidyl-prolyl cis-trans isomerase A	Cyclosporin binding protein	NM_021130

Supplementary Table 2: Primer Sequences

Primer Name	Primer Sequence
ACTB-F	CACAGAGCCTCGCCTTTGC
ACTB-R	ATATCATCATCCATGGTGAGCTGG
PPIA-F	GCCGAGGAAAACCGTGTACT
PPIA-R	CCTTGTCTGCAAACAGCTCA
GAPDH-F	AGGTCGGAGTCAACGGATTTGG
GAPDH-R	CATGGAATTTGCCATGGGTGGA
18S-F	TGGTTCCTTTGGTCGCTCG
18S-R	CGCCCGTCGGCATGTATTA
moxGFP-F	GACGACGGCACCTACAAGAC
MoxGFP-R	TTGGCCTTGATGCCGTTCTT

Supplementary Table 3: Primer Sequences for cDNA Synthesis

Primer Name	Primer Sequence
ACTB-F	AGATCATGTTTGAGACCTTCAACACCC
ACTB-R	AAGTCAGTGTACAGGTAAGCCCTG
PPIA-F	CGCCGAGGAAAACCGTGTA
PPIA-R	CAAAAATTAGCTGGGCATGGTGGC
GAPDH-F	GCTCCTCCTGTTCGACAGTC
GAPDH-R	TCTTACTCCTTGGAGGCCATGTG
18S-F	ATATGCTTGTCTCAAAGATTAAGCCATGCATG
18S-R	CCGTGTTGAGTCAAATTAAGCCGC
moxGFP-F	GTAAACGGCCACAAGTTCTCCG
MoxGFP-R	GCGGTCACGAATTCCAGAAGG

MoxGFP Sequence:

ATGGTGTCCAAGGGCGAGGAGCTGTTCACCGGGGTGGTGCCCATCCTGGTCGAGCTG
GACGGCGACGTAAACGGCCACAAGTTCTCCGTGCGGGGCGAGGGCGAGGGCGATGC
CACCAACGGCAAGCTGACCCTGAAGTTCATCAGCACCACCGGCAAGCTGCCCGTGCC
CTGGCCCACCCTCGTGACCACCCTGACCTACGGCGTGCAGAGCTTCTCCCGCTACCCC
GACCACATGAAGCGCCACGACTTCTTCAAGAGCGCCATGCCCGAAGGCTACGTCCAGG
AGCGCACCATCTCCTTCAAGGACGACGGCACCTACAAGACCCGCGCCGAGGTGAAGTT

CGAGGGCGACACCCTGGTGAACCGCATCGAGCTGAAGGGCATCGACTTCAAGGAGGA
CGGCAACATCCTGGGGCACAAGCTGGAGTACAACTTCAACTCCCACAACGTCTATATCA
CCGCCGACAAGCAGAAGAACGGCATCAAGGCCAACTTCAAGATCCGCCACAACGTGGA
GGACGGCTCCGTGCAGCTCGCCGACCACTACCAGCAGAACACCCCCATCGGCGACGG
CCCCGTGCTGCTGCCCGACAACCACTACCTGTCCACCCAGTCCAAGCTGTCCAAAGAC
CCCAACGAGAAGCGCGATCACATGGTCCTTCTGGAATTCGTGACCGCCGCCGGGATCA
CTCACGGCATGGACGAGCTGTACAAGTAA

2.5 Supporting data and images

Supporting data accompanying the qPCR experiments are attached to this document. In addition, we provide the qPCR report of our qPCR machine in the data repository for Fig. 6.
