# Supplementary material for: Operating CRISPR/Cas12a in a complex nucleic acid sequence background
Source: Nucleic Acids Res. 2026 Apr 30;54(8):gkag390. doi: 10.1093/nar/gkag390 (PMC13129545; doi:10.1093/nar/gkag390)
Supplement: gkag390_Supplemental_Files [file gkag390_supplemental_files.zip › qPCR-report.pdf]

Experiment

Title: TEMPLATE - qPCR

User: Administrator

Program

Preheat lid: ⊕

Lid temp. °C: 100

Step	Scan	Temp (°C)	Time (m:s)	Goto	Loops	+/- Temp (°C)	+/- Time (s)	Ramp (°C/s)
1		95,0	00:30	0	0	0,0	0	8,0
2		95,0	01:00	0	0	0,0	0	8,0
3		95,0	00:10	0	0	0,0	0	8,0
4		60,0	00:30	0	0	0,0	0	6,0
5	◆	70,0	00:03	3	44	0,0	0	6,0

Melting curve

Active: ⊕

Start temp. (°C)	End temp. (°C)	Increment (°C)	Equilibration (s)	Ramp (°C/s)
60,0	95,0	4,0	15	0,1

Scan

Meas. repeats: 3

Color compensation: ⊖

from column: 1

to column: 10

Pos.	Channel	Excitation	Detection	Dye	Gain	Measurement	Pass. Ref.
1	Blue	470	520	FAM	5	⊕	⊖
2	Green	515	545	JOE	5	⊖	⊖
3	Orange	565	605	ROX	5	⊖	⊖
4	Red	630	670	Cy5	5	⊖	⊖
5	Yellow	535	580	TAMRA	5	⊖	⊖

Control: Block Control

Start: 05.03.2026 19:50:52

End: 05.03.2026 21:16:47

Comment:

Settings

General

Title:	TEMPLATE - qPCR
Operator:	
Start:	05.03.2026 19:50:52
End:	05.03.2026 21:16:47
Comment:	

Settings

Thermal Cycler

Block type:	28
Lid temp.:	100°C
Hot start:	Yes
Control:	Block Control
Standby:	No
Block temp.:	12°C

Melting curve

Start temp.:	60°C
End temp.:	95°C
Gradient:	1°C
Ramp:	0,1°C/s
Equilibration:	15s
Active:	Yes

Program

Step	Scan	Temp (°C)	Time (m:s)	Goto	Loops	+/- Temp (°C)	+/- Time (s)	Ramp (°C/s)
1		95,0	00:30	0	0	0,0	0	8,0
1		95,0	00:30	0	0	0,0	0	8,0
1		95,0	00:30	0	0	0,0	0	8,0
1		95,0	00:30	0	0	0,0	0	8,0
1		95,0	00:30	0	0	0,0	0	8,0

Settings

Scan

Meas. repeats:	3
Color compensation:	No
from column:	1
to column:	10

Modules

Pos.	Channel	Excitation	Detection	Dye	Gain	Measurement	Pass. Ref.
1	Blue	470	520	FAM	5	⊕	⊖
2	Green	515	545	JOE	5	⊖	⊖
3	Orange	565	605	ROX	5	⊖	⊖
4	Red	630	670	Cy5	5	⊖	⊖
5	Yellow	535	580	TAMRA	5	⊖	⊖

Settings

Samples

	1	2	3	4	5	6	7	8	9	10	11	12
A	U FAM: GFP target7.3	U FAM: GFP fillup.3	U FAM: 18S pScaf.3	U FAM: PPIA target13.3	U FAM: PPIA untransfectec	U FAM: GFP target7.3	U FAM: GFP fillup.3	U FAM: 18S pScaf.3	U FAM: PPIA target13.3	U FAM: PPIA untransfectec	FAM:	FAM:
B	U FAM: GFP target8.3	U FAM: GFP reagent only.	U FAM: 18S BPK.3	U FAM: 18S target14.3	N FAM: GFP wasser	U FAM: GFP target8.3	U FAM: GFP reagent only.	U FAM: 18S BPK.3	U FAM: PPIA target14.3	FAM:	FAM:	FAM:
C	U FAM: GFP target13.3	U FAM: GFP untransfectec	U FAM: 18S pY010.3	U FAM: PPIA moxGFP.3	N FAM: GFP wasser	U FAM: GFP target13.3	U FAM: GFP untransfectec	U FAM: 18S pY010.3	U FAM: PPIA moxGFP.3	FAM:	FAM:	FAM:
D	U FAM: GFP target14.3	U FAM: 18S target7.3	U FAM: 18S fillup.3	U FAM: PPIA pScaf.3	N FAM: 18S wasser	U FAM: GFP target14.3	U FAM: 18S target7.3	U FAM: 18S fillup.3	U FAM: PPIA pScaf.3	FAM:	FAM:	FAM:
E	U FAM: GFP moxGFP.3	U FAM: 18S target8.3	U FAM: 18S reagent only.	U FAM: PPIA BPK.3	N FAM: 18S wasser	U FAM: GFP moxGFP.3	U FAM: 18S target8.3	U FAM: 18S reagent only.	U FAM: PPIA BPK.3	FAM:	FAM:	FAM:
F	U FAM: GFP pScaf.3	U FAM: 18S target13.3	U FAM: 18S untransfectec	U FAM: PPIA pY010.3	N FAM: PPIA wasser	U FAM: GFP pScaf.3	U FAM: 18S target13.3	U FAM: 18S untransfectec	U FAM: PPIA pY010.3	FAM:	FAM:	FAM:
G	U FAM: GFP BPK.3	U FAM: 18S target14.3	U FAM: PPIA target7.3	U FAM: PPIA fillup.3	N FAM: PPIA wasser	U FAM: GFP BPK.3	U FAM: 18S target14.3	U FAM: PPIA target7.3	U FAM: PPIA fillup.3	FAM:	FAM:	FAM:
H	U FAM: GFP pY010.3	U FAM: 18S moxGFP.3	U FAM: PPIA target8.3	U FAM: PPIA reagent only.3	K FAM: GFP calibrator	U FAM: GFP pY010.3	U FAM: 18S moxGFP.3	U FAM: PPIA target8.3	U FAM: PPIA reagent only.3	FAM:	FAM:	FAM:

Monitoring

Monitoring - RawData

All colors

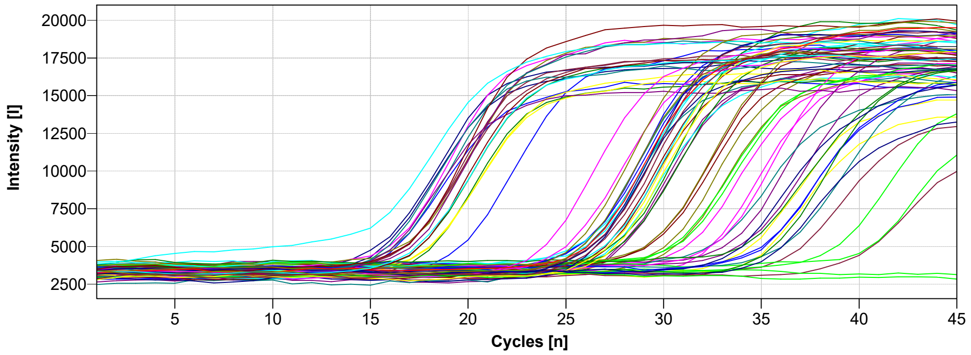

FAM

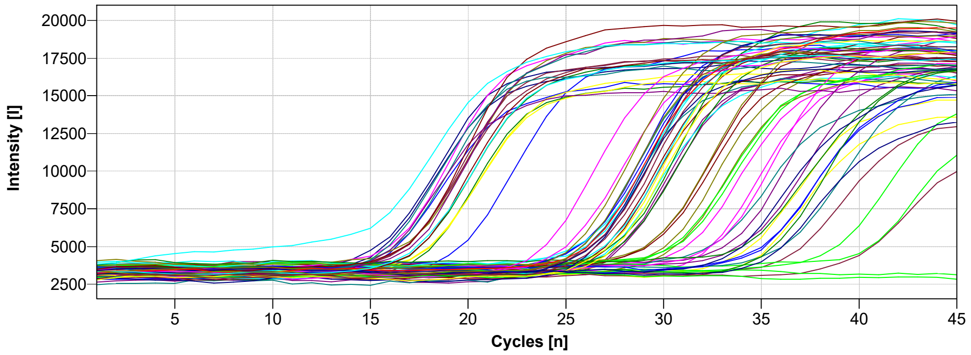

Monitoring

Monitoring - AmplifyData

All colors

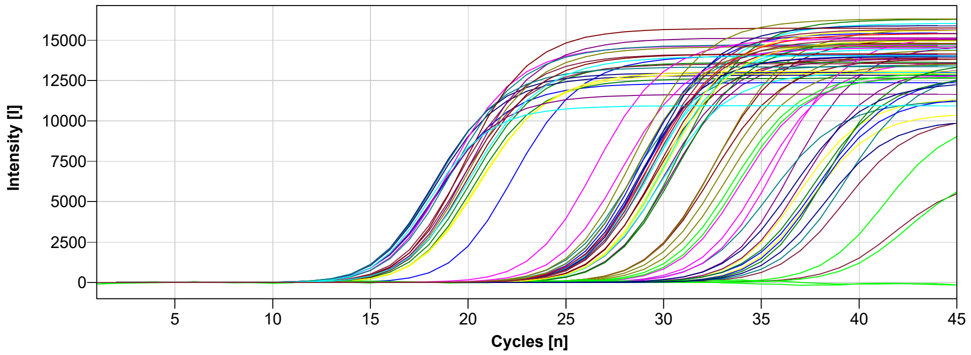

FAM

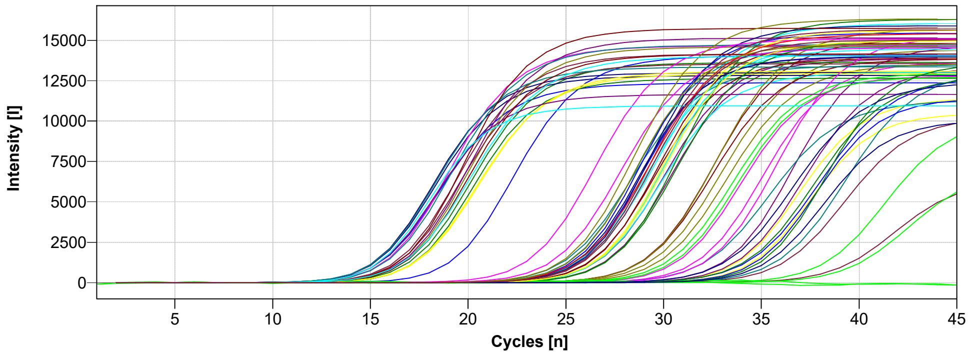

Monitoring

Monitoring - MeltingData

All colors

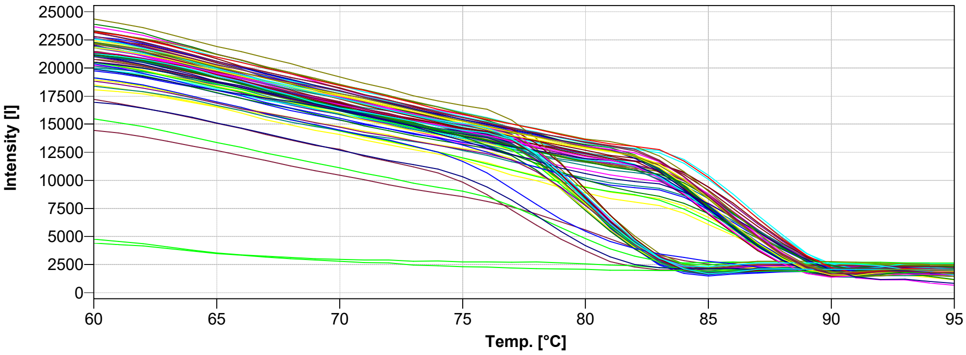

FAM

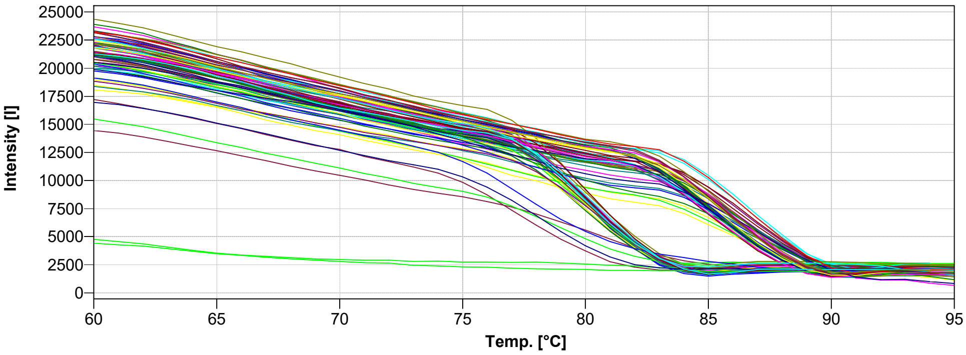

Monitoring

Ct

CT

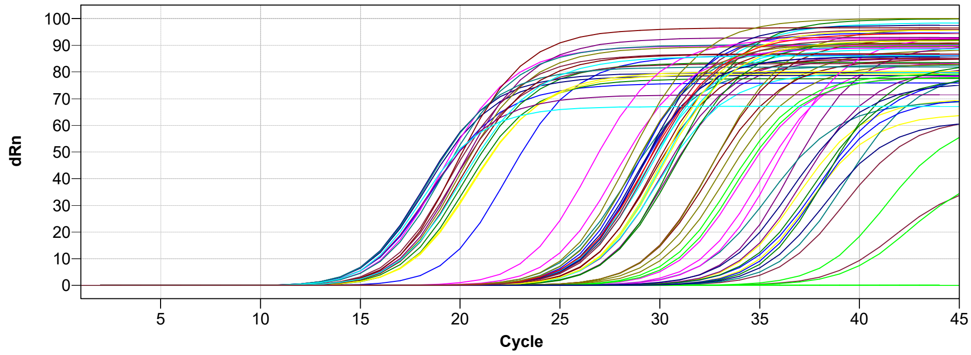

Well		Sample name	Sample type	Dye	Gene	Ct
A1		target7.3	U	FAM	GFP	28,61
A2		fillup.3	U	FAM	GFP	31,7
A4		target13.3	U	FAM	PPIA	25,02
A5		untransfected.3	U	FAM	PPIA	23,16
A6		target7.3	U	FAM	GFP	29,58
A7		fillup.3	U	FAM	GFP	32,54
A9		target13.3	U	FAM	PPIA	24,51
A10		untransfected.3	U	FAM	PPIA	22,79
B1		target8.3	U	FAM	GFP	27,15
B2		reagent only.3	U	FAM	GFP	34,06
B5		wasser	N	FAM	GFP	36,16
B6		target8.3	U	FAM	GFP	27,58
B7		reagent only.3	U	FAM	GFP	37,29
B9		target14.3	U	FAM	PPIA	24,18
C1		target13.3	U	FAM	GFP	31,03
C2		untransfected.3	U	FAM	GFP	29,97
C4		moxGFP.3	U	FAM	PPIA	24,57
C5		wasser	N	FAM	GFP	37,77
C6		target13.3	U	FAM	GFP	31,57
C7		untransfected.3	U	FAM	GFP	33,54
C9		moxGFP.3	U	FAM	PPIA	24,47
D1		target14.3	U	FAM	GFP	26,75
D4		pScaf.3	U	FAM	PPIA	23,33
D6		target14.3	U	FAM	GFP	26,76
D9		pScaf.3	U	FAM	PPIA	23,66
E1		moxGFP.3	U	FAM	GFP	23,52
E4		BPK.3	U	FAM	PPIA	25,09
E6		moxGFP.3	U	FAM	GFP	23,42
E9		BPK.3	U	FAM	PPIA	25,02
F1		pScaf.3	U	FAM	GFP	32,69
F4		pY010.3	U	FAM	PPIA	23,73
F5		wasser	N	FAM	PPIA	No Ct
F6		pScaf.3	U	FAM	GFP	32,03
F9		pY010.3	U	FAM	PPIA	23,51
G1		BPK.3	U	FAM	GFP	31,98
G3		target7.3	U	FAM	PPIA	21,01
G4		fillup.3	U	FAM	PPIA	24,42

Monitoring

Well		Sample name	Sample type	Dye	Gene	Ct
G5		wasser	N	FAM	PPIA	No Ct
G6		BPK.3	U	FAM	GFP	33,02
G8		target7.3	U	FAM	PPIA	22,2
G9		fillup.3	U	FAM	PPIA	24,45
H1		pY010.3	U	FAM	GFP	30,98
H3		target8.3	U	FAM	PPIA	22,76
H4		reagent only.3	U	FAM	PPIA	23,8
H5		calibrator	K	FAM	GFP	23,35
H6		pY010.3	U	FAM	GFP	33,19
H8		target8.3	U	FAM	PPIA	23,2
H9		reagent only.3	U	FAM	PPIA	23,9
A3		pScaf.3	U	FAM	18S	17,19
A8		pScaf.3	U	FAM	18S	13,64
B3		BPK.3	U	FAM	18S	15,14
B4		target14.3	U	FAM	18S	24,25
B8		BPK.3	U	FAM	18S	15,43
C3		pY010.3	U	FAM	18S	13,38
C8		pY010.3	U	FAM	18S	13,42
D2		target7.3	U	FAM	18S	13,95
D3		fillup.3	U	FAM	18S	15,34
D5		wasser	N	FAM	18S	28,1
D7		target7.3	U	FAM	18S	30,06
D8		fillup.3	U	FAM	18S	15,39
E2		target8.3	U	FAM	18S	14,9
E3		reagent only.3	U	FAM	18S	14,39
E5		wasser	N	FAM	18S	28,43
E7		target8.3	U	FAM	18S	26,78
E8		reagent only.3	U	FAM	18S	14,52
F2		target13.3	U	FAM	18S	14,76
F3		untransfected.3	U	FAM	18S	13,97
F7		target13.3	U	FAM	18S	13,67
F8		untransfected.3	U	FAM	18S	13,37
G2		target14.3	U	FAM	18S	14,65
G7		target14.3	U	FAM	18S	14,97
H2		moxGFP.3	U	FAM	18S	13,74
H7		moxGFP.3	U	FAM	18S	14,76

Well		Sample name	Mean Ct	Std.Dev. Ct
A1		target7.3	29,09	0,69
A2		fillup.3	32,12	0,59
A4		target13.3	24,77	0,36
A5		untransfected.3	22,97	0,26
A6		target7.3	29,09	0,69
A7		fillup.3	32,12	0,59
A9		target13.3	24,77	0,36
A10		untransfected.3	22,97	0,26
B1		target8.3	27,37	0,31
B2		reagent only.3	35,67	2,28
B5		wasser	36,97	1,14
B6		target8.3	27,37	0,31
B7		reagent only.3	35,67	2,28
B9		target14.3	24,18	0
C1		target13.3	31,3	0,38

Monitoring

Well		Sample name	Mean Ct	Std.Dev. Ct
C2		untransfected.3	31,76	2,53
C4		moxGFP.3	24,52	0,07
C5		wasser	36,97	1,14
C6		target13.3	31,3	0,38
C7		untransfected.3	31,76	2,53
C9		moxGFP.3	24,52	0,07
D1		target14.3	26,75	0
D4		pScaf.3	23,49	0,24
D6		target14.3	26,75	0
D9		pScaf.3	23,49	0,24
E1		moxGFP.3	23,47	0,07
E4		BPK.3	25,05	0,05
E6		moxGFP.3	23,47	0,07
E9		BPK.3	25,05	0,05
F1		pScaf.3	32,36	0,47
F4		pY010.3	23,62	0,15
F5		wasser		
F6		pScaf.3	32,36	0,47
F9		pY010.3	23,62	0,15
G1		BPK.3	32,5	0,73
G3		target7.3	21,6	0,84
G4		fillup.3	24,43	0,02
G5		wasser		
G6		BPK.3	32,5	0,73
G8		target7.3	21,6	0,84
G9		fillup.3	24,43	0,02
H1		pY010.3	32,09	1,56
H3		target8.3	22,98	0,31
H4		reagent only.3	23,85	0,07
H5		calibrator	23,35	0
H6		pY010.3	32,09	1,56
H8		target8.3	22,98	0,31
H9		reagent only.3	23,85	0,07
A3		pScaf.3	15,42	2,51
A8		pScaf.3	15,42	2,51
B3		BPK.3	15,29	0,2
B4		target14.3	17,95	5,46
B8		BPK.3	15,29	0,2
C3		pY010.3	13,4	0,03
C8		pY010.3	13,4	0,03
D2		target7.3	22	11,39
D3		fillup.3	15,36	0,03
D5		wasser	28,27	0,24
D7		target7.3	22	11,39
D8		fillup.3	15,36	0,03
E2		target8.3	20,84	8,4
E3		reagent only.3	14,46	0,09
E5		wasser	28,27	0,24
E7		target8.3	20,84	8,4
E8		reagent only.3	14,46	0,09
F2		target13.3	14,22	0,77
F3		untransfected.3	13,67	0,42
F7		target13.3	14,22	0,77

Monitoring

Well		Sample name	Mean Ct	Std.Dev. Ct
F8		untransfected.3	13,67	0,42
G2		target14.3	17,95	5,46
G7		target14.3	17,95	5,46
H2		moxGFP.3	14,25	0,72
H7		moxGFP.3	14,25	0,72

Monitoring

Tm

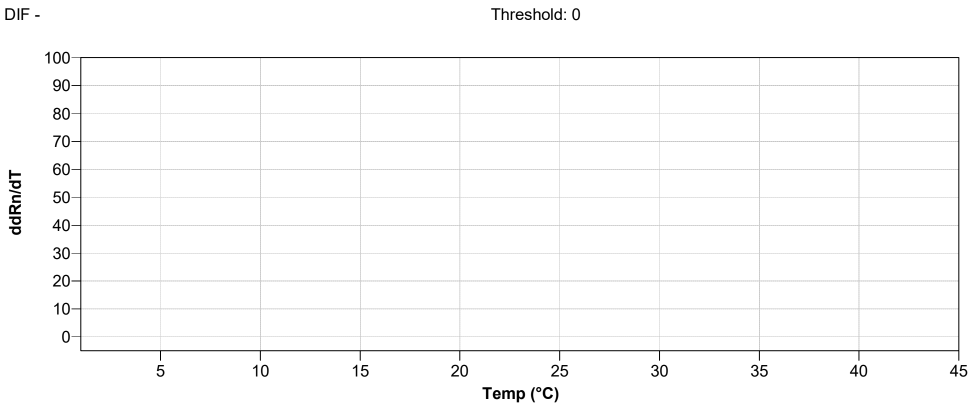

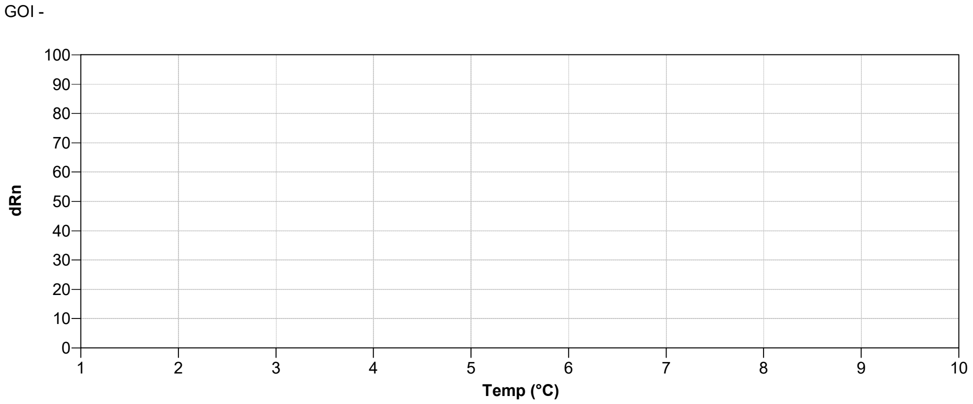

Well		Sample name	Sample type	Tm	Mean Tm	Std.Dev. Mean Tm
A1						
A2						
A3						
A4						
A5						
A6						
A7						
A8						
A9						
A10						
B1						
B2						
B3						
B4						
B5						
B6						
B7						
B8						
B9						
C1						
C2						
C3						
C4						
C5						

Monitoring

Well		Sample name	Sample type	Tm	Mean Tm	Std.Dev. Mean Tm
C6						
C7						
C8						
C9						
D1						
D2						
D3						
D4						
D5						
D6						
D7						
D8						
D9						
E1						
E2						
E3						
E4						
E5						
E6						
E7						
E8						
E9						
F1						
F2						
F3						
F4						
F5						
F6						
F7						
F8						
F9						
G1						
G2						
G3						
G4						
G5						
G6						
G7						
G8						
G9						
H1						
H2						
H3						
H4						
H5						
H6						
H7						
H8						
H9						

ddCt quantification

GOI - FAM Threshold: 3,08

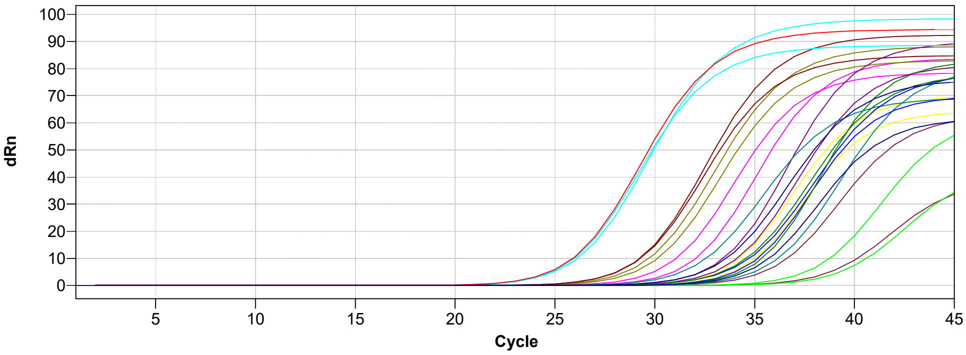

Ref.gene - FAM Threshold: 2,07

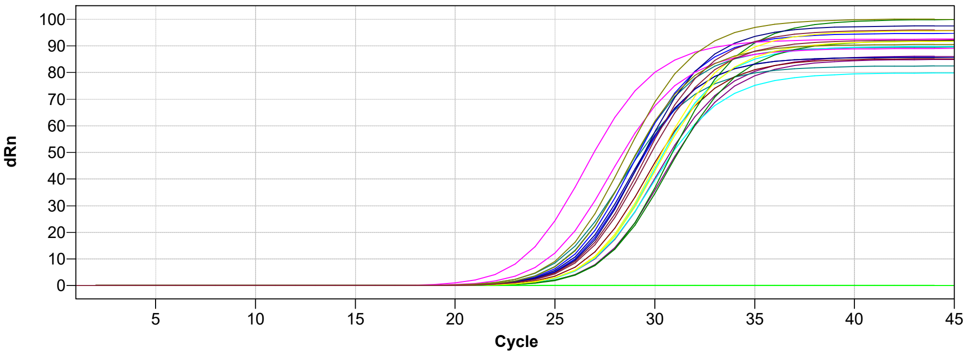

BAR

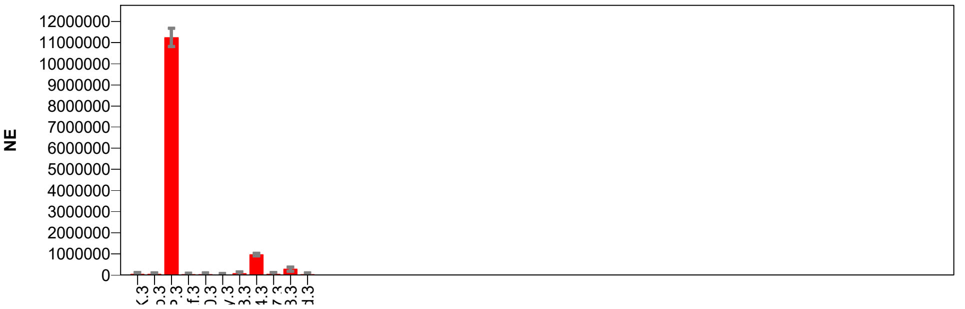

ddCt quantification

ddCt quantification

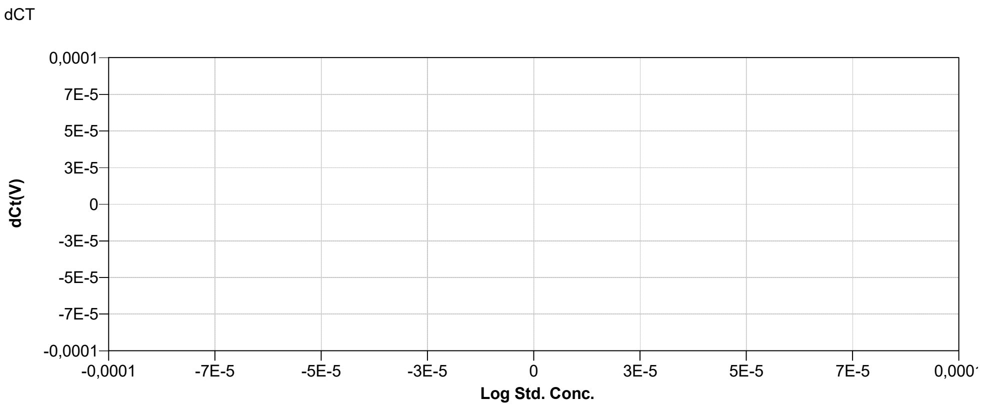

Well		Sample name	Sample type	GOI	Reference gene	Ct GOI
A1		target7.3	U	GFP		29,2
A2		fillup.3	U	GFP		32,28
A3		pScaf.3	U			
A4		target13.3	U		PPIA	
A5		untransfected.3	U		PPIA	
A6		target7.3	U	GFP		30,18
A7		fillup.3	U	GFP		33,14
A8		pScaf.3	U			
A9		target13.3	U		PPIA	
A10		untransfected.3	U		PPIA	
B1		target8.3	U	GFP		27,74
B2		reagent only.3	U	GFP		34,62
B3		BPK.3	U			
B4		target14.3	U			
B5		wasser	N	GFP		36,77
B6		target8.3	U	GFP		28,2
B7		reagent only.3	U	GFP		37,99
B8		BPK.3	U			
B9		target14.3	U		PPIA	
C1		target13.3	U	GFP		31,54
C2		untransfected.3	U	GFP		30,52
C3		pY010.3	U			
C4		moxGFP.3	U		PPIA	
C5		wasser	N	GFP		38,4
C6		target13.3	U	GFP		32,19
C7		untransfected.3	U	GFP		34,18
C8		pY010.3	U			
C9		moxGFP.3	U		PPIA	
D1		target14.3	U	GFP		27,32
D2		target7.3	U			
D3		fillup.3	U			
D4		pScaf.3	U		PPIA	
D5		wasser	N			
D6		target14.3	U	GFP		27,33
D7		target7.3	U			
D8		fillup.3	U			
D9		pScaf.3	U		PPIA	

ddCt quantification

Well		Sample name	Sample type	GOI	Reference gene	Ct GOI
E1		moxGFP.3	U	GFP		24,16
E2		target8.3	U			
E3		reagent only.3	U			
E4		BPK.3	U		PPIA	
E5		wasser	N			
E6		moxGFP.3	U	GFP		24,06
E7		target8.3	U			
E8		reagent only.3	U			
E9		BPK.3	U		PPIA	
F1		pScaf.3	U	GFP		33,26
F2		target13.3	U			
F3		untransfected.3	U			
F4		pY010.3	U		PPIA	
F5		wasser	N		PPIA	
F6		pScaf.3	U	GFP		32,62
F7		target13.3	U			
F8		untransfected.3	U			
F9		pY010.3	U		PPIA	
G1		BPK.3	U	GFP		32,54
G2		target14.3	U			
G3		target7.3	U		PPIA	
G4		fillup.3	U		PPIA	
G5		wasser	N		PPIA	
G6		BPK.3	U	GFP		33,51
G7		target14.3	U			
G8		target7.3	U		PPIA	
G9		fillup.3	U		PPIA	
H1		pY010.3	U	GFP		31,54
H2		moxGFP.3	U			
H3		target8.3	U		PPIA	
H4		reagent only.3	U		PPIA	
H5		calibrator	K	GFP		24,01
H6		pY010.3	U	GFP		33,79
H7		moxGFP.3	U			
H8		target8.3	U		PPIA	
H9		reagent only.3	U		PPIA	

Well		Sample name	Ct Ref. gene	Mean Ct GOI	Mean Ct Ref.gene	RQ GOI
A1		target7.3		29,69		0,02
A2		fillup.3		32,71		0
A3		pScaf.3				
A4		target13.3	25,02		24,77	
A5		untransfected.3	23,16		22,97	
A6		target7.3		29,69		0,02
A7		fillup.3		32,71		0
A8		pScaf.3				
A9		target13.3	24,51		24,77	
A10		untransfected.3	22,79		22,97	
B1		target8.3		27,97		0,06
B2		reagent only.3		36,3		0
B3		BPK.3				
B4		target14.3				
B5		wasser		37,58		0

ddCt quantification

Well		Sample name	Ct Ref. gene	Mean Ct GOI	Mean Ct Ref.gene	RQ GOI
B6		target8.3		27,97		0,06
B7		reagent only.3		36,3		0
B8		BPK.3				
B9		target14.3	24,18		24,18	
C1		target13.3		31,87		0
C2		untransfected.3		32,35		0
C3		pY010.3				
C4		moxGFP.3	24,57		24,52	
C5		wasser		37,58		0
C6		target13.3		31,87		0
C7		untransfected.3		32,35		0
C8		pY010.3				
C9		moxGFP.3	24,47		24,52	
D1		target14.3		27,33		0,1
D2		target7.3				
D3		fillup.3				
D4		pScaf.3	23,33		23,49	
D5		wasser				
D6		target14.3		27,33		0,1
D7		target7.3				
D8		fillup.3				
D9		pScaf.3	23,66		23,49	
E1		moxGFP.3		24,11		0,93
E2		target8.3				
E3		reagent only.3				
E4		BPK.3	25,09		25,05	
E5		wasser				
E6		moxGFP.3		24,11		0,93
E7		target8.3				
E8		reagent only.3				
E9		BPK.3	25,02		25,05	
F1		pScaf.3		32,94		0
F2		target13.3				
F3		untransfected.3				
F4		pY010.3	23,73		23,62	
F5		wasser	No Ct			
F6		pScaf.3		32,94		0
F7		target13.3				
F8		untransfected.3				
F9		pY010.3	23,51		23,62	
G1		BPK.3		33,03		0
G2		target14.3				
G3		target7.3	21,01		21,6	
G4		fillup.3	24,42		24,43	
G5		wasser	No Ct			
G6		BPK.3		33,03		0
G7		target14.3				
G8		target7.3	22,2		21,6	
G9		fillup.3	24,45		24,43	
H1		pY010.3		32,66		0
H2		moxGFP.3				
H3		target8.3	22,76		22,98	
H4		reagent only.3	23,8		23,85	

ddCt quantification

Well		Sample name	Ct Ref. gene	Mean Ct GOI	Mean Ct Ref.gene	RQ GOI
H5		calibrator		24,01		1
H6		pY010.3		32,66		0
H7		moxGFP.3				
H8		target8.3	23,2		22,98	
H9		reagent only.3	23,9		23,85	

Well		Sample name	RQ Ref.gene	Norm. Expression	Std.Dev. RQ GOI	Std.Dev. RQ Ref.gene
A1		target7.3		31037,2014	0,01	
A2		fillup.3		27171,866	0	
A3		pScaf.3				
A4		target13.3	0	61482,8755		0
A5		untransfected.3	0	12654,5004		0
A6		target7.3		31037,2014	0,01	
A7		fillup.3		27171,866	0	
A8		pScaf.3				
A9		target13.3	0	61482,8755		0
A10		untransfected.3	0	12654,5004		0
B1		target8.3		265618,4841	0,01	
B2		reagent only.3		1502,8228	0	
B3		BPK.3				
B4		target14.3				
B5		wasser			0	
B6		target8.3		265618,4841	0,01	
B7		reagent only.3		1502,8228	0	
B8		BPK.3				
B9		target14.3	0	953982,2794		0
C1		target13.3		61482,8755	0	
C2		untransfected.3		12654,5004	0,01	
C3		pY010.3				
C4		moxGFP.3	0	11224285,0045		0
C5		wasser			0	
C6		target13.3		61482,8755	0	
C7		untransfected.3		12654,5004	0,01	
C8		pY010.3				
C9		moxGFP.3	0	11224285,0045		0
D1		target14.3		953982,2794	0	
D2		target7.3				
D3		fillup.3				
D4		pScaf.3	0	12072,3352		0
D5		wasser				
D6		target14.3		953982,2794	0	
D7		target7.3				
D8		fillup.3				
D9		pScaf.3	0	12072,3352		0
E1		moxGFP.3		11224285,0045	0,04	
E2		target8.3				
E3		reagent only.3				
E4		BPK.3	0	33568,6779		0
E5		wasser				
E6		moxGFP.3		11224285,0045	0,04	
E7		target8.3				
E8		reagent only.3				
E9		BPK.3	0	33568,6779		0

ddCt quantification

Well		Sample name	RQ Ref.gene	Norm. Expression	Std.Dev. RQ GOI	Std.Dev. RQ Ref.gene
F1		pScaf.3		12072,3352	0	
F2		target13.3				
F3		untransfected.3				
F4		pY010.3	0	15943,8469		0
F5		wasser				
F6		pScaf.3		12072,3352	0	
F7		target13.3				
F8		untransfected.3				
F9		pY010.3	0	15943,8469		0
G1		BPK.3		33568,6779	0	
G2		target14.3				
G3		target7.3	0	31037,2014		0
G4		fillup.3	0	27171,866		0
G5		wasser				
G6		BPK.3		33568,6779	0	
G7		target14.3				
G8		target7.3	0	31037,2014		0
G9		fillup.3	0	27171,866		0
H1		pY010.3		15943,8469	0	
H2		moxGFP.3				
H3		target8.3	0	265618,4841		0
H4		reagent only.3	0	1502,8228		0
H5		calibrator			0	
H6		pY010.3		15943,8469	0	
H7		moxGFP.3				
H8		target8.3	0	265618,4841		0
H9		reagent only.3	0	1502,8228		0

Well		Sample name	Std.Dev. Norm. Expression	dCt (Ref.Gen – GOI)
A1		target7.3	23364,4792	-8,09
A2		fillup.3	11507,7885	-8,28
A3		pScaf.3		
A4		target13.3		
A5		untransfected.3		
A6		target7.3	23364,4792	-8,09
A7		fillup.3	11507,7885	-8,28
A8		pScaf.3		
A9		target13.3		
A10		untransfected.3		
B1		target8.3	82548,4445	-4,99
B2		reagent only.3	2480,4019	-12,45
B3		BPK.3		
B4		target14.3		
B5		wasser		
B6		target8.3	82548,4445	-4,99
B7		reagent only.3	2480,4019	-12,45
B8		BPK.3		
B9		target14.3		
C1		target13.3	24782,9976	-7,1
C2		untransfected.3	22805,6708	-9,38
C3		pY010.3		
C4		moxGFP.3		
C5		wasser		

ddCt quantification

Well		Sample name	Std.Dev. Norm. Expression	dCt (Ref.Gen – GOI)
C6		target13.3	24782,9976	-7,1
C7		untransfected.3	22805,6708	-9,38
C8		pY010.3		
C9		moxGFP.3		
D1		target14.3	5595,6298	-3,14
D2		target7.3		
D3		fillup.3		
D4		pScaf.3		
D5		wasser		
D6		target14.3	5595,6298	-3,14
D7		target7.3		
D8		fillup.3		
D9		pScaf.3		
E1		moxGFP.3	741670,2681	0,41
E2		target8.3		
E3		reagent only.3		
E4		BPK.3		
E5		wasser		
E6		moxGFP.3	741670,2681	0,41
E7		target8.3		
E8		reagent only.3		
E9		BPK.3		
F1		pScaf.3	4263,9239	-9,45
F2		target13.3		
F3		untransfected.3		
F4		pY010.3		
F5		wasser		
F6		pScaf.3	4263,9239	-9,45
F7		target13.3		
F8		untransfected.3		
F9		pY010.3		
G1		BPK.3	16040,8359	-7,97
G2		target14.3		
G3		target7.3		
G4		fillup.3		
G5		wasser		
G6		BPK.3	16040,8359	-7,97
G7		target14.3		
G8		target7.3		
G9		fillup.3		
H1		pY010.3	17678,5839	-9,05
H2		moxGFP.3		
H3		target8.3		
H4		reagent only.3		
H5		calibrator		
H6		pY010.3	17678,5839	-9,05
H7		moxGFP.3		
H8		target8.3		
H9		reagent only.3		

Experiment

Title: TEMPLATE - qPCR

User: Administrator

Program

Preheat lid: ⊕

Lid temp. °C: 100

Step	Scan	Temp (°C)	Time (m:s)	Goto	Loops	+/- Temp (°C)	+/- Time (s)	Ramp (°C/s)
1		95,0	00:30	0	0	0,0	0	8,0
2		95,0	01:00	0	0	0,0	0	8,0
3		95,0	00:10	0	0	0,0	0	8,0
4		60,0	00:30	0	0	0,0	0	6,0
5	◆	70,0	00:03	3	44	0,0	0	6,0

Melting curve

Active: ⊕

Start temp. (°C)	End temp. (°C)	Increment (°C)	Equilibration (s)	Ramp (°C/s)
60,0	95,0	4,0	15	0,1

Scan

Meas. repeats: 3

Color compensation: ⊖

from column: 1

to column: 10

Pos.	Channel	Excitation	Detection	Dye	Gain	Measurement	Pass. Ref.
1	Blue	470	520	FAM	5	⊕	⊖
2	Green	515	545	JOE	5	⊖	⊖
3	Orange	565	605	ROX	5	⊖	⊖
4	Red	630	670	Cy5	5	⊖	⊖
5	Yellow	535	580	TAMRA	5	⊖	⊖

Control: Block Control

Start: 05.03.2026 16:53:56

End: 05.03.2026 18:20:44

Comment:

Settings

General

Title:	TEMPLATE - qPCR
Operator:	
Start:	05.03.2026 16:53:56
End:	05.03.2026 18:20:44
Comment:	

Settings

Thermal Cycler

Block type:	28
Lid temp.:	100°C
Hot start:	Yes
Control:	Block Control
Standby:	No
Block temp.:	12°C

Melting curve

Start temp.:	60°C
End temp.:	95°C
Gradient:	1°C
Ramp:	0,1°C/s
Equilibration:	15s
Active:	Yes

Program

Step	Scan	Temp (°C)	Time (m:s)	Goto	Loops	+/- Temp (°C)	+/- Time (s)	Ramp (°C/s)
1		95,0	00:30	0	0	0,0	0	8,0
1		95,0	00:30	0	0	0,0	0	8,0
1		95,0	00:30	0	0	0,0	0	8,0
1		95,0	00:30	0	0	0,0	0	8,0
1		95,0	00:30	0	0	0,0	0	8,0

Settings

Scan

Meas. repeats:	3
Color compensation:	No
from column:	1
to column:	10

Modules

Pos.	Channel	Excitation	Detection	Dye	Gain	Measurement	Pass. Ref.
1	Blue	470	520	FAM	5	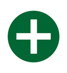	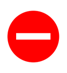
2	Green	515	545	JOE	5	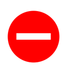	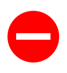
3	Orange	565	605	ROX	5	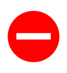	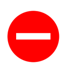
4	Red	630	670	Cy5	5	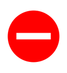	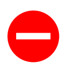
5	Yellow	535	580	TAMRA	5	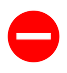	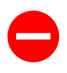

Settings

Samples

	1	2	3	4	5	6	7	8	9	10	11	12
A	U FAM: GFP target7.1	U FAM: GFP fillup.1	U FAM: 18S pScaf.1	U FAM: PPIA target13.1	U FAM: PPIA untransfected	U FAM: GFP target7.1	U FAM: GFP fillup.1	U FAM: 18S pScaf.1	U FAM: PPIA target13.1	U FAM: PPIA untransfected	FAM:	FAM:
B	U FAM: GFP target8.1	U FAM: GFP reagent only.1	U FAM: 18S BPK.1	U FAM: 18S target14.1	N FAM: GFP wasser	U FAM: GFP target8.1	U FAM: GFP reagent only.1	U FAM: 18S BPK.1	U FAM: PPIA target14.1	FAM:	FAM:	FAM:
C	U FAM: GFP target13.1	U FAM: GFP untransfected	U FAM: 18S pY010.1	U FAM: PPIA moxGFP.1	N FAM: GFP wasser	U FAM: GFP target13.1	U FAM: GFP untransfected	U FAM: 18S pY010.1	U FAM: PPIA moxGFP.1	FAM:	FAM:	FAM:
D	U FAM: GFP target14.1	U FAM: 18S target7.1	U FAM: 18S fillup.1	U FAM: PPIA pScaf.1	N FAM: 18S wasser	U FAM: GFP target14.1	U FAM: 18S target7.1	U FAM: 18S fillup.1	U FAM: PPIA pScaf.1	FAM:	FAM:	FAM:
E	U FAM: GFP moxGFP.1	U FAM: 18S target8.1	U FAM: 18S reagent only.1	U FAM: PPIA BPK.1	N FAM: 18S wasser	U FAM: GFP moxGFP.1	U FAM: 18S target8.1	U FAM: 18S reagent only.1	U FAM: PPIA BPK.1	FAM:	FAM:	FAM:
F	U FAM: GFP pScaf.1	U FAM: 18S target13.1	U FAM: 18S untransfected	U FAM: PPIA pY010.1	N FAM: PPIA wasser	U FAM: GFP pScaf.1	U FAM: 18S target13.1	U FAM: 18S untransfected	U FAM: PPIA pY010.1	FAM:	FAM:	FAM:
G	U FAM: GFP BPK.1	U FAM: 18S target14.1	U FAM: PPIA target7.1	U FAM: PPIA fillup.1	N FAM: PPIA wasser	U FAM: GFP BPK.1	U FAM: 18S target14.1	U FAM: PPIA target7.1	U FAM: PPIA fillup.1	FAM:	FAM:	FAM:
H	U FAM: GFP pY010.1	U FAM: 18S moxGFP.1	U FAM: PPIA target8.1	U FAM: PPIA reagent only.1	K FAM: GFP calibrator	U FAM: GFP pY010.1	U FAM: 18S moxGFP.1	U FAM: PPIA target8.1	U FAM: PPIA reagent only.1	FAM:	FAM:	FAM:

Monitoring

Monitoring - RawData

All colors

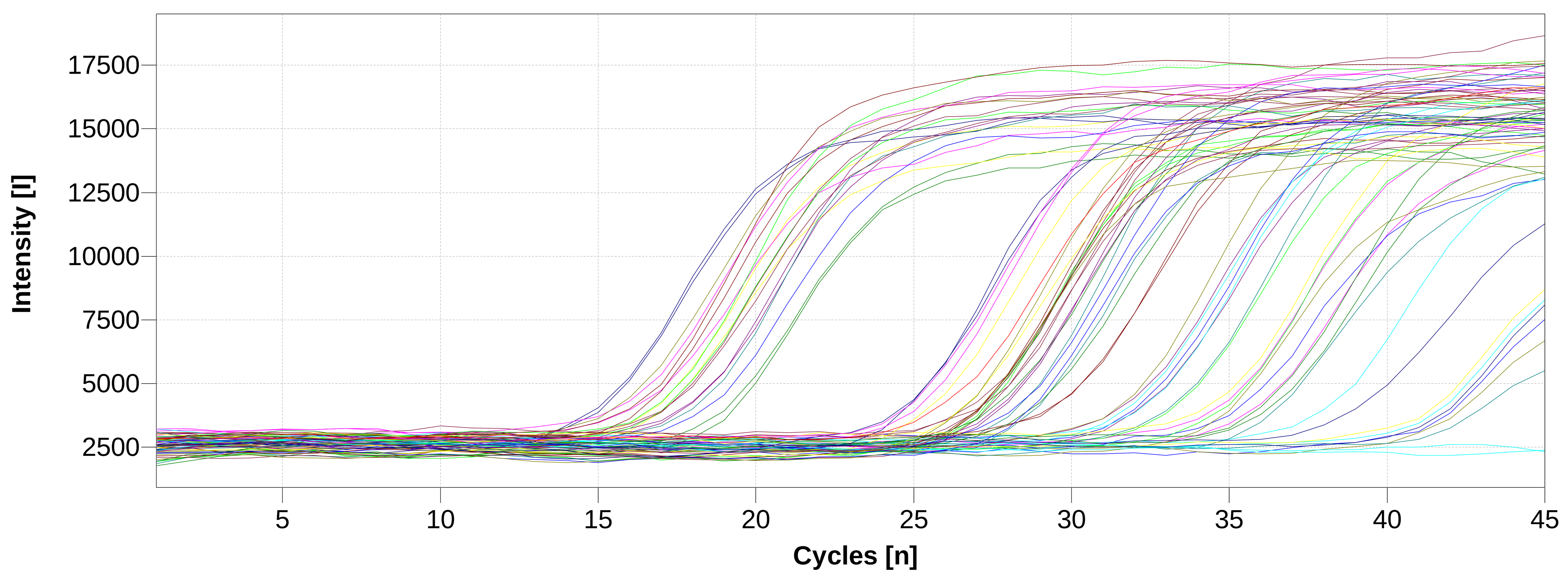

FAM

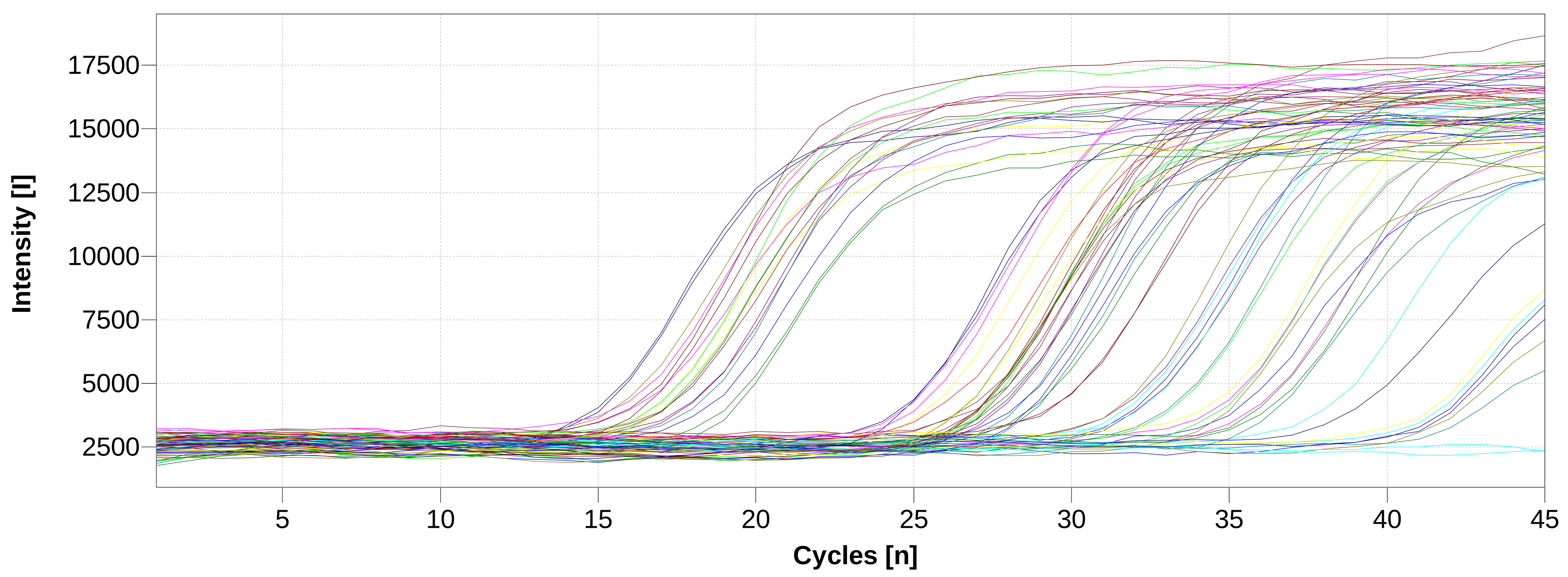

Monitoring

Monitoring - AmplifyData

All colors

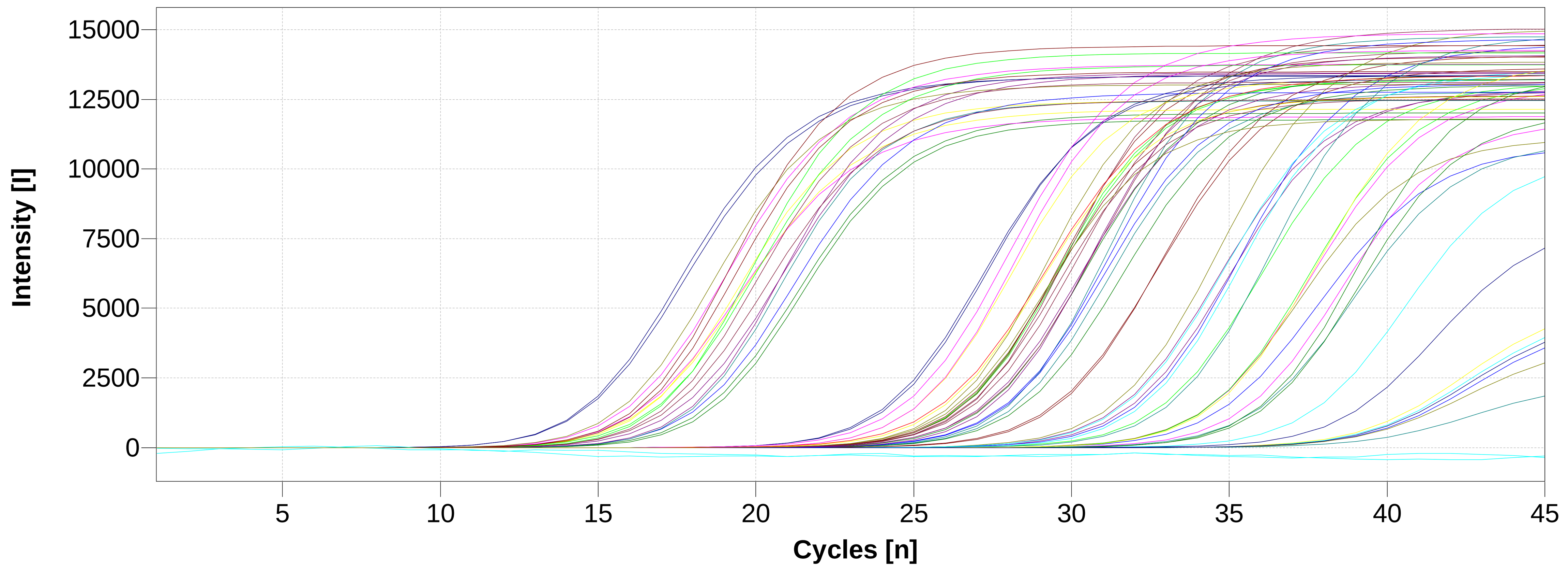

FAM

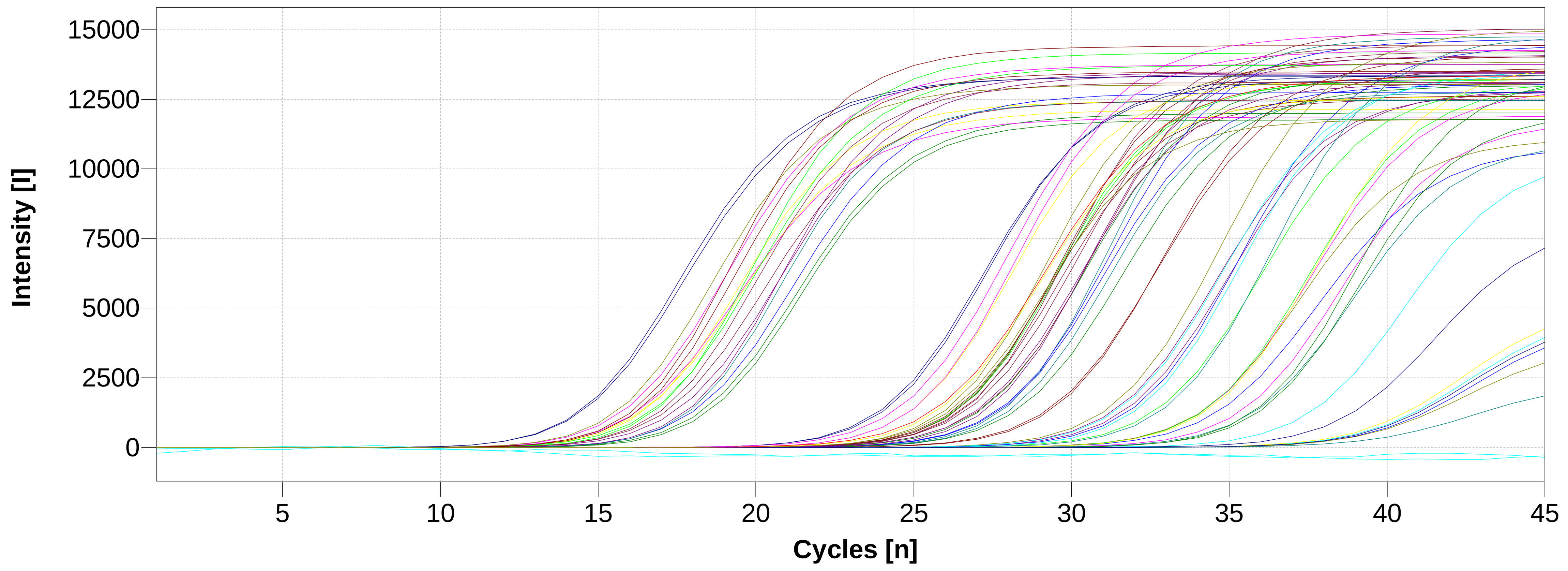

Monitoring

Monitoring - MeltingData

All colors

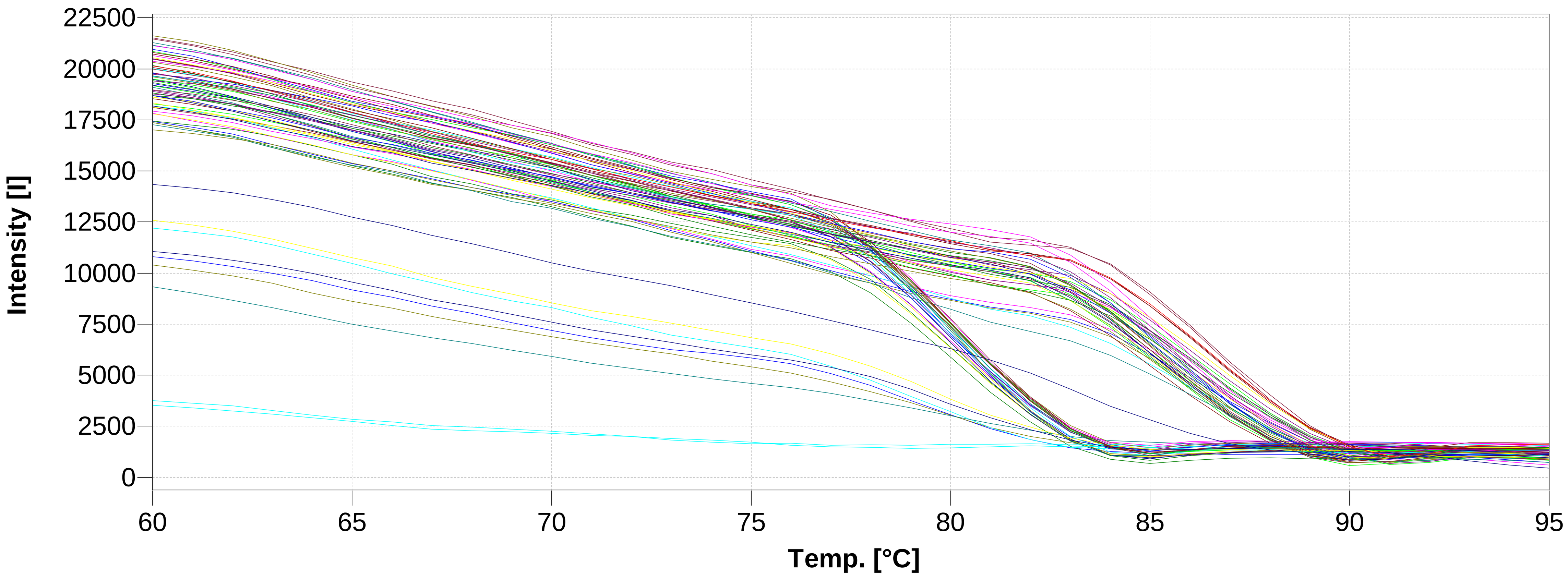

FAM

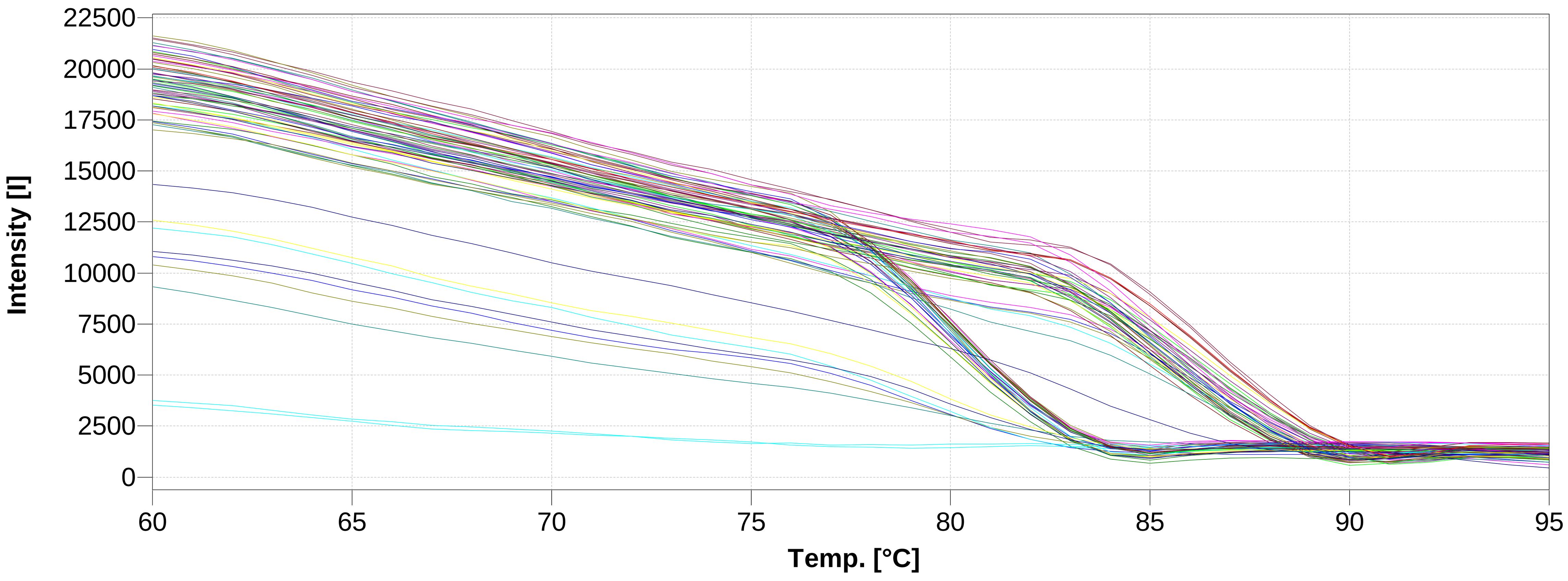

Monitoring

Ct

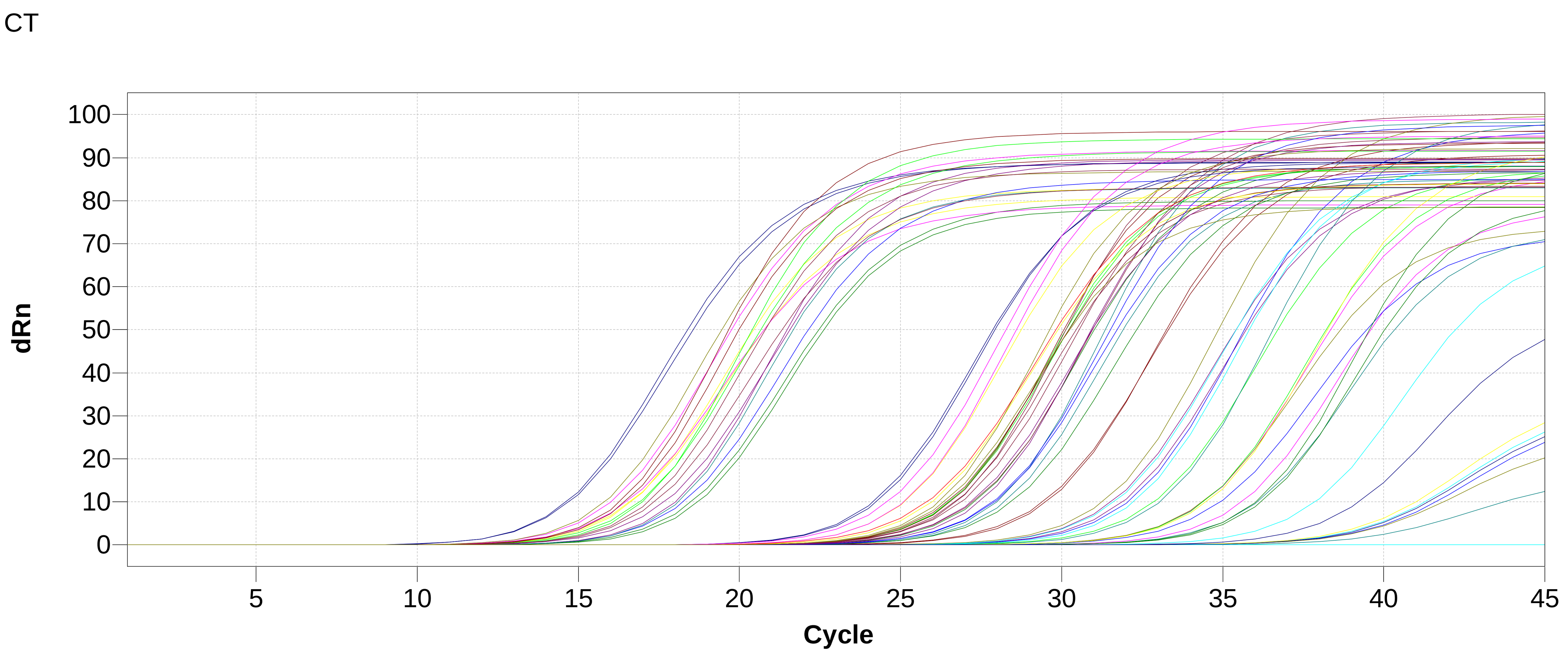

Well		Sample name	Sample type	Dye	Gene	Ct
A1		target7.1	U	FAM	GFP	33,7
A2		fillup.1	U	FAM	GFP	39,21
A4		target13.1	U	FAM	PPIA	24,74
A5		untransfected.1	U	FAM	PPIA	26,2
A6		target7.1	U	FAM	GFP	32,43
A7		fillup.1	U	FAM	GFP	32,92
A9		target13.1	U	FAM	PPIA	24,72
A10		untransfected.1	U	FAM	PPIA	26,39
B1		target8.1	U	FAM	GFP	30,02
B2		reagent only.1	U	FAM	GFP	32,42
B5		wasser	N	FAM	GFP	35,97
B6		target8.1	U	FAM	GFP	29,63
B7		reagent only.1	U	FAM	GFP	39,29
B9		target14.1	U	FAM	PPIA	24,62
C1		target13.1	U	FAM	GFP	32,48
C2		untransfected.1	U	FAM	GFP	34,13
C4		moxGFP.1	U	FAM	PPIA	25,02
C5		wasser	N	FAM	GFP	38,97
C6		target13.1	U	FAM	GFP	30,98
C7		untransfected.1	U	FAM	GFP	40,4
C9		moxGFP.1	U	FAM	PPIA	24,91
D1		target14.1	U	FAM	GFP	27,6
D4		pScaf.1	U	FAM	PPIA	25,42
D6		target14.1	U	FAM	GFP	27,44
D9		pScaf.1	U	FAM	PPIA	26,52
E1		moxGFP.1	U	FAM	GFP	25,3
E4		BPK.1	U	FAM	PPIA	23,3
E6		moxGFP.1	U	FAM	GFP	25,03
E9		BPK.1	U	FAM	PPIA	24,08
F1		pScaf.1	U	FAM	GFP	34,19
F4		pY010.1	U	FAM	PPIA	22,3
F5		wasser	N	FAM	PPIA	No Ct
F6		pScaf.1	U	FAM	GFP	34,31
F9		pY010.1	U	FAM	PPIA	22,41
G1		BPK.1	U	FAM	GFP	32,55
G3		target7.1	U	FAM	PPIA	23,3
G4		fillup.1	U	FAM	PPIA	26,02

Monitoring

Well		Sample name	Sample type	Dye	Gene	Ct
G5		wasser	N	FAM	PPIA	No Ct
G6		BPK.1	U	FAM	GFP	38,68
G8		target7.1	U	FAM	PPIA	22,72
G9		fillup.1	U	FAM	PPIA	26,07
H1		pY010.1	U	FAM	GFP	37,18
H3		target8.1	U	FAM	PPIA	25,65
H4		reagent only.1	U	FAM	PPIA	24,4
H5		calibrator	K	FAM	GFP	23,86
H6		pY010.1	U	FAM	GFP	39,05
H8		target8.1	U	FAM	PPIA	25,3
H9		reagent only.1	U	FAM	PPIA	24,51
A3		pScaf.1	U	FAM	18S	17,03
A8		pScaf.1	U	FAM	18S	16,73
B3		BPK.1	U	FAM	18S	14,98
B4		target14.1	U	FAM	18S	24,76
B8		BPK.1	U	FAM	18S	14,77
C3		pY010.1	U	FAM	18S	12,99
C8		pY010.1	U	FAM	18S	12,95
D2		target7.1	U	FAM	18S	14,62
D3		fillup.1	U	FAM	18S	30,16
D5		wasser	N	FAM	18S	30,37
D7		target7.1	U	FAM	18S	14,22
D8		fillup.1	U	FAM	18S	16,43
E2		target8.1	U	FAM	18S	15,85
E3		reagent only.1	U	FAM	18S	29,35
E5		wasser	N	FAM	18S	29,71
E7		target8.1	U	FAM	18S	16,3
E8		reagent only.1	U	FAM	18S	14,13
F2		target13.1	U	FAM	18S	15,29
F3		untransfected.1	U	FAM	18S	31,18
F7		target13.1	U	FAM	18S	15,12
F8		untransfected.1	U	FAM	18S	16,41
G2		target14.1	U	FAM	18S	14,6
G7		target14.1	U	FAM	18S	14,76
H2		moxGFP.1	U	FAM	18S	15,56
H7		moxGFP.1	U	FAM	18S	15,45

Well		Sample name	Mean Ct	Std.Dev. Ct
A1		target7.1	33,06	0,9
A2		fillup.1	36,07	4,45
A4		target13.1	24,73	0,01
A5		untransfected.1	26,29	0,13
A6		target7.1	33,06	0,9
A7		fillup.1	36,07	4,45
A9		target13.1	24,73	0,01
A10		untransfected.1	26,29	0,13
B1		target8.1	29,83	0,28
B2		reagent only.1	35,85	4,86
B5		wasser	37,47	2,12
B6		target8.1	29,83	0,28
B7		reagent only.1	35,85	4,86
B9		target14.1	24,62	0
C1		target13.1	31,73	1,06

Monitoring

Well		Sample name	Mean Ct	Std.Dev. Ct
C2		untransfected.1	37,26	4,43
C4		moxGFP.1	24,97	0,08
C5		wasser	37,47	2,12
C6		target13.1	31,73	1,06
C7		untransfected.1	37,26	4,43
C9		moxGFP.1	24,97	0,08
D1		target14.1	27,52	0,12
D4		pScaf.1	25,97	0,78
D6		target14.1	27,52	0,12
D9		pScaf.1	25,97	0,78
E1		moxGFP.1	25,17	0,19
E4		BPK.1	23,69	0,56
E6		moxGFP.1	25,17	0,19
E9		BPK.1	23,69	0,56
F1		pScaf.1	34,25	0,08
F4		pY010.1	22,35	0,08
F5		wasser		
F6		pScaf.1	34,25	0,08
F9		pY010.1	22,35	0,08
G1		BPK.1	35,62	4,33
G3		target7.1	23,01	0,41
G4		fillup.1	26,04	0,03
G5		wasser		
G6		BPK.1	35,62	4,33
G8		target7.1	23,01	0,41
G9		fillup.1	26,04	0,03
H1		pY010.1	38,12	1,32
H3		target8.1	25,48	0,25
H4		reagent only.1	24,45	0,08
H5		calibrator	23,86	0
H6		pY010.1	38,12	1,32
H8		target8.1	25,48	0,25
H9		reagent only.1	24,45	0,08
A3		pScaf.1	16,88	0,21
A8		pScaf.1	16,88	0,21
B3		BPK.1	14,87	0,15
B4		target14.1	18,04	5,82
B8		BPK.1	14,87	0,15
C3		pY010.1	12,97	0,03
C8		pY010.1	12,97	0,03
D2		target7.1	14,42	0,28
D3		fillup.1	23,29	9,71
D5		wasser	30,04	0,47
D7		target7.1	14,42	0,28
D8		fillup.1	23,29	9,71
E2		target8.1	16,08	0,32
E3		reagent only.1	21,74	10,77
E5		wasser	30,04	0,47
E7		target8.1	16,08	0,32
E8		reagent only.1	21,74	10,77
F2		target13.1	15,2	0,12
F3		untransfected.1	23,79	10,44
F7		target13.1	15,2	0,12

Monitoring

Well		Sample name	Mean Ct	Std.Dev. Ct
F8		untransfected.1	23,79	10,44
G2		target14.1	18,04	5,82
G7		target14.1	18,04	5,82
H2		moxGFP.1	15,5	0,08
H7		moxGFP.1	15,5	0,08

Tm

DIF - Threshold: 0

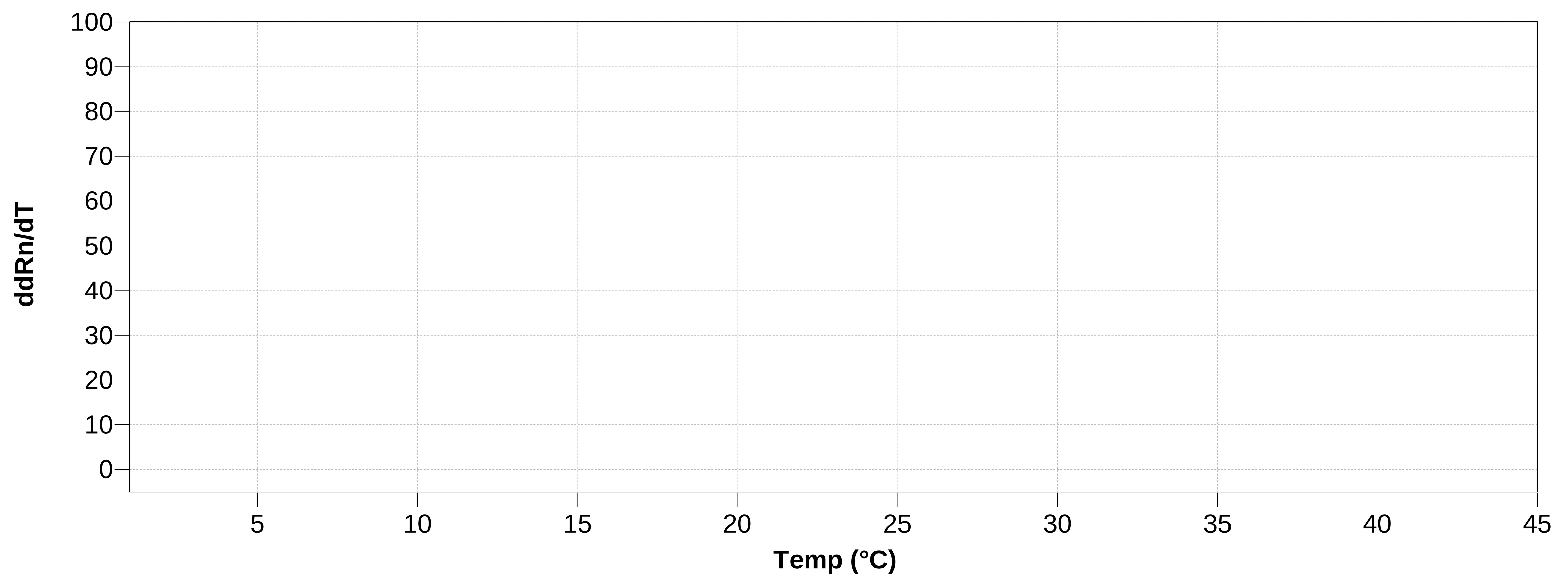

GOI -

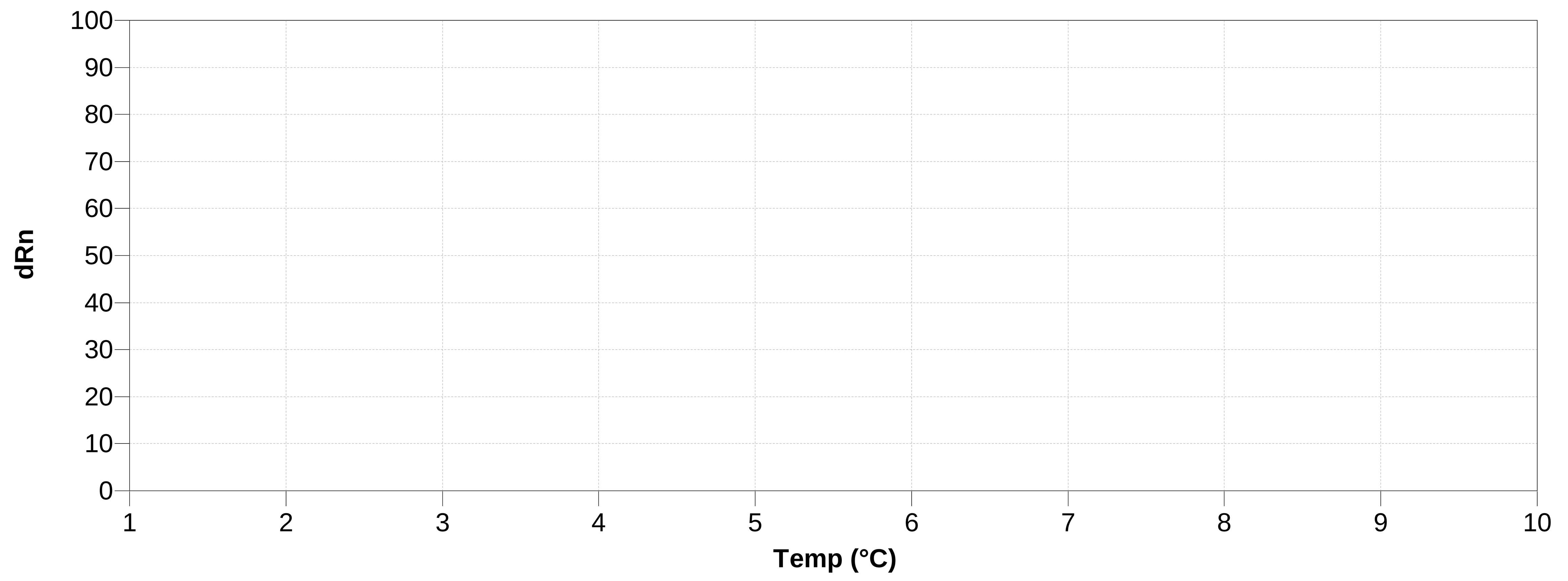

Well		Sample name	Sample type	Tm	Mean Tm	Std.Dev. Mean Tm
A1						
A2						
A3						
A4						
A5						
A6						
A7						
A8						
A9						
A10						
B1						
B2						
B3						
B4						
B5						
B6						
B7						
B8						
B9						
C1						
C2						
C3						
C4						
C5						

Monitoring

Well		Sample name	Sample type	Tm	Mean Tm	Std.Dev. Mean Tm
C6						
C7						
C8						
C9						
D1						
D2						
D3						
D4						
D5						
D6						
D7						
D8						
D9						
E1						
E2						
E3						
E4						
E5						
E6						
E7						
E8						
E9						
F1						
F2						
F3						
F4						
F5						
F6						
F7						
F8						
F9						
G1						
G2						
G3						
G4						
G5						
G6						
G7						
G8						
G9						
H1						
H2						
H3						
H4						
H5						
H6						
H7						
H8						
H9						

ddCt quantification

ddCt quantification

GOI - FAM Threshold: 3,08

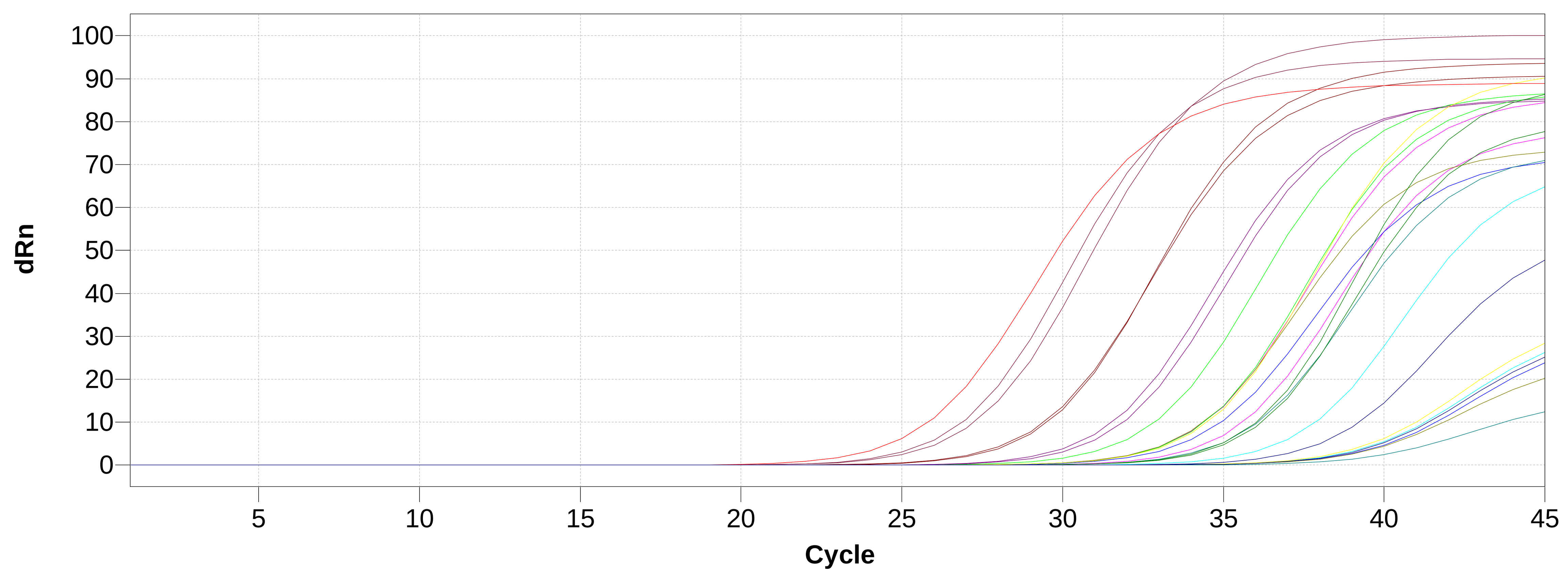

Ref.gene - FAM Threshold: 3,08

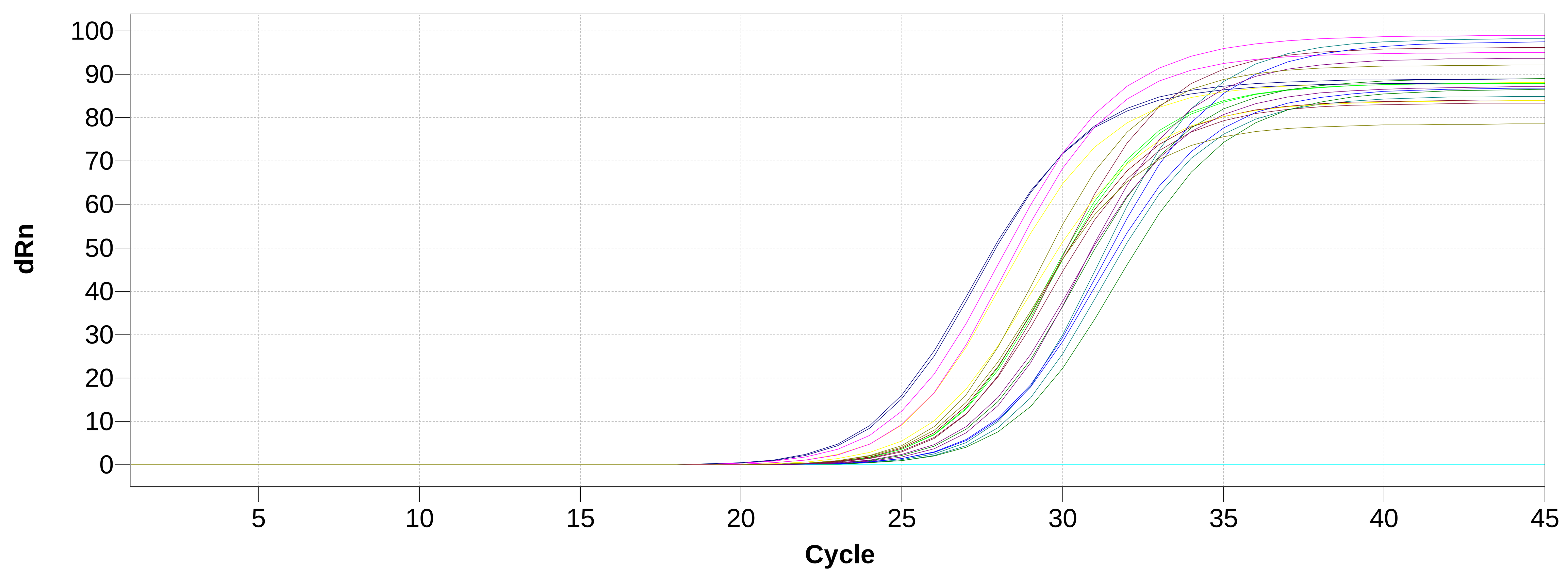

BAR

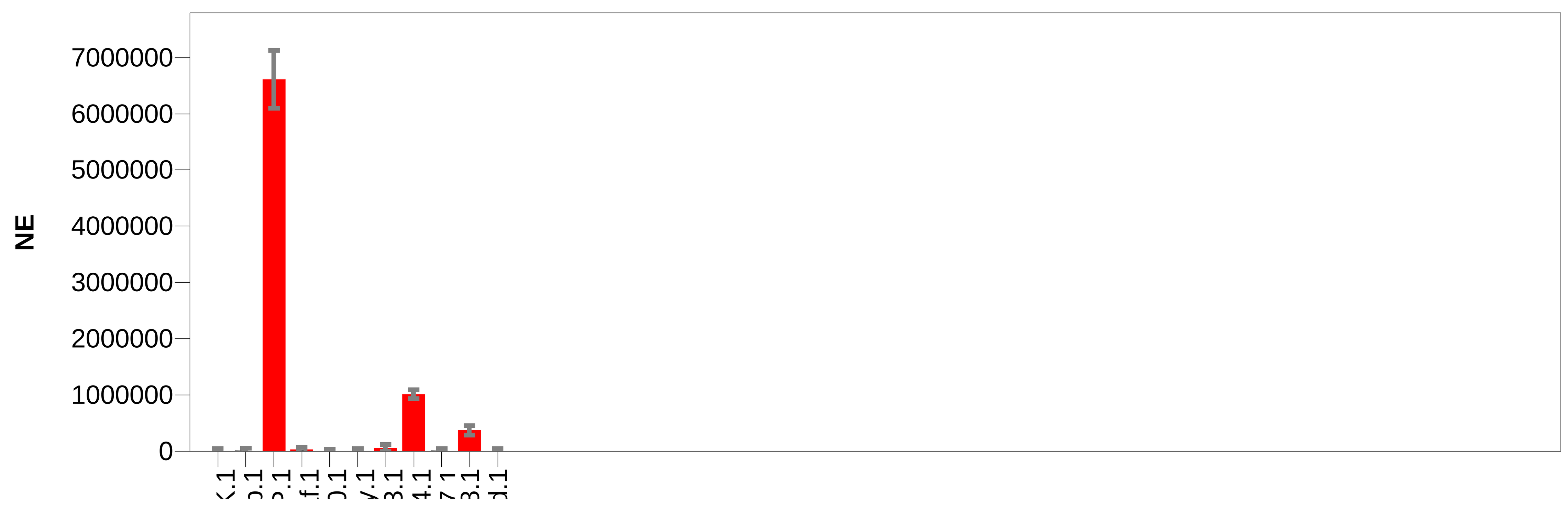

ddCt quantification

ddCt quantification

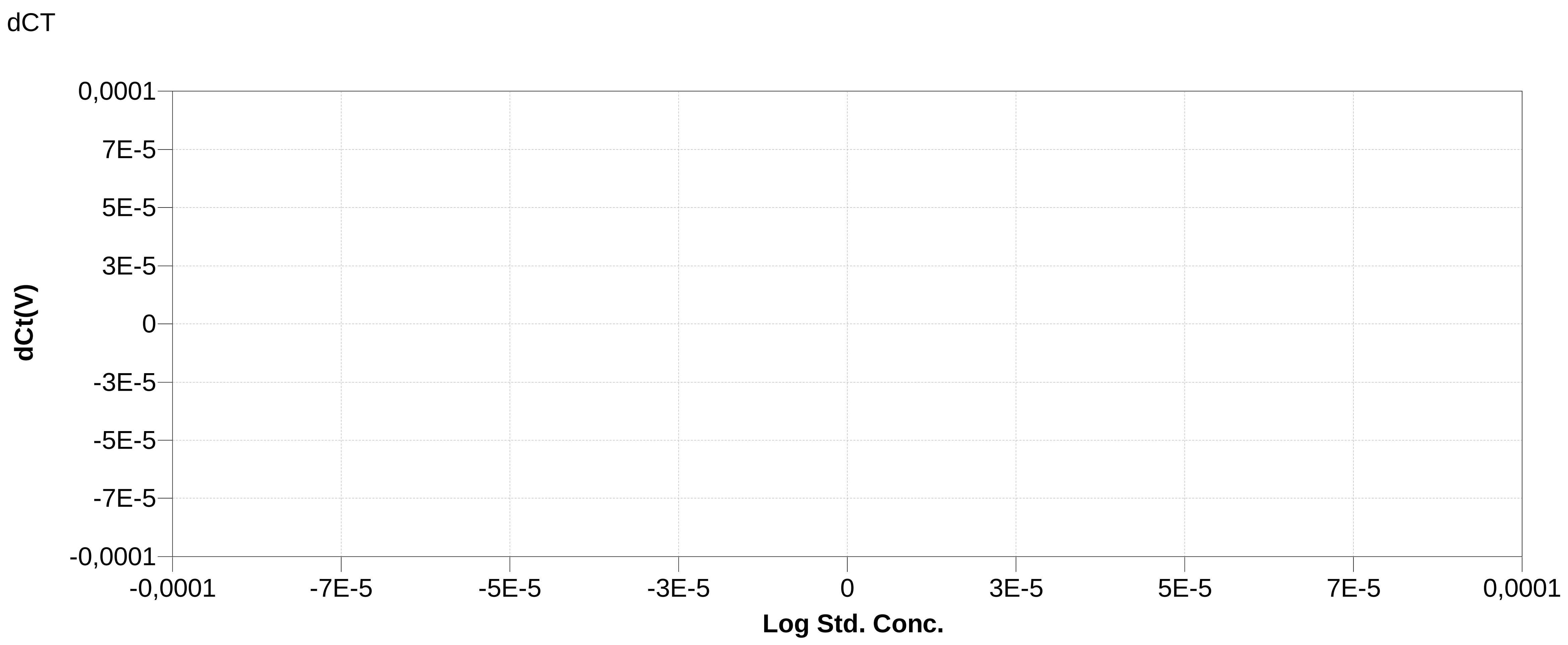

Well		Sample name	Sample type	GOI	Reference gene	Ct GOI
A1		target7.1	U	GFP		33,7
A2		fillup.1	U	GFP		39,21
A3		pScaf.1	U			
A4		target13.1	U		PPIA	
A5		untransfected.1	U		PPIA	
A6		target7.1	U	GFP		32,43
A7		fillup.1	U	GFP		32,92
A8		pScaf.1	U			
A9		target13.1	U		PPIA	
A10		untransfected.1	U		PPIA	
B1		target8.1	U	GFP		30,02
B2		reagent only.1	U	GFP		32,42
B3		BPK.1	U			
B4		target14.1	U			
B5		wasser	N	GFP		35,97
B6		target8.1	U	GFP		29,63
B7		reagent only.1	U	GFP		39,29
B8		BPK.1	U			
B9		target14.1	U		PPIA	
C1		target13.1	U	GFP		32,48
C2		untransfected.1	U	GFP		34,13
C3		pY010.1	U			
C4		moxGFP.1	U		PPIA	
C5		wasser	N	GFP		38,97
C6		target13.1	U	GFP		30,98
C7		untransfected.1	U	GFP		40,4
C8		pY010.1	U			
C9		moxGFP.1	U		PPIA	
D1		target14.1	U	GFP		27,6
D2		target7.1	U			
D3		fillup.1	U			
D4		pScaf.1	U		PPIA	
D5		wasser	N			
D6		target14.1	U	GFP		27,44
D7		target7.1	U			
D8		fillup.1	U			
D9		pScaf.1	U		PPIA	

ddCt quantification

Well		Sample name	Sample type	GOI	Reference gene	Ct GOI
E1		moxGFP.1	U	GFP		25,3
E2		target8.1	U			
E3		reagent only.1	U			
E4		BPK.1	U		PPIA	
E5		wasser	N			
E6		moxGFP.1	U	GFP		25,03
E7		target8.1	U			
E8		reagent only.1	U			
E9		BPK.1	U		PPIA	
F1		pScaf.1	U	GFP		34,19
F2		target13.1	U			
F3		untransfected.1	U			
F4		pY010.1	U		PPIA	
F5		wasser	N		PPIA	
F6		pScaf.1	U	GFP		34,31
F7		target13.1	U			
F8		untransfected.1	U			
F9		pY010.1	U		PPIA	
G1		BPK.1	U	GFP		32,55
G2		target14.1	U			
G3		target7.1	U		PPIA	
G4		fillup.1	U		PPIA	
G5		wasser	N		PPIA	
G6		BPK.1	U	GFP		38,68
G7		target14.1	U			
G8		target7.1	U		PPIA	
G9		fillup.1	U		PPIA	
H1		pY010.1	U	GFP		37,18
H2		moxGFP.1	U			
H3		target8.1	U		PPIA	
H4		reagent only.1	U		PPIA	
H5		calibrator	K	GFP		23,86
H6		pY010.1	U	GFP		39,05
H7		moxGFP.1	U			
H8		target8.1	U		PPIA	
H9		reagent only.1	U		PPIA	

Well		Sample name	Ct Ref. gene	Mean Ct GOI	Mean Ct Ref.gene	RQ GOI
A1		target7.1		33,07		0
A2		fillup.1		36,07		0
A3		pScaf.1				
A4		target13.1	24,74		24,73	
A5		untransfected.1	26,2		26,29	
A6		target7.1		33,07		0
A7		fillup.1		36,07		0
A8		pScaf.1				
A9		target13.1	24,72		24,73	
A10		untransfected.1	26,39		26,29	
B1		target8.1		29,83		0,02
B2		reagent only.1		35,85		0
B3		BPK.1				
B4		target14.1				
B5		wasser		37,47		0

ddCt quantification

Well		Sample name	Ct Ref. gene	Mean Ct GOI	Mean Ct Ref.gene	RQ GOI
B6		target8.1		29,83		0,02
B7		reagent only.1		35,85		0
B8		BPK.1				
B9		target14.1	24,62		24,62	
C1		target13.1		31,73		0
C2		untransfected.1		37,26		0
C3		pY010.1				
C4		moxGFP.1	25,02		24,97	
C5		wasser		37,47		0
C6		target13.1		31,73		0
C7		untransfected.1		37,26		0
C8		pY010.1				
C9		moxGFP.1	24,91		24,97	
D1		target14.1		27,52		0,08
D2		target7.1				
D3		fillup.1				
D4		pScaf.1	25,42		25,97	
D5		wasser				
D6		target14.1		27,52		0,08
D7		target7.1				
D8		fillup.1				
D9		pScaf.1	26,52		25,97	
E1		moxGFP.1		25,17		0,4
E2		target8.1				
E3		reagent only.1				
E4		BPK.1	23,3		23,69	
E5		wasser				
E6		moxGFP.1		25,17		0,4
E7		target8.1				
E8		reagent only.1				
E9		BPK.1	24,08		23,69	
F1		pScaf.1		34,25		0
F2		target13.1				
F3		untransfected.1				
F4		pY010.1	22,3		22,35	
F5		wasser	No Ct			
F6		pScaf.1		34,25		0
F7		target13.1				
F8		untransfected.1				
F9		pY010.1	22,41		22,35	
G1		BPK.1		35,62		0
G2		target14.1				
G3		target7.1	23,3		23,01	
G4		fillup.1	26,02		26,04	
G5		wasser	No Ct			
G6		BPK.1		35,62		0
G7		target14.1				
G8		target7.1	22,72		23,01	
G9		fillup.1	26,07		26,04	
H1		pY010.1		38,12		0
H2		moxGFP.1				
H3		target8.1	25,65		25,48	
H4		reagent only.1	24,4		24,45	

ddCt quantification

Well		Sample name	Ct Ref. gene	Mean Ct GOI	Mean Ct Ref.gene	RQ GOI
H5		calibrator		23,86		1
H6		pY010.1		38,12		0
H7		moxGFP.1				
H8		target8.1	25,3		25,48	
H9		reagent only.1	24,51		24,45	

Well		Sample name	RQ Ref.gene	Norm. Expression	Std.Dev. RQ GOI	Std.Dev. RQ Ref.gene
A1		target7.1		7131,2398	0	
A2		fillup.1		7302,4764	0	
A3		pScaf.1				
A4		target13.1	0	59464,4843		0
A5		untransfected.1	0	3783,4322		0
A6		target7.1		7131,2398	0	
A7		fillup.1		7302,4764	0	
A8		pScaf.1				
A9		target13.1	0	59464,4843		0
A10		untransfected.1	0	3783,4322		0
B1		target8.1		372693,1286	0	
B2		reagent only.1		2805,322	0	
B3		BPK.1				
B4		target14.1				
B5		wasser			0	
B6		target8.1		372693,1286	0	
B7		reagent only.1		2805,322	0	
B8		BPK.1				
B9		target14.1	0	1014329,4325		0
C1		target13.1		59464,4843	0	
C2		untransfected.1		3783,4322	0	
C3		pY010.1				
C4		moxGFP.1	0	6611421,2395		0
C5		wasser			0	
C6		target13.1		59464,4843	0	
C7		untransfected.1		3783,4322	0	
C8		pY010.1				
C9		moxGFP.1	0	6611421,2395		0
D1		target14.1		1014329,4325	0,01	
D2		target7.1				
D3		fillup.1				
D4		pScaf.1	0	24386,0508		0
D5		wasser				
D6		target14.1		1014329,4325	0,01	
D7		target7.1				
D8		fillup.1				
D9		pScaf.1	0	24386,0508		0
E1		moxGFP.1		6611421,2395	0,05	
E2		target8.1				
E3		reagent only.1				
E4		BPK.1	0	1951,9305		0
E5		wasser				
E6		moxGFP.1		6611421,2395	0,05	
E7		target8.1				
E8		reagent only.1				
E9		BPK.1	0	1951,9305		0

ddCt quantification

Well		Sample name	RQ Ref.gene	Norm. Expression	Std.Dev. RQ GOI	Std.Dev. RQ Ref.gene
F1		pScaf.1		24386,0508	0	
F2		target13.1				
F3		untransfected.1				
F4		pY010.1	0	136,7459		0
F5		wasser				
F6		pScaf.1		24386,0508	0	
F7		target13.1				
F8		untransfected.1				
F9		pY010.1	0	136,7459		0
G1		BPK.1		1951,9305	0	
G2		target14.1				
G3		target7.1	0	7131,2398		0
G4		fillup.1	0	7302,4764		0
G5		wasser				
G6		BPK.1		1951,9305	0	
G7		target14.1				
G8		target7.1	0	7131,2398		0
G9		fillup.1	0	7302,4764		0
H1		pY010.1		136,7459	0	
H2		moxGFP.1				
H3		target8.1	0	372693,1286		0
H4		reagent only.1	0	2805,322		0
H5		calibrator			0	
H6		pY010.1		136,7459	0	
H7		moxGFP.1				
H8		target8.1	0	372693,1286		0
H9		reagent only.1	0	2805,322		0

Well		Sample name	Std.Dev. Norm. Expression	dCt (Ref.Gen – GOI)
A1		target7.1	4882,7271	-10,06
A2		fillup.1	22514,8713	-10,02
A3		pScaf.1		
A4		target13.1		
A5		untransfected.1		
A6		target7.1	4882,7271	-10,06
A7		fillup.1	22514,8713	-10,02
A8		pScaf.1		
A9		target13.1		
A10		untransfected.1		
B1		target8.1	96278,961	-4,35
B2		reagent only.1	9453,6371	-11,4
B3		BPK.1		
B4		target14.1		
B5		wasser		
B6		target8.1	96278,961	-4,35
B7		reagent only.1	9453,6371	-11,4
B8		BPK.1		
B9		target14.1		
C1		target13.1	43704,2965	-7
C2		untransfected.1	11629,3115	-10,97
C3		pY010.1		
C4		moxGFP.1		
C5		wasser		

ddCt quantification

Well		Sample name	Std.Dev. Norm. Expression	dCt (Ref.Gen – GOI)
C6		target13.1	43704,2965	-7
C7		untransfected.1	11629,3115	-10,97
C8		pY010.1		
C9		moxGFP.1		
D1		target14.1	81820,1884	-2,91
D2		target7.1		
D3		fillup.1		
D4		pScaf.1		
D5		wasser		
D6		target14.1	81820,1884	-2,91
D7		target7.1		
D8		fillup.1		
D9		pScaf.1		
E1		moxGFP.1	946583,7227	-0,2
E2		target8.1		
E3		reagent only.1		
E4		BPK.1		
E5		wasser		
E6		moxGFP.1	946583,7227	-0,2
E7		target8.1		
E8		reagent only.1		
E9		BPK.1		
F1		pScaf.1	13196,5988	-8,28
F2		target13.1		
F3		untransfected.1		
F4		pY010.1		
F5		wasser		
F6		pScaf.1	13196,5988	-8,28
F7		target13.1		
F8		untransfected.1		
F9		pY010.1		
G1		BPK.1	5908,2196	-11,93
G2		target14.1		
G3		target7.1		
G4		fillup.1		
G5		wasser		
G6		BPK.1	5908,2196	-11,93
G7		target14.1		
G8		target7.1		
G9		fillup.1		
H1		pY010.1	125,7444	-15,76
H2		moxGFP.1		
H3		target8.1		
H4		reagent only.1		
H5		calibrator		
H6		pY010.1	125,7444	-15,76
H7		moxGFP.1		
H8		target8.1		
H9		reagent only.1		

Experiment

Title: TEMPLATE - qPCR

User: Administrator

Program

Preheat lid: ⊕

Lid temp. °C: 100

Step	Scan	Temp (°C)	Time (m:s)	Goto	Loops	+/- Temp (°C)	+/- Time (s)	Ramp (°C/s)
1		95,0	00:30	0	0	0,0	0	8,0
2		95,0	01:00	0	0	0,0	0	8,0
3		95,0	00:10	0	0	0,0	0	8,0
4		60,0	00:30	0	0	0,0	0	6,0
5	◆	70,0	00:03	3	44	0,0	0	6,0

Melting curve

Active: ⊕

Start temp. (°C)	End temp. (°C)	Increment (°C)	Equilibration (s)	Ramp (°C/s)
60,0	95,0	4,0	15	0,1

Scan

Meas. repeats: 3

from column: 1

Color compensation: ⊖

to column: 10

Pos.	Channel	Excitation	Detection	Dye	Gain	Measurement	Pass. Ref.
1	Blue	470	520	FAM	5	⊕	⊖
2	Green	515	545	JOE	5	⊖	⊖
3	Orange	565	605	ROX	5	⊖	⊖
4	Red	630	670	Cy5	5	⊖	⊖
5	Yellow	535	580	TAMRA	5	⊖	⊖

Control: Block Control

Start: 05.03.2026 18:21:48

End: 05.03.2026 19:47:28

Comment:

Settings

General

Title:	TEMPLATE - qPCR
Operator:	
Start:	05.03.2026 18:21:48
End:	05.03.2026 19:47:28
Comment:	

Settings

Thermal Cycler

Block type:	28
Lid temp.:	100°C
Hot start:	Yes
Control:	Block Control
Standby:	No
Block temp.:	12°C

Melting curve

Start temp.:	60°C
End temp.:	95°C
Gradient:	1°C
Ramp:	0,1°C/s
Equilibration:	15s
Active:	Yes

Program

Step	Scan	Temp (°C)	Time (m:s)	Goto	Loops	+/- Temp (°C)	+/- Time (s)	Ramp (°C/s)
1		95,0	00:30	0	0	0,0	0	8,0
1		95,0	00:30	0	0	0,0	0	8,0
1		95,0	00:30	0	0	0,0	0	8,0
1		95,0	00:30	0	0	0,0	0	8,0
1		95,0	00:30	0	0	0,0	0	8,0

Settings

Scan

Meas. repeats:	3
Color compensation:	No
from column:	1
to column:	10

Modules

Pos.	Channel	Excitation	Detection	Dye	Gain	Measurement	Pass. Ref.
1	Blue	470	520	FAM	5	⊕	⊖
2	Green	515	545	JOE	5	⊖	⊖
3	Orange	565	605	ROX	5	⊖	⊖
4	Red	630	670	Cy5	5	⊖	⊖
5	Yellow	535	580	TAMRA	5	⊖	⊖

Settings

Samples

	1	2	3	4	5	6	7	8	9	10	11	12
A	U FAM: GFP target7.2	U FAM: GFP fillup.2	U FAM: 18S pScaf.2	U FAM: PPIA target13.2	U FAM: PPIA untransfected	U FAM: GFP target7.2	U FAM: GFP fillup.2	U FAM: 18S pScaf.2	U FAM: PPIA target13.2	U FAM: PPIA untransfected	FAM:	FAM:
B	U FAM: GFP target8.2	U FAM: GFP reagent only.	U FAM: 18S BPK.2	U FAM: 18S target14.2	N FAM: GFP wasser	U FAM: GFP target8.2	U FAM: GFP reagent only.	U FAM: 18S BPK.2	U FAM: PPIA target14.2	FAM:	FAM:	FAM:
C	U FAM: GFP target13.2	U FAM: GFP untransfected	U FAM: 18S pY010.2	U FAM: PPIA moxGFP.2	N FAM: GFP wasser	U FAM: GFP target13.2	U FAM: GFP untransfected	U FAM: 18S pY010.2	U FAM: PPIA moxGFP.2	FAM:	FAM:	FAM:
D	U FAM: GFP target14.2	U FAM: 18S target7.2	U FAM: 18S fillup.2	U FAM: PPIA pScaf.2	N FAM: 18S wasser	U FAM: GFP target14.2	U FAM: 18S target7.2	U FAM: 18S fillup.2	U FAM: PPIA pScaf.2	FAM:	FAM:	FAM:
E	U FAM: GFP moxGFP.2	U FAM: 18S target8.2	U FAM: 18S reagent only.	U FAM: PPIA BPK.2	N FAM: 18S wasser	U FAM: GFP moxGFP.2	U FAM: 18S target8.2	U FAM: 18S reagent only.	U FAM: PPIA BPK.2	FAM:	FAM:	FAM:
F	U FAM: GFP pScaf.2	U FAM: 18S target13.2	U FAM: 18S untransfected	U FAM: PPIA pY010.2	N FAM: PPIA wasser	U FAM: GFP pScaf.2	U FAM: 18S target13.2	U FAM: 18S untransfected	U FAM: PPIA pY010.2	FAM:	FAM:	FAM:
G	U FAM: GFP BPK.2	U FAM: 18S target14.2	U FAM: PPIA target7.2	U FAM: PPIA fillup.2	N FAM: PPIA wasser	U FAM: GFP BPK.2	U FAM: 18S target14.2	U FAM: PPIA target7.2	U FAM: PPIA fillup.2	FAM:	FAM:	FAM:
H	U FAM: GFP pY010.2	U FAM: 18S moxGFP.2	U FAM: PPIA target8.2	U FAM: PPIA reagent only.2	K FAM: GFP calibrator	U FAM: GFP pY010.2	U FAM: 18S moxGFP.2	U FAM: PPIA target8.2	U FAM: PPIA reagent only.2	FAM:	FAM:	FAM:

Monitoring

Monitoring - RawData

All colors

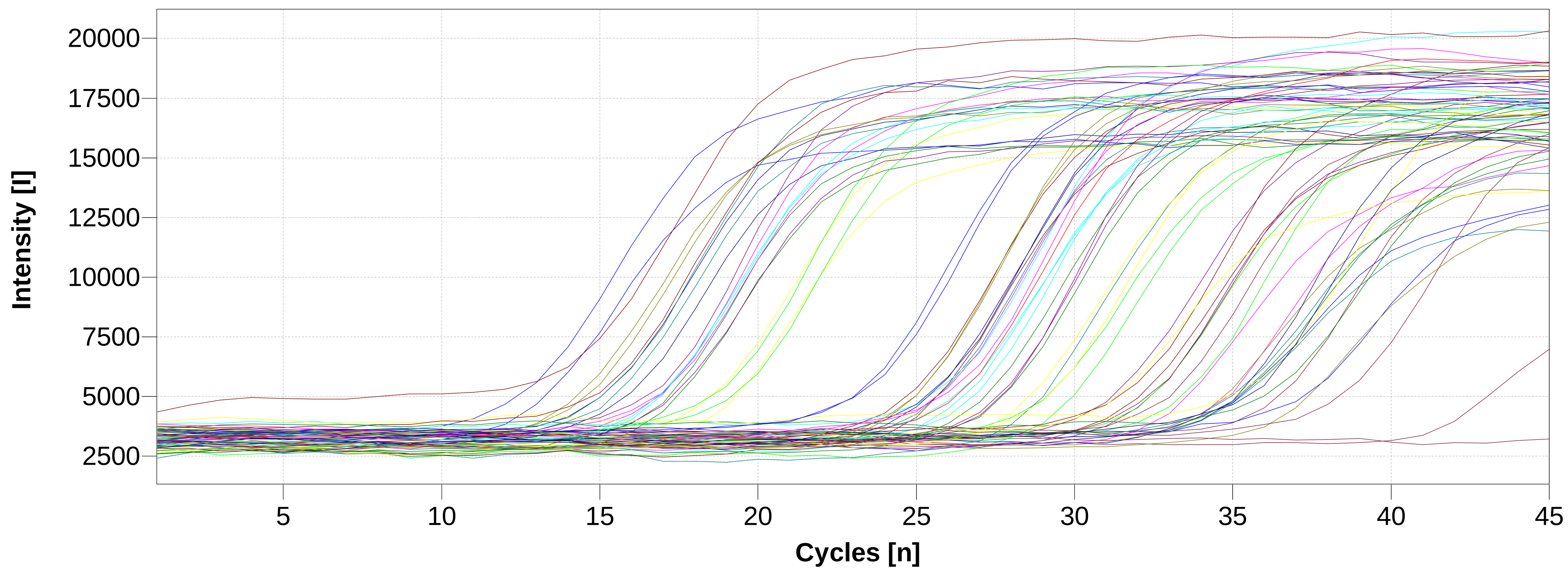

FAM

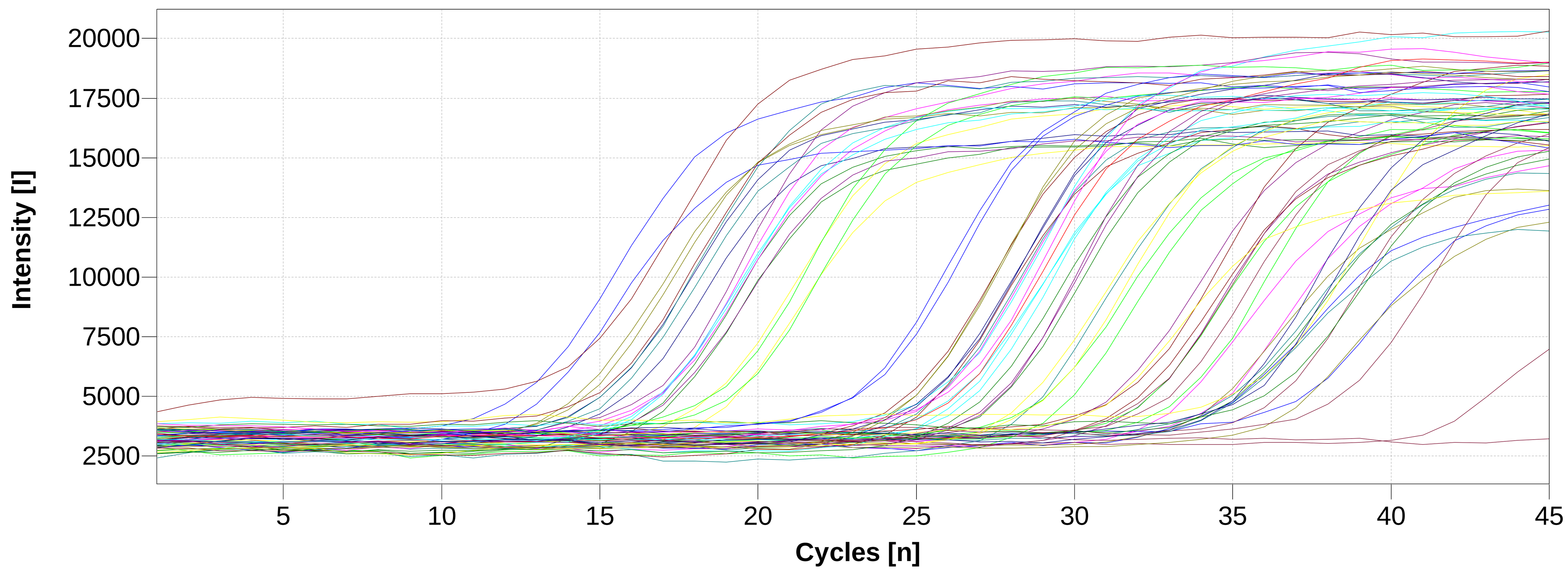

Monitoring - AmplifyData

All colors

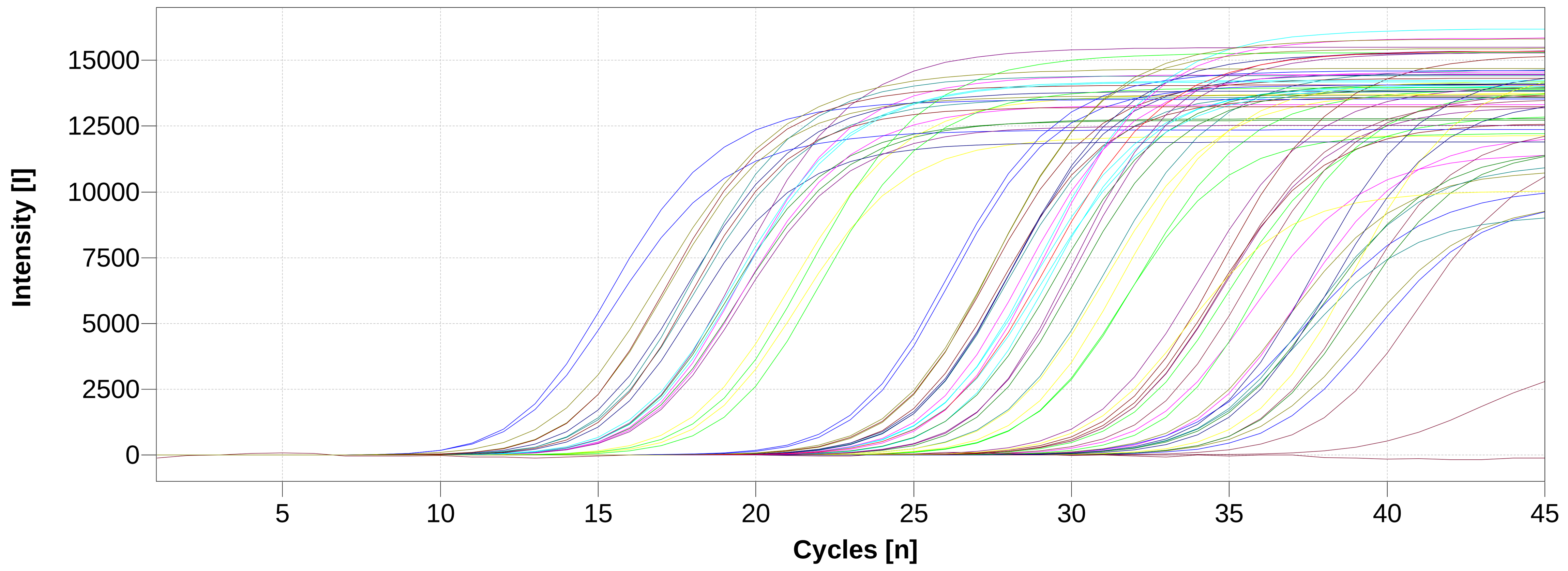

FAM

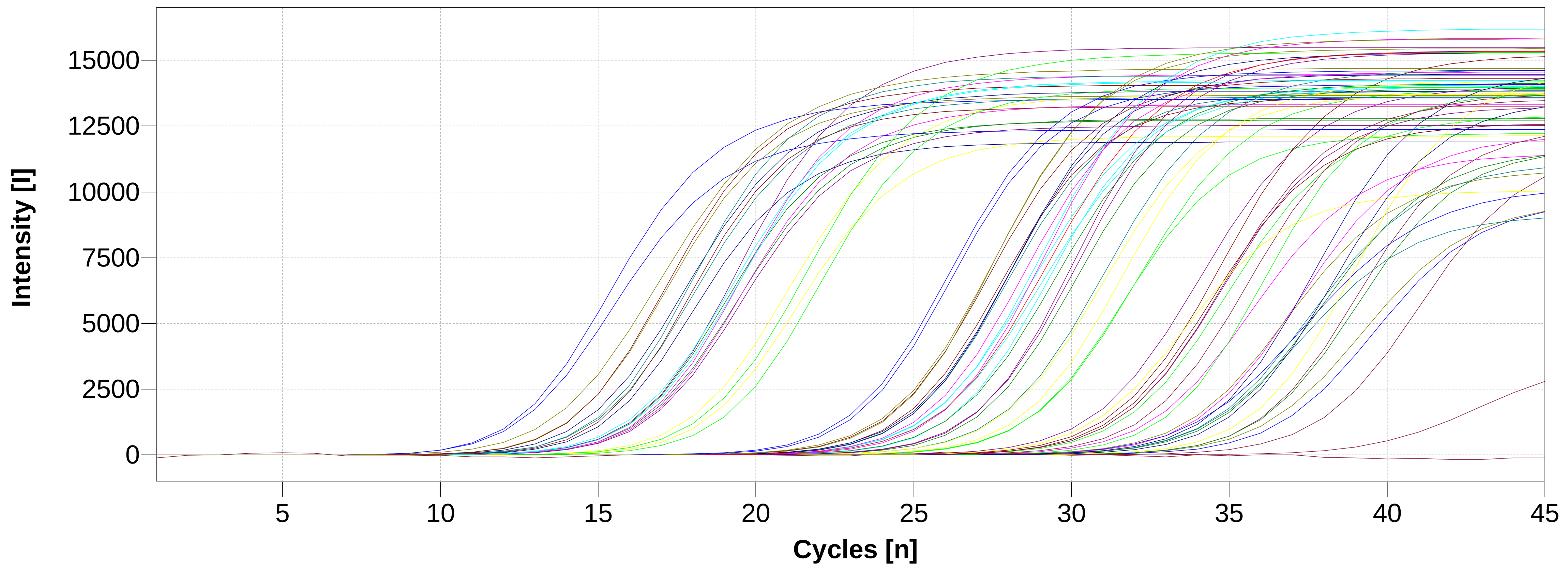

Monitoring

Monitoring - MeltingData

All colors

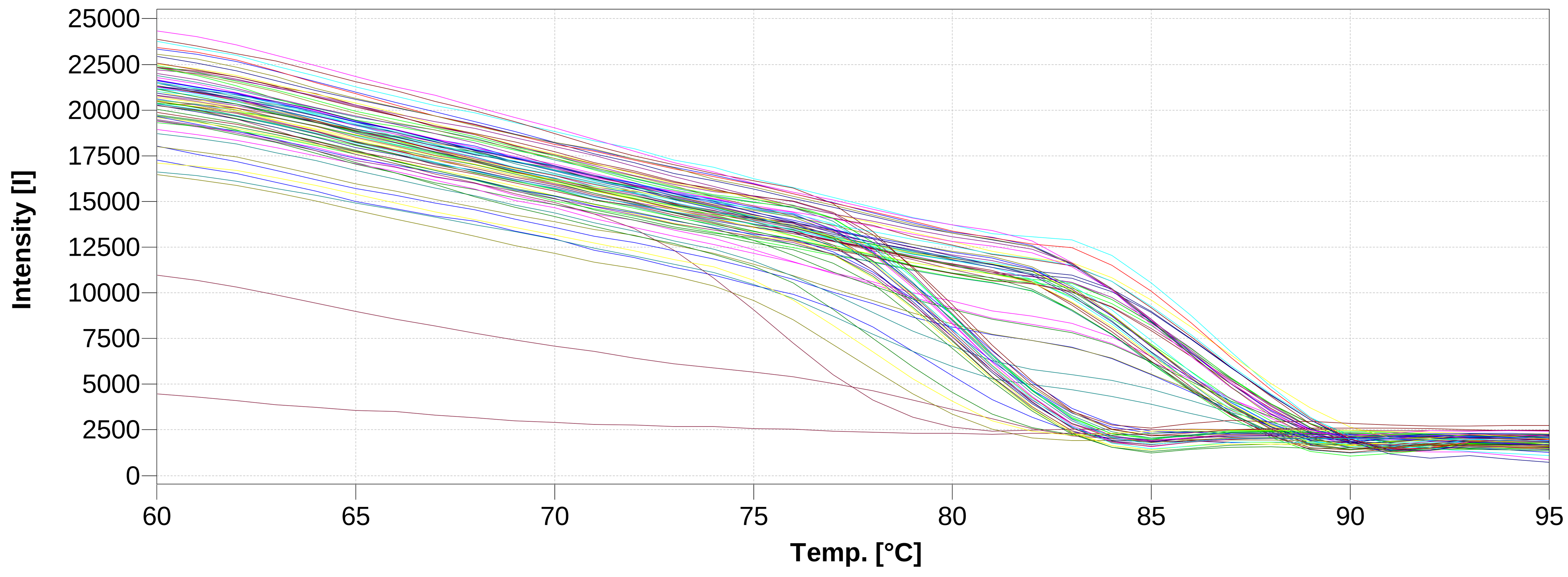

FAM

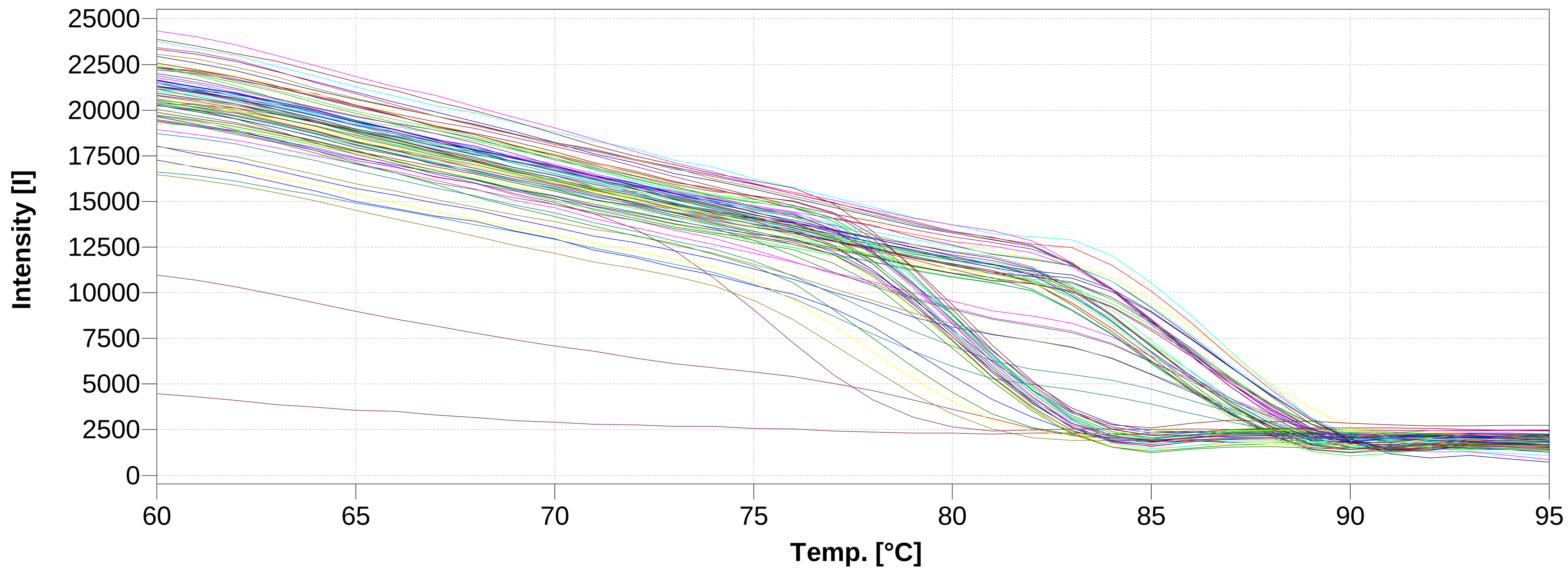

Monitoring

Ct

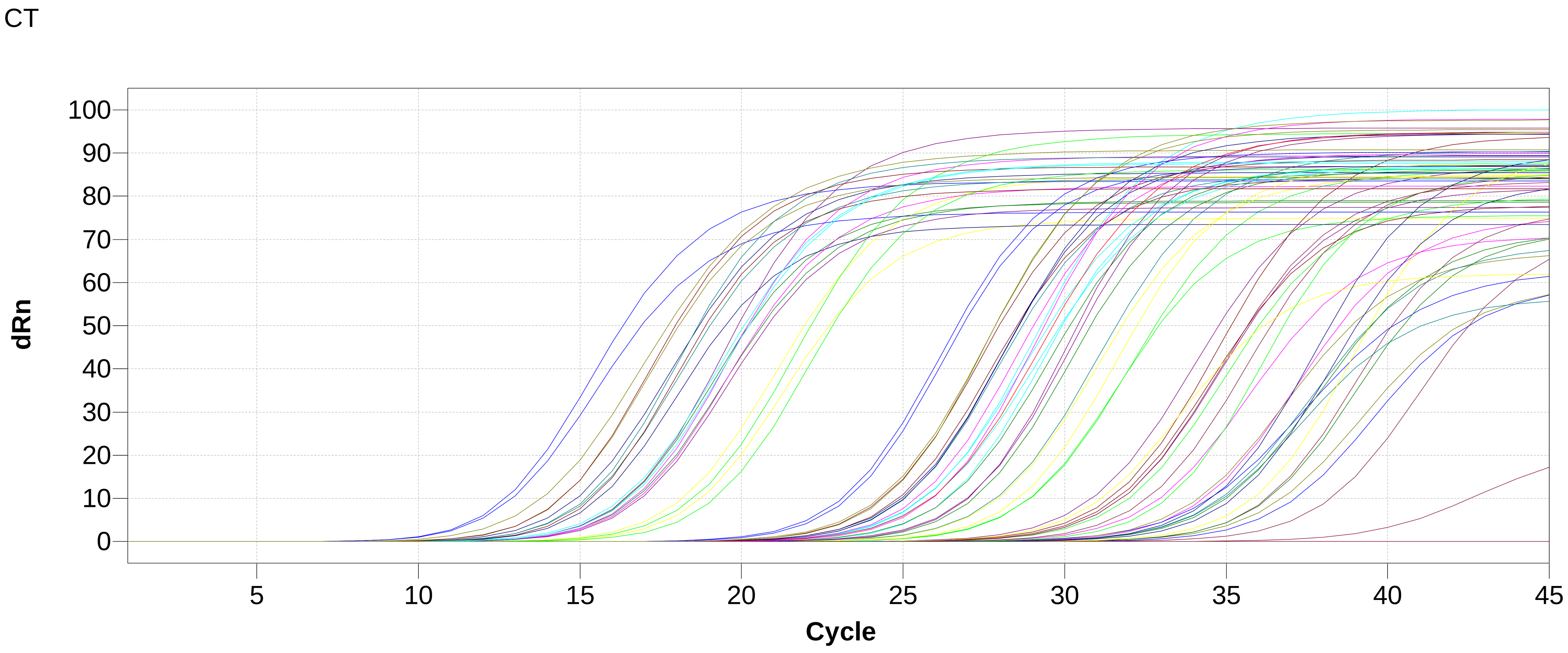

Well		Sample name	Sample type	Dye	Gene	Ct
A1		target7.2	U	FAM	GFP	31,64
A2		fillup.2	U	FAM	GFP	34,39
A4		target13.2	U	FAM	PPIA	26,3
A5		untransfected.2	U	FAM	PPIA	22,56
A6		target7.2	U	FAM	GFP	30,25
A7		fillup.2	U	FAM	GFP	31,47
A9		target13.2	U	FAM	PPIA	26,37
A10		untransfected.2	U	FAM	PPIA	25,27
B1		target8.2	U	FAM	GFP	28,17
B2		reagent only.2	U	FAM	GFP	31,38
B5		wasser	N	FAM	GFP	39,05
B6		target8.2	U	FAM	GFP	29,15
B7		reagent only.2	U	FAM	GFP	34,01
B9		target14.2	U	FAM	PPIA	22,39
C1		target13.2	U	FAM	GFP	30,69
C2		untransfected.2	U	FAM	GFP	32,11
C4		moxGFP.2	U	FAM	PPIA	24,01
C5		wasser	N	FAM	GFP	33,74
C6		target13.2	U	FAM	GFP	29,32
C7		untransfected.2	U	FAM	GFP	32,12
C9		moxGFP.2	U	FAM	PPIA	23,04
D1		target14.2	U	FAM	GFP	28,74
D4		pScaf.2	U	FAM	PPIA	24,73
D6		target14.2	U	FAM	GFP	29,05
D9		pScaf.2	U	FAM	PPIA	23,88
E1		moxGFP.2	U	FAM	GFP	23,07
E4		BPK.2	U	FAM	PPIA	26,1
E6		moxGFP.2	U	FAM	GFP	23,19
E9		BPK.2	U	FAM	PPIA	25,34
F1		pScaf.2	U	FAM	GFP	33,72
F4		pY010.2	U	FAM	PPIA	22,59
F5		wasser	N	FAM	PPIA	No Ct
F6		pScaf.2	U	FAM	GFP	32,24
F9		pY010.2	U	FAM	PPIA	22,4
G1		BPK.2	U	FAM	GFP	33,27
G3		target7.2	U	FAM	PPIA	23
G4		fillup.2	U	FAM	PPIA	20,96

Monitoring

Well		Sample name	Sample type	Dye	Gene	Ct
G5		wasser	N	FAM	PPIA	35,54
G6		BPK.2	U	FAM	GFP	28,47
G8		target7.2	U	FAM	PPIA	23,4
G9		fillup.2	U	FAM	PPIA	20,66
H1		pY010.2	U	FAM	GFP	32,56
H3		target8.2	U	FAM	PPIA	24,56
H4		reagent only.2	U	FAM	PPIA	22,03
H5		calibrator	K	FAM	GFP	23,23
H6		pY010.2	U	FAM	GFP	32,01
H8		target8.2	U	FAM	PPIA	24,43
H9		reagent only.2	U	FAM	PPIA	21,72
A3		pScaf.2	U	FAM	18S	14,19
A8		pScaf.2	U	FAM	18S	14,33
B3		BPK.2	U	FAM	18S	15,77
B4		target14.2	U	FAM	18S	21,95
B8		BPK.2	U	FAM	18S	16,36
C3		pY010.2	U	FAM	18S	12,53
C8		pY010.2	U	FAM	18S	13,34
D2		target7.2	U	FAM	18S	14,33
D3		fillup.2	U	FAM	18S	10,49
D5		wasser	N	FAM	18S	29,16
D7		target7.2	U	FAM	18S	14,44
D8		fillup.2	U	FAM	18S	10,61
E2		target8.2	U	FAM	18S	14,13
E3		reagent only.2	U	FAM	18S	11,37
E5		wasser	N	FAM	18S	30,01
E7		target8.2	U	FAM	18S	14,52
E8		reagent only.2	U	FAM	18S	12,17
F2		target13.2	U	FAM	18S	16,09
F3		untransfected.2	U	FAM	18S	13,03
F7		target13.2	U	FAM	18S	16,82
F8		untransfected.2	U	FAM	18S	13,02
G2		target14.2	U	FAM	18S	12,2
G7		target14.2	U	FAM	18S	13,19
H2		moxGFP.2	U	FAM	18S	13,95
H7		moxGFP.2	U	FAM	18S	14,16

Well		Sample name	Mean Ct	Std.Dev. Ct
A1		target7.2	30,94	0,98
A2		fillup.2	32,93	2,07
A4		target13.2	26,33	0,05
A5		untransfected.2	23,92	1,92
A6		target7.2	30,94	0,98
A7		fillup.2	32,93	2,07
A9		target13.2	26,33	0,05
A10		untransfected.2	23,92	1,92
B1		target8.2	28,66	0,69
B2		reagent only.2	32,7	1,86
B5		wasser	36,4	3,75
B6		target8.2	28,66	0,69
B7		reagent only.2	32,7	1,86
B9		target14.2	22,39	0
C1		target13.2	30	0,97

Monitoring

Well		Sample name	Mean Ct	Std.Dev. Ct
C2		untransfected.2	32,11	0
C4		moxGFP.2	23,53	0,68
C5		wasser	36,4	3,75
C6		target13.2	30	0,97
C7		untransfected.2	32,11	0
C9		moxGFP.2	23,53	0,68
D1		target14.2	28,89	0,22
D4		pScaf.2	24,3	0,6
D6		target14.2	28,89	0,22
D9		pScaf.2	24,3	0,6
E1		moxGFP.2	23,13	0,08
E4		BPK.2	25,72	0,54
E6		moxGFP.2	23,13	0,08
E9		BPK.2	25,72	0,54
F1		pScaf.2	32,98	1,05
F4		pY010.2	22,5	0,13
F5		wasser		
F6		pScaf.2	32,98	1,05
F9		pY010.2	22,5	0,13
G1		BPK.2	30,87	3,39
G3		target7.2	23,2	0,28
G4		fillup.2	20,81	0,21
G5		wasser	35,54	0
G6		BPK.2	30,87	3,39
G8		target7.2	23,2	0,28
G9		fillup.2	20,81	0,21
H1		pY010.2	32,29	0,39
H3		target8.2	24,49	0,1
H4		reagent only.2	21,88	0,22
H5		calibrator	23,23	0
H6		pY010.2	32,29	0,39
H8		target8.2	24,49	0,1
H9		reagent only.2	21,88	0,22
A3		pScaf.2	14,26	0,1
A8		pScaf.2	14,26	0,1
B3		BPK.2	16,07	0,42
B4		target14.2	15,78	5,36
B8		BPK.2	16,07	0,42
C3		pY010.2	12,93	0,57
C8		pY010.2	12,93	0,57
D2		target7.2	14,39	0,07
D3		fillup.2	10,55	0,08
D5		wasser	29,59	0,6
D7		target7.2	14,39	0,07
D8		fillup.2	10,55	0,08
E2		target8.2	14,33	0,28
E3		reagent only.2	11,77	0,57
E5		wasser	29,59	0,6
E7		target8.2	14,33	0,28
E8		reagent only.2	11,77	0,57
F2		target13.2	16,45	0,52
F3		untransfected.2	13,02	0
F7		target13.2	16,45	0,52

Well		Sample name	Mean Ct	Std.Dev. Ct
F8		untransfected.2	13,02	0
G2		target14.2	15,78	5,36
G7		target14.2	15,78	5,36
H2		moxGFP.2	14,05	0,15
H7		moxGFP.2	14,05	0,15

Monitoring

Tm

DIF - Threshold: 0

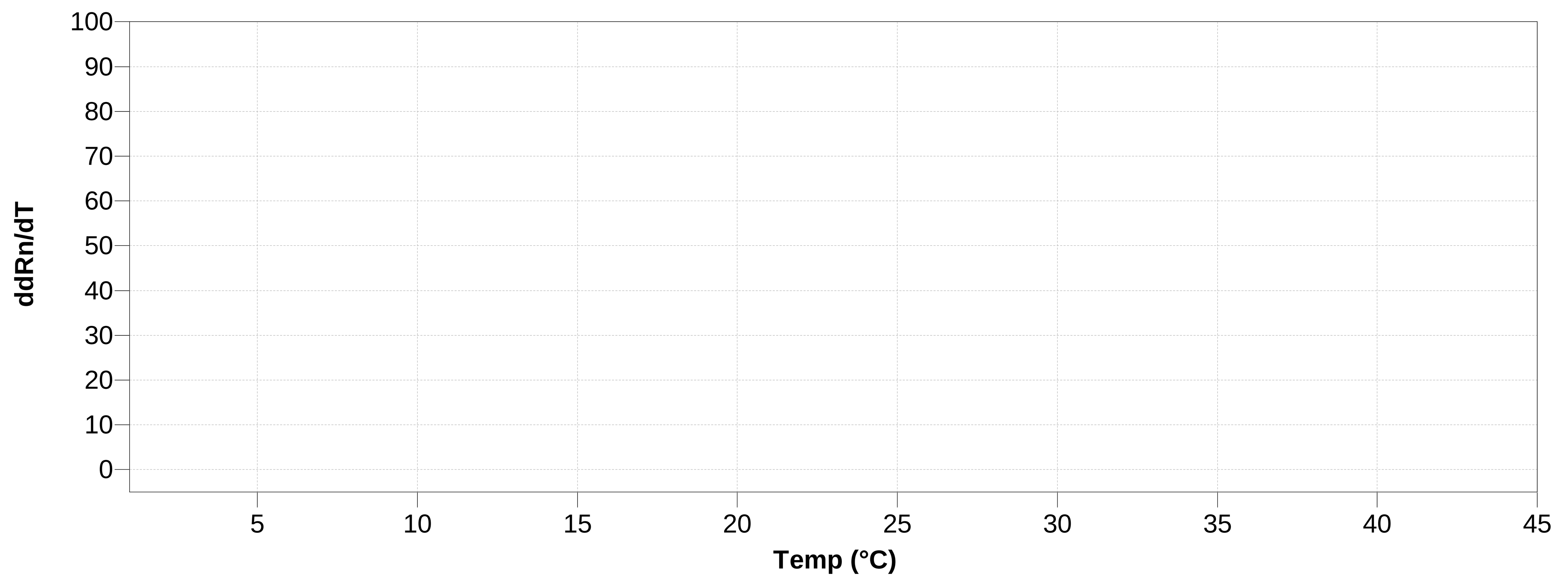

GOI -

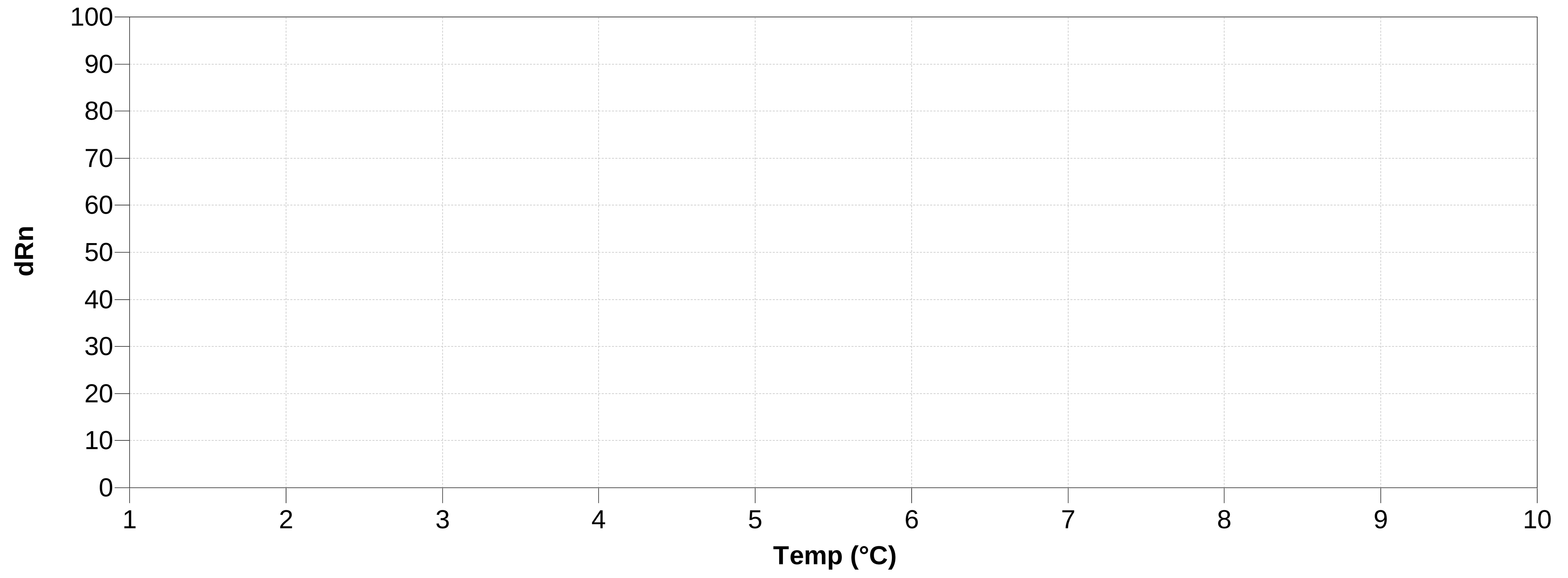

Well		Sample name	Sample type	Tm	Mean Tm	Std.Dev. Mean Tm
A1						
A2						
A3						
A4						
A5						
A6						
A7						
A8						
A9						
A10						
B1						
B2						
B3						
B4						
B5						
B6						
B7						
B8						
B9						
C1						
C2						
C3						
C4						
C5						

Monitoring

Well		Sample name	Sample type	Tm	Mean Tm	Std.Dev. Mean Tm
C6						
C7						
C8						
C9						
D1						
D2						
D3						
D4						
D5						
D6						
D7						
D8						
D9						
E1						
E2						
E3						
E4						
E5						
E6						
E7						
E8						
E9						
F1						
F2						
F3						
F4						
F5						
F6						
F7						
F8						
F9						
G1						
G2						
G3						
G4						
G5						
G6						
G7						
G8						
G9						
H1						
H2						
H3						
H4						
H5						
H6						
H7						
H8						
H9						

ddCt quantification

ddCt quantification

GOI - FAM Threshold: 3,08

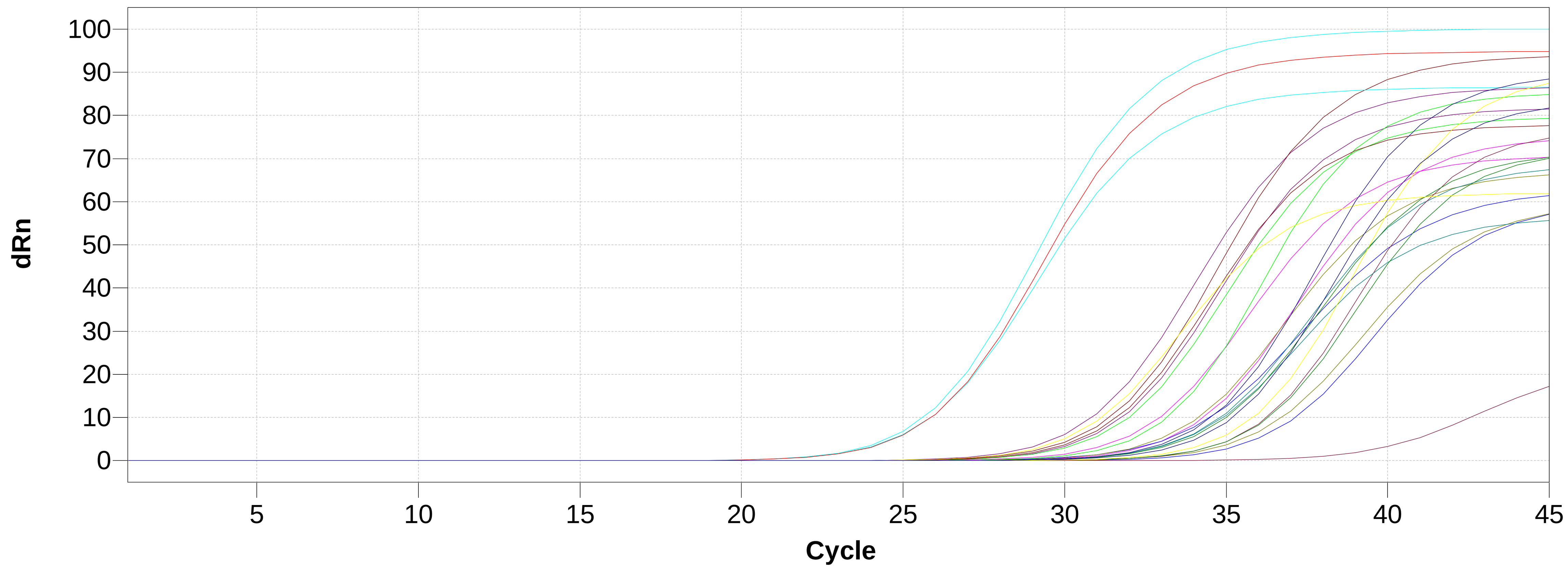

Ref.gene - FAM Threshold: 1,92

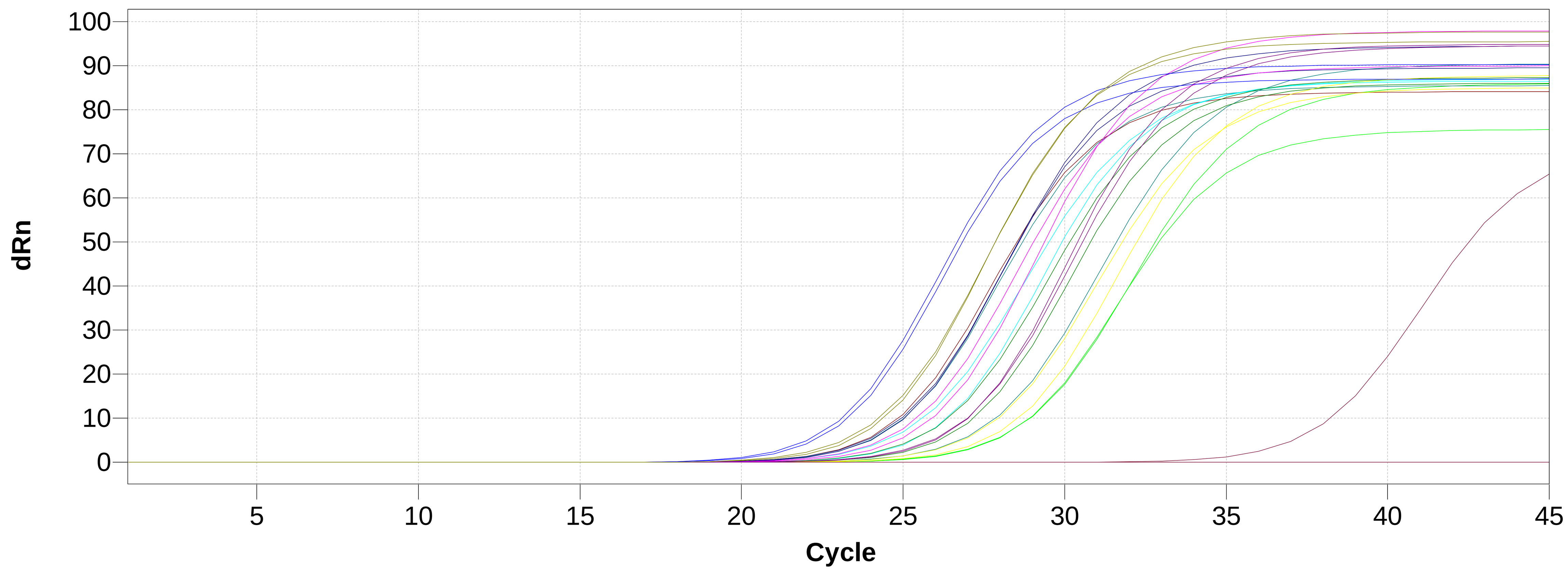

BAR

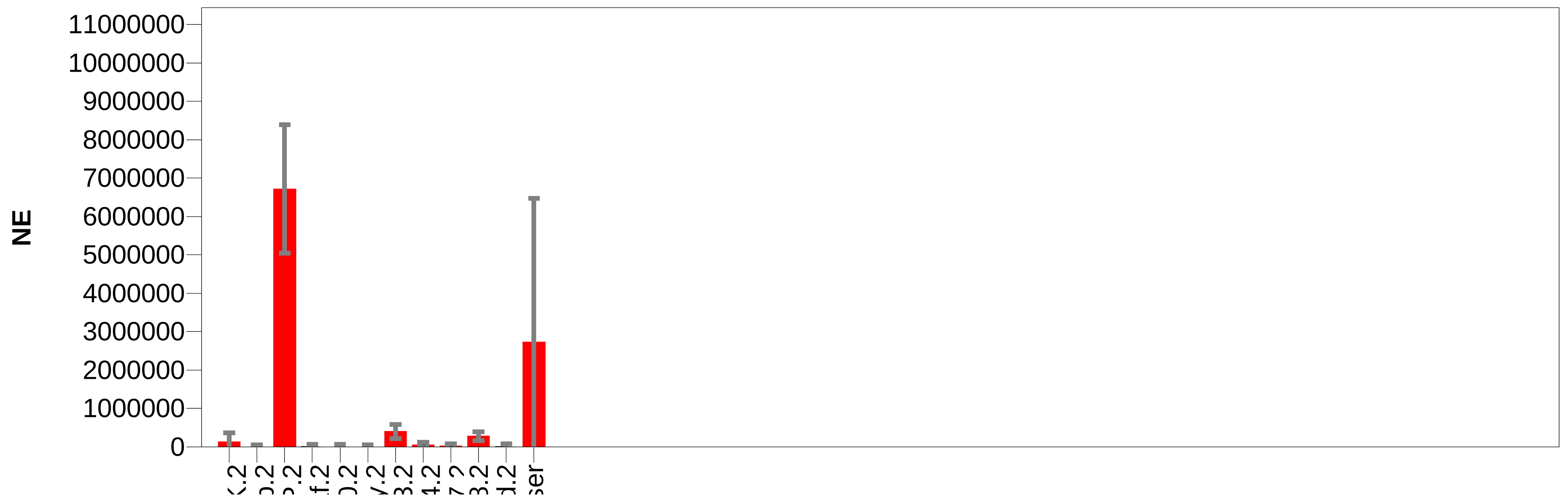

ddCt quantification

ddCt quantification

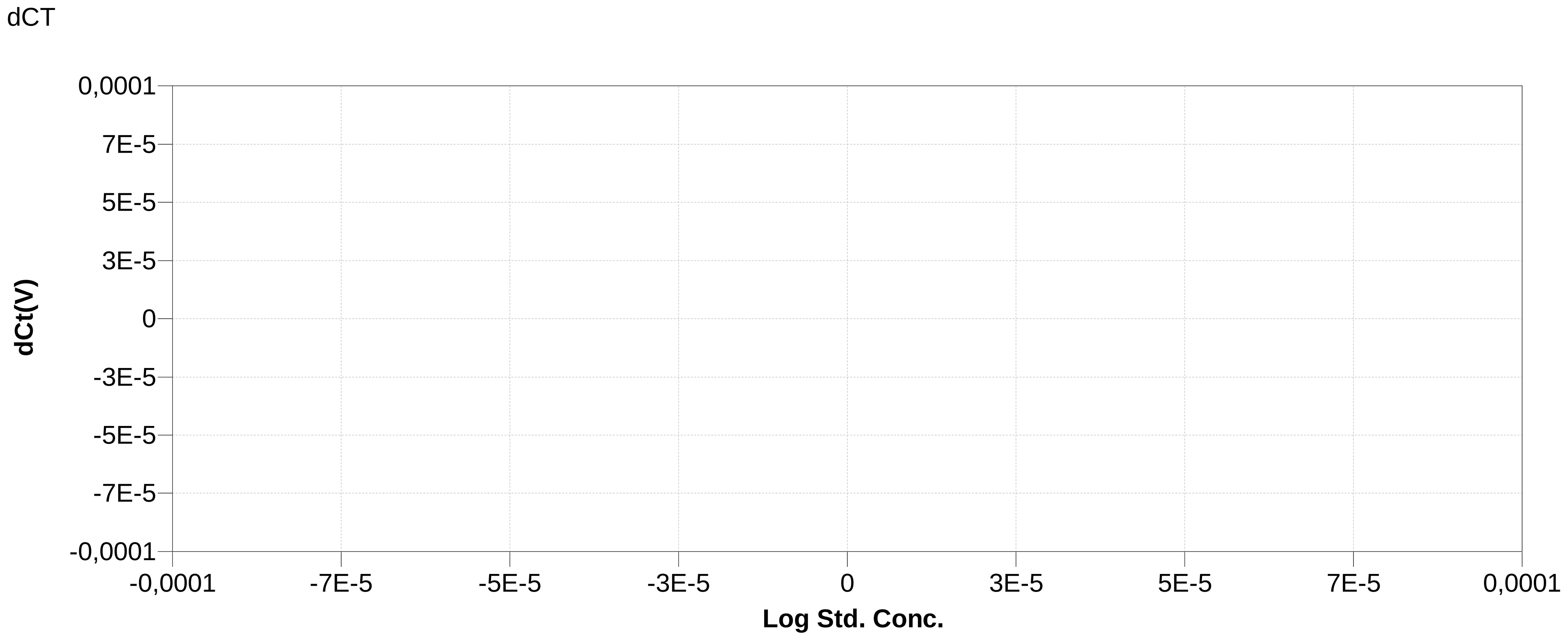

Well		Sample name	Sample type	GOI	Reference gene	Ct GOI
A1		target7.2	U	GFP		32,34
A2		fillup.2	U	GFP		35,15
A3		pScaf.2	U			
A4		target13.2	U		PPIA	
A5		untransfected.2	U		PPIA	
A6		target7.2	U	GFP		31,02
A7		fillup.2	U	GFP		32,28
A8		pScaf.2	U			
A9		target13.2	U		PPIA	
A10		untransfected.2	U		PPIA	
B1		target8.2	U	GFP		28,92
B2		reagent only.2	U	GFP		32,14
B3		BPK.2	U			
B4		target14.2	U			
B5		wasser	N	GFP		39,89
B6		target8.2	U	GFP		29,87
B7		reagent only.2	U	GFP		34,69
B8		BPK.2	U			
B9		target14.2	U		PPIA	
C1		target13.2	U	GFP		31,34
C2		untransfected.2	U	GFP		32,83
C3		pY010.2	U			
C4		moxGFP.2	U		PPIA	
C5		wasser	N	GFP		34,39
C6		target13.2	U	GFP		30,07
C7		untransfected.2	U	GFP		32,85
C8		pY010.2	U			
C9		moxGFP.2	U		PPIA	
D1		target14.2	U	GFP		29,43
D2		target7.2	U			
D3		fillup.2	U			
D4		pScaf.2	U		PPIA	
D5		wasser	N			
D6		target14.2	U	GFP		29,7
D7		target7.2	U			
D8		fillup.2	U			
D9		pScaf.2	U		PPIA	

ddCt quantification

Well		Sample name	Sample type	GOI	Reference gene	Ct GOI
E1		moxGFP.2	U	GFP		23,73
E2		target8.2	U			
E3		reagent only.2	U			
E4		BPK.2	U		PPIA	
E5		wasser	N			
E6		moxGFP.2	U	GFP		23,93
E7		target8.2	U			
E8		reagent only.2	U			
E9		BPK.2	U		PPIA	
F1		pScaf.2	U	GFP		34,39
F2		target13.2	U			
F3		untransfected.2	U			
F4		pY010.2	U		PPIA	
F5		wasser	N		PPIA	
F6		pScaf.2	U	GFP		33,02
F7		target13.2	U			
F8		untransfected.2	U			
F9		pY010.2	U		PPIA	
G1		BPK.2	U	GFP		34,02
G2		target14.2	U			
G3		target7.2	U		PPIA	
G4		fillup.2	U		PPIA	
G5		wasser	N		PPIA	
G6		BPK.2	U	GFP		29,19
G7		target14.2	U			
G8		target7.2	U		PPIA	
G9		fillup.2	U		PPIA	
H1		pY010.2	U	GFP		33,27
H2		moxGFP.2	U			
H3		target8.2	U		PPIA	
H4		reagent only.2	U		PPIA	
H5		calibrator	K	GFP		23,99
H6		pY010.2	U	GFP		32,64
H7		moxGFP.2	U			
H8		target8.2	U		PPIA	
H9		reagent only.2	U		PPIA	

Well		Sample name	Ct Ref. gene	Mean Ct GOI	Mean Ct Ref.gene	RQ GOI
A1		target7.2		31,68		0
A2		fillup.2		33,71		0
A3		pScaf.2				
A4		target13.2	26,3		26,33	
A5		untransfected.2	22,56		23,92	
A6		target7.2		31,68		0
A7		fillup.2		33,71		0
A8		pScaf.2				
A9		target13.2	26,37		26,33	
A10		untransfected.2	25,27		23,92	
B1		target8.2		29,39		0,02
B2		reagent only.2		33,42		0
B3		BPK.2				
B4		target14.2				
B5		wasser		37,14		0

ddCt quantification

Well		Sample name	Ct Ref. gene	Mean Ct GOI	Mean Ct Ref.gene	RQ GOI
B6		target8.2		29,39		0,02
B7		reagent only.2		33,42		0
B8		BPK.2				
B9		target14.2	22,39		22,39	
C1		target13.2		30,71		0,01
C2		untransfected.2		32,84		0
C3		pY010.2				
C4		moxGFP.2	24,01		23,53	
C5		wasser		37,14		0
C6		target13.2		30,71		0,01
C7		untransfected.2		32,84		0
C8		pY010.2				
C9		moxGFP.2	23,04		23,53	
D1		target14.2		29,56		0,02
D2		target7.2				
D3		fillup.2				
D4		pScaf.2	24,73		24,3	
D5		wasser				
D6		target14.2		29,56		0,02
D7		target7.2				
D8		fillup.2				
D9		pScaf.2	23,88		24,3	
E1		moxGFP.2		23,83		1,11
E2		target8.2				
E3		reagent only.2				
E4		BPK.2	26,1		25,72	
E5		wasser				
E6		moxGFP.2		23,83		1,11
E7		target8.2				
E8		reagent only.2				
E9		BPK.2	25,34		25,72	
F1		pScaf.2		33,71		0
F2		target13.2				
F3		untransfected.2				
F4		pY010.2	22,59		22,5	
F5		wasser	No Ct			
F6		pScaf.2		33,71		0
F7		target13.2				
F8		untransfected.2				
F9		pY010.2	22,4		22,5	
G1		BPK.2		31,6		0,01
G2		target14.2				
G3		target7.2	23		23,2	
G4		fillup.2	20,96		20,81	
G5		wasser	35,54		35,54	
G6		BPK.2		31,6		0,01
G7		target14.2				
G8		target7.2	23,4		23,2	
G9		fillup.2	20,66		20,81	
H1		pY010.2		32,96		0
H2		moxGFP.2				
H3		target8.2	24,56		24,49	
H4		reagent only.2	22,03		21,88	

ddCt quantification

Well		Sample name	Ct Ref. gene	Mean Ct GOI	Mean Ct Ref.gene	RQ GOI
H5		calibrator		23,99		1
H6		pY010.2		32,96		0
H7		moxGFP.2				
H8		target8.2	24,43		24,49	
H9		reagent only.2	21,72		21,88	

Well		Sample name	RQ Ref.gene	Norm. Expression	Std.Dev. RQ GOI	Std.Dev. RQ Ref.gene
A1		target7.2		23293,2731	0	
A2		fillup.2		1087,6648	0	
A3		pScaf.2				
A4		target13.2	0	401289,099		0
A5		untransfected.2	0	17075,7135		0
A6		target7.2		23293,2731	0	
A7		fillup.2		1087,6648	0	
A8		pScaf.2				
A9		target13.2	0	401289,099		0
A10		untransfected.2	0	17075,7135		0
B1		target8.2		278965,5031	0,01	
B2		reagent only.2		2790,6366	0	
B3		BPK.2				
B4		target14.2				
B5		wasser		2734055,8934	0	
B6		target8.2		278965,5031	0,01	
B7		reagent only.2		2790,6366	0	
B8		BPK.2				
B9		target14.2	0	57487,6033		0
C1		target13.2		401289,099	0,01	
C2		untransfected.2		17075,7135	0	
C3		pY010.2				
C4		moxGFP.2	0	6716050,719		0
C5		wasser		2734055,8934	0	
C6		target13.2		401289,099	0,01	
C7		untransfected.2		17075,7135	0	
C8		pY010.2				
C9		moxGFP.2	0	6716050,719		0
D1		target14.2		57487,6033	0	
D2		target7.2				
D3		fillup.2				
D4		pScaf.2	0	12250,2091		0
D5		wasser				
D6		target14.2		57487,6033	0	
D7		target7.2				
D8		fillup.2				
D9		pScaf.2	0	12250,2091		0
E1		moxGFP.2		6716050,719	0,11	
E2		target8.2				
E3		reagent only.2				
E4		BPK.2	0	140742,1964		0
E5		wasser				
E6		moxGFP.2		6716050,719	0,11	
E7		target8.2				
E8		reagent only.2				
E9		BPK.2	0	140742,1964		0

ddCt quantification

Well		Sample name	RQ Ref.gene	Norm. Expression	Std.Dev. RQ GOI	Std.Dev. RQ Ref.gene
F1		pScaf.2		12250,2091	0	
F2		target13.2				
F3		untransfected.2				
F4		pY010.2	0	5885,0672		0
F5		wasser				
F6		pScaf.2		12250,2091	0	
F7		target13.2				
F8		untransfected.2				
F9		pY010.2	0	5885,0672		0
G1		BPK.2		140742,1964	0,01	
G2		target14.2				
G3		target7.2	0	23293,2731		0
G4		fillup.2	0	1087,6648		0
G5		wasser	0	2734055,8934		0
G6		BPK.2		140742,1964	0,01	
G7		target14.2				
G8		target7.2	0	23293,2731		0
G9		fillup.2	0	1087,6648		0
H1		pY010.2		5885,0672	0	
H2		moxGFP.2				
H3		target8.2	0	278965,5031		0
H4		reagent only.2	0	2790,6366		0
H5		calibrator			0	
H6		pY010.2		5885,0672	0	
H7		moxGFP.2				
H8		target8.2	0	278965,5031		0
H9		reagent only.2	0	2790,6366		0

Well		Sample name	Std.Dev. Norm. Expression	dCt (Ref.Gen – GOI)
A1		target7.2	15789,4099	-8,48
A2		fillup.2	1536,7306	-12,9
A3		pScaf.2		
A4		target13.2		
A5		untransfected.2		
A6		target7.2	15789,4099	-8,48
A7		fillup.2	1536,7306	-12,9
A8		pScaf.2		
A9		target13.2		
A10		untransfected.2		
B1		target8.2	131504,6578	-4,9
B2		reagent only.2	3508,3471	-11,54
B3		BPK.2		
B4		target14.2		
B5		wasser	7363107,3909	-1,6
B6		target8.2	131504,6578	-4,9
B7		reagent only.2	3508,3471	-11,54
B8		BPK.2		
B9		target14.2		
C1		target13.2	249838,6651	-4,37
C2		untransfected.2	22696,8018	-8,93
C3		pY010.2		
C4		moxGFP.2		
C5		wasser	7363107,3909	-1,6

ddCt quantification

Well		Sample name	Std.Dev. Norm. Expression	dCt (Ref.Gen – GOI)
C6		target13.2	249838,6651	-4,37
C7		untransfected.2	22696,8018	-8,93
C8		pY010.2		
C9		moxGFP.2		
D1		target14.2	7651,5412	-7,18
D2		target7.2		
D3		fillup.2		
D4		pScaf.2		
D5		wasser		
D6		target14.2	7651,5412	-7,18
D7		target7.2		
D8		fillup.2		
D9		pScaf.2		
E1		moxGFP.2	3238274,5057	-0,31
E2		target8.2		
E3		reagent only.2		
E4		BPK.2		
E5		wasser		
E6		moxGFP.2	3238274,5057	-0,31
E7		target8.2		
E8		reagent only.2		
E9		BPK.2		
F1		pScaf.2	9692,7661	-9,41
F2		target13.2		
F3		untransfected.2		
F4		pY010.2		
F5		wasser		
F6		pScaf.2	9692,7661	-9,41
F7		target13.2		
F8		untransfected.2		
F9		pY010.2		
G1		BPK.2	336778,829	-5,88
G2		target14.2		
G3		target7.2		
G4		fillup.2		
G5		wasser		
G6		BPK.2	336778,829	-5,88
G7		target14.2		
G8		target7.2		
G9		fillup.2		
H1		pY010.2	1894,568	-10,46
H2		moxGFP.2		
H3		target8.2		
H4		reagent only.2		
H5		calibrator		
H6		pY010.2	1894,568	-10,46
H7		moxGFP.2		
H8		target8.2		
H9		reagent only.2		
